# Supplementary material for: Determinants of trafficking, conduction, and disease within a K+ channel revealed through multiparametric deep mutational scanning
Source: eLife. 2022 May 31;11:e76903. doi: 10.7554/eLife.76903 (PMC9273215; doi:10.7554/eLife.76903)
Supplement: Source data 1. [file elife-76903-data1.zip › SourceData/SourceData.pdf]

Coyote-Maestas 2022 Source Data

Willow Coyote-Maestas, David Nedrud, Yungui He, Daniel Schmidt

2022-04-25

Setup

```
library(ggplot2)
library(scales)
library(reshape2)
library(colorspace)
library(dplyr)
```

```
##
## Attaching package: 'dplyr'
## The following objects are masked from 'package:stats':
##
##     filter, lag
## The following objects are masked from 'package:base':
##
##     intersect, setdiff, setequal, union
```

```
library(tidyr)
```

```
##
## Attaching package: 'tidyr'
## The following object is masked from 'package:reshape2':
##
##     smiths
```

```
library(ggpubr)
library(qwraps2)
```

```
##
## Attaching package: 'qwraps2'
## The following objects are masked from 'package:ggpubr':
##
##     mean_ci, mean_sd, median_iqr
```

```
library(kableExtra)
```

```
##
## Attaching package: 'kableExtra'
## The following object is masked from 'package:dplyr':
##
##     group_rows
```

```
library(bio3d)
library(ComplexHeatmap)
```

```
## Loading required package: grid
```

```
## ========================================
## ComplexHeatmap version 2.8.0
## Bioconductor page: http://bioconductor.org/packages/ComplexHeatmap/
## Github page: https://github.com/jokergoo/ComplexHeatmap
## Documentation: http://jokergoo.github.io/ComplexHeatmap-reference
##
## If you use it in published research, please cite:
## Gu, Z. Complex heatmaps reveal patterns and correlations in multidimensional
##   genomic data. Bioinformatics 2016.
##
## The new InteractiveComplexHeatmap package can directly export static
## complex heatmaps into an interactive Shiny app with zero effort. Have a try!
##
## This message can be suppressed by:
##   suppressPackageStartupMessages(library(ComplexHeatmap))
## ========================================
```

```
library(matrixStats)
```

```
##
## Attaching package: 'matrixStats'
```

```
## The following object is masked from 'package:dplyr':
##
##     count
```

```
setwd(
  "/Volumes/GoogleDrive/Shared drives/Schmidt Lab/PLANNING/Kir2.1_DMS/REVISIONS/SourceData"
)
fig_path = './figure_output/'

set.seed(42)
```

Helper Functions

```
'%!in%' = function(x, y)
  ! ('%in%'(x, y))

range01 = function(x, remove.na = F) {
  if (remove.na) {
    (x - min(x, na.rm = T)) / (max(x, na.rm = T) - min(x, na.rm = T))
  }
  else{
    (x - min(x)) / (max(x) - min(x))
  }
}

frac_perc = function(value) {
  round(sum(as.numeric(value) >= 30, na.rm = T) / n_pos * 100, digits = 1)
}
```

```r
cor.prob <- function(X, dfr = nrow(X) - 2) {
  R <- cor(X)
  above <- row(R) < col(R)
  r2 <- R[above] ^ 2
  Fstat <- r2 * dfr / (1 - r2)
  R[above] <- 1 - pf(Fstat, 1, dfr)
  R
}
```

##BASELINE

```r
#Number of Kir2.1 positions, not considering FLAG tag res 116-124
n_pos = length(c(2:115, 124:400)) * 19

baseline = openxlsx::read.xlsx('./input_data/baseline_single_mutations.xlsx', sheet = 5)
baseline = aggregate(. ~ mutation, baseline[, -c(1, 3)], sum)
baseline$mutation = factor(
  baseline$mutation,
  levels = c(
    'H',
    'K',
    'R',
    'D',
    'E',
    'C',
    'M',
    'N',
    'Q',
    'S',
    'T',
    'A',
    'I',
    'L',
    'V',
    'W',
    'F',
    'Y',
    'G',
    'P'
  )
)
baseline = baseline[-1, ]

baseline = melt(
  baseline,
  id.vars = 'mutation',
  variable.name = 'position',
  value.name = 'count'
)
baseline$position = as.numeric(baseline$position)

irk = openxlsx::read.xlsx('./input_data/IRK_alignment_master_updated020521.xlsx')
idx = match(baseline$position, irk$Kir2_1_FLAG_Resno_mus)
baseline$wt_resid = irk$Kir2_1_FLAG_Resid_mus[idx]
```

```r
baseline$is.wt = baseline$mutation == baseline$wt_resid
baseline[baseline$count == 0, 'count'] = NA

baseline_var_det = nrow(baseline[baseline$is.wt == F &
                                   baseline$position %in% c(2:115, 124:400) &
                                   !is.na(baseline$count), ])
baseline_var_det = nrow(baseline[baseline$is.wt == F &
                                   baseline$position %in% c(2:115, 124:400) &
                                   baseline$count > 20, ])
```

```r
paste0(
  'Baseline present for ',
  baseline_var_det,
  ' variants (',
  round(baseline_var_det / n_pos * 100, 1),
  '%).'
)
```

```
## [1] "Baseline present for 6702 variants (90.2%)."
```

```r
paste0(
  '>= 20 read count for ',
  sum(baseline$count >=20, na.rm = T),
  ' variants (',
  round(sum(baseline$count >=20, na.rm = T) / n_pos * 100, 1),
  '%).'
)
```

```
## [1] ">= 20 read count for 6610 variants (89%)."
```

##SURFACE ##Surface Read Count Data Intake

```r
d_neg1 = read.delim('./enrich_surface/tsv/neg1_lib/main_synonymous_counts.tsv')
colnames(d_neg1) = c('variant', 'count1')
d_neg2 = read.delim('./enrich_surface/tsv/neg2_lib/main_synonymous_counts.tsv')
colnames(d_neg2) = c('variant', 'count2')
d_neg = merge(d_neg1, d_neg2, all = T)
d_neg$cond = rep('neg', nrow(d_neg))

d_high1 = read.delim('./enrich_surface/tsv/high1_lib/main_synonymous_counts.tsv')
colnames(d_high1) = c('variant', 'count1')
d_high2 = read.delim('./enrich_surface/tsv/high2_lib/main_synonymous_counts.tsv')
colnames(d_high2) = c('variant', 'count2')
d_high = merge(d_high1, d_high2, all = T)
d_high$cond = rep('high', nrow(d_high))

d_low1 = read.delim('./enrich_surface/tsv/low1_lib/main_synonymous_counts.tsv')
colnames(d_low1) = c('variant', 'count1')
d_low2 = read.delim('./enrich_surface/tsv/low2_lib/main_synonymous_counts.tsv')
colnames(d_low2) = c('variant', 'count2')
d_low = merge(d_low1, d_low2, all = T)
d_low$cond = rep('low', nrow(d_low))

d_up1 = read.delim('./enrich_surface/tsv/up1_lib/main_synonymous_counts.tsv')
colnames(d_up1) = c('variant', 'count1')
d_up2 = read.delim('./enrich_surface/tsv/up2_lib/main_synonymous_counts.tsv')
```

```
colnames(d_up2) = c('variant', 'count2')
d_up = merge(d_up1, d_up2, all = T)
d_up$cond = rep('up', nrow(d_up))

d_neg_rep = ggscatter(
  d_neg,
  x = 'count1',
  y = 'count2',
  add = 'reg.line',
  conf.int = T,
  add.params = list(color = 'red'),
  cor.coef = T,
  cor.method = 'spearman',
  title = 'neg'
) +
  scale_y_log10() + scale_x_log10()

d_low_rep = ggscatter(
  d_low,
  x = 'count1',
  y = 'count2',
  add = 'reg.line',
  conf.int = T,
  add.params = list(color = 'red'),
  cor.coef = T,
  cor.method = 'spearman',
  title = 'low'
) +
  scale_y_log10() + scale_x_log10()

d_up_rep = ggscatter(
  d_up,
  x = 'count1',
  y = 'count2',
  add = 'reg.line',
  conf.int = T,
  add.params = list(color = 'red'),
  cor.coef = T,
  cor.method = 'spearman',
  title = 'up'
) +
  scale_y_log10() + scale_x_log10()

d_high_rep = ggscatter(
  d_high,
  x = 'count1',
  y = 'count2',
  add = 'reg.line',
  conf.int = T,
  add.params = list(color = 'red'),
  cor.coef = T,
  cor.method = 'spearman',
  title = 'high'
```

```
) +
  scale_y_log10() + scale_x_log10()
```

##Surface Read count Stats

```
d_count = rbind(d_neg, d_low, d_up, d_high)
d_count_melt = melt(d_count, id.vars = c('variant', 'cond'))
d_count_melt$cond = factor(d_count_melt$cond, levels = c('neg', 'low', 'up', 'high'))
```

##Surface Read Count Summary in Table

```
d_count_melt %>%
  group_by(cond, variable) %>%
  summarise(
    median = median(as.numeric(value), na.rm = T),
    mean = mean(as.numeric(value), na.rm = T),
    n = n()
  )
```

```
## `summarise()` has grouped output by 'cond'. You can override using the
## `.groups` argument.
```

```
## # A tibble: 8 x 5
## # Groups:   cond [4]
##   cond  variable median  mean     n
##   <fct> <fct>     <dbl> <dbl> <int>
## 1 neg   count1     103   342.  7282
## 2 neg   count2     174   345.  7282
## 3 low   count1     187   316.  7305
## 4 low   count2     179   307.  7305
## 5 up    count1     160.  310.  7175
## 6 up    count2     160   310.  7175
## 7 high  count1     130   301.  6975
## 8 high  count2     123   286.  6975
```

```
read_count_summary =
  list(
    "Read Count Statistics" =
      list(
        "median" = ~ median(as.numeric(value), na.rm = T),
        'mean' = ~ mean(as.numeric(value), na.rm = T),
        '>30-fold [%]' = ~ frac_perc(value)
      )
  )
```

##Processed Surface Data Intake

```
kir21_surf = read.csv(
  './input_data/surface_mod.csv',
  header = TRUE,
  sep = ",",
  stringsAsFactors = FALSE
)
kir21_surf$mutation = bio3d::aa321(toupper(kir21_surf$mutation))
plot_data = melt(
  acast(
    data = kir21_surf,
```

```r
    mutation ~ position,
    value.var = 'surface_score',
    fill = NA_real_,
    fun.aggregate = I
  )
)
colnames(plot_data) = c("mutation", 'position', 'surface_score')
kir21_surf = merge(plot_data, kir21_surf, all.x = T)

idx = match(kir21_surf$position, irk$Kir2_1_FLAG_Resno_mus)
kir21_surf$wt_resid = irk$Kir2_1_FLAG_Resid_mus[idx]
kir21_surf$is.wt = kir21_surf$mutation == kir21_surf$wt_resid

kir21_surf$mutation = factor(
  kir21_surf$mutation,
  levels = c(
    'H',
    'K',
    'R',
    'D',
    'E',
    'C',
    'M',
    'N',
    'Q',
    'S',
    'T',
    'A',
    'I',
    'L',
    'V',
    'W',
    'F',
    'Y',
    'G',
    'P'
  )
)

kir21_surf$trust = ifelse(kir21_surf$surface_SE > 1, 'X', NA)

surf_var_det = nrow(kir21_surf[kir21_surf$is.wt == F &
                                 kir21_surf$position %in% c(2:115, 125:400) &
                                 !is.na(kir21_surf$surface_score), ])
```

```r
paste0(
  'Surface Trafficking determined for ',
  surf_var_det,
  ' variants (',
  round(surf_var_det / n_pos * 100, 1),
  '%).'
)
```

```
## [1] "Surface Trafficking determined for 6898 variants (92.9%)."
```

##FUNCTION #Function Read Count Data Intake

```
f_neg1 = read.delim('./enrich_function/tsv/neg_lib/main_synonymous_counts.tsv')
colnames(f_neg1) = c('variant', 'count1')
f_neg2 = read.delim('./enrich_function/tsv/neg2_lib/main_synonymous_counts.tsv')
colnames(f_neg2) = c('variant', 'count2')
f_neg = merge(f_neg1, f_neg2, all = T)
f_neg$cond = rep('neg', nrow(f_neg))

f_high1 = read.delim('./enrich_function/tsv/high_lib/main_synonymous_counts.tsv')
colnames(f_high1) = c('variant', 'count1')
f_high2 = read.delim('./enrich_function/tsv/high2_lib/main_synonymous_counts.tsv')
colnames(f_high2) = c('variant', 'count2')
f_high = merge(f_high1, f_high2, all = T)
f_high$cond = rep('high', nrow(f_high))

f_low1 = read.delim('./enrich_function/tsv/low_lib/main_synonymous_counts.tsv')
colnames(f_low1) = c('variant', 'count1')
f_low2 = read.delim('./enrich_function/tsv/low2_lib/main_synonymous_counts.tsv')
colnames(f_low2) = c('variant', 'count2')
f_low = merge(f_low1, f_low2, all = T)
f_low$cond = rep('low', nrow(f_low))


f_neg_plot = ggscatter(
  f_neg,
  x = 'count1',
  y = 'count2',
  add = 'reg.line',
  conf.int = T,
  add.params = list(color = 'red'),
  cor.coef = T,
  cor.method = 'spearman',
  title = 'neg'
) +
  scale_y_log10() + scale_x_log10()


f_low_plot = ggscatter(
  f_low,
  x = 'count1',
  y = 'count2',
  add = 'reg.line',
  conf.int = T,
  add.params = list(color = 'red'),
  cor.coef = T,
  cor.method = 'spearman',
  title = 'low'
) +
  scale_y_log10() + scale_x_log10()

f_high_plot = ggscatter(
  f_high,
  x = 'count1',
```

```
  y = 'count2',
  add = 'reg.line',
  conf.int = T,
  add.params = list(color = 'red'),
  cor.coef = T,
  cor.method = 'spearman',
  title = 'high'
) +
  scale_y_log10() + scale_x_log10()
```

#Function Read Count Stats

```
f_count = rbind(f_neg, f_low, f_high)
f_count_melt = melt(f_count, id.vars = c('variant', 'cond'))
f_count_melt$cond = factor(f_count_melt$cond, levels = c('neg', 'low', 'high'))
```

#Function Read Count Summary in Table

```
f_count_melt %>%
  group_by(cond, variable) %>%
  summarise(
    median = median(as.numeric(value), na.rm = T),
    mean = mean(as.numeric(value), na.rm = T),
    n = n()
  )
```

```
## `summarise()` has grouped output by 'cond'. You can override using the
## `.groups` argument.
```

```
## # A tibble: 6 x 5
## # Groups:   cond [3]
##   cond  variable median  mean     n
##   <fct> <fct>     <dbl> <dbl> <int>
## 1 neg   count1      178  282.  7168
## 2 neg   count2      165  297.  7168
## 3 low   count1      162  248.  7284
## 4 low   count2      167  276.  7284
## 5 high  count1      168  261.  7320
## 6 high  count2      182  296.  7320
```

```
read_count_summary =
  list(
    "Read Count Statistics Function" =
      list(
        "median" = ~ median(as.numeric(value), na.rm = T),
        'mean' = ~ mean(as.numeric(value), na.rm = T),
        '>30-fold [%]' = ~ frac_perc(value)
      )
  )
```

#Processed Function Data Intake

```
kir21_func = read.csv(
  './input_data/function_mod.csv',
  header = TRUE,
  sep = ",",
  stringsAsFactors = FALSE
```

```
)

kir21_func$mutation = bio3d::aa321(toupper(kir21_func$mutation))
plot_data = melt(
  acast(
    data = kir21_func,
    mutation ~ position,
    value.var = 'function_score',
    fill = NA_real_,
    fun.aggregate = I
  )
)
colnames(plot_data) = c("mutation", 'position', 'function_score')
kir21_func = merge(plot_data, kir21_func, all.x = T)

idx = match(kir21_func$position, irk$Kir2_1_FLAG_Resno_mus)
kir21_func$wt_resid = irk$Kir2_1_FLAG_Resid_mus[idx]
kir21_func$is.wt = kir21_func$mutation == kir21_func$wt_resid

kir21_func$mutation = factor(
  kir21_func$mutation,
  levels = c(
    'H',
    'K',
    'R',
    'D',
    'E',
    'C',
    'M',
    'N',
    'Q',
    'S',
    'T',
    'A',
    'I',
    'L',
    'V',
    'W',
    'F',
    'Y',
    'G',
    'P'
  )
)

kir21_func$trust = ifelse(kir21_func$function_SE > 1, 'X', NA)

func_var_det = nrow(kir21_func[kir21_func$is.wt == F &
                                 kir21_func$position %in% c(2:115, 125:400) &
                                 !is.na(kir21_func$function_score), ])
```

```
paste0(
  'Function determined for ',
  func_var_det,
```

```r
  ' variants (',
  round(func_var_det / n_pos * 100, 1),
  '%).'
)
```

```
## [1] "Function determined for 6944 variants (93.5%)."
```

#Measures of Surface and Function Fitness Error

```r
xd_surf = density(kir21_surf$surface_score, bw = .15, na.rm = T)
correction_factor_surf = xd_surf$x[xd_surf$y == max(xd_surf$y)]
measurement_range_surf = abs(xd_surf$x[xd_surf$y == max(xd_surf$y[xd_surf$x < -4])])
```

```r
paste0(
  "Median fitness error compared to measurement range: ",
  round(
    median(kir21_surf$surface_SE, na.rm = T) / measurement_range_surf * 100,
    1
  ),
  "%"
)
```

```
## [1] "Median fitness error compared to measurement range: 7.2%"
```

```r
paste0(
  "WT IQR compared to measurement range: ",
  round(IQR(kir21_surf[kir21_surf$is.wt == T, 'surface_score'], na.rm = T) /
          measurement_range_surf * 100, 1),
  "%"
)
```

```
## [1] "WT IQR compared to measurement range: 10.6%"
```

```r
paste0(
  "WT standard deviation compared to measurement range: ",
  round(sd(kir21_surf[kir21_surf$is.wt == T, 'surface_score'], na.rm = T) /
          measurement_range_surf * 100, 1),
  "%"
)
```

```
## [1] "WT standard deviation compared to measurement range: 7.6%"
```

```r
surf_median_WT = round(median(kir21_surf[kir21_surf$is.wt == T, 'surface_score'],
                              na.rm = T), 2)
surf_SD_WT = round(sd(kir21_surf[kir21_surf$is.wt == T, 'surface_score'],
                      na.rm = T), 2)
surf_median_MUT = round(median(kir21_surf[kir21_surf$is.wt == F, 'surface_score'],
                               na.rm = T), 2)
surf_SD_MUT = round(sd(kir21_surf[kir21_surf$is.wt == F, 'surface_score'],
                       na.rm = T), 2)
```

```r
paste0("Median WT: ", surf_median_WT)
```

```
## [1] "Median WT: -0.1"
```

```r
paste0("Median mutant: ", surf_median_MUT)
```

```
## [1] "Median mutant: -0.17"
```

```r
paste0("Standard Dev WT: ", surf_SD_WT)
```

```
## [1] "Standard Dev WT: 0.4"
```

```r
paste0("Standard Dev mutant: ", surf_SD_MUT)
```

```
## [1] "Standard Dev mutant: 2.18"
```

```r
surf_wt_quant = quantile(kir21_surf[kir21_surf$is.wt == T, 'surface_score'],
                         probs = seq(0, 1, 0.1), na.rm = T)[c(2, 10)]
surf_mut_quant = quantile(kir21_surf[kir21_surf$is.wt == F, 'surface_score'],
                          probs = seq(0, 1, 0.1), na.rm = T)[c(2, 10)]
```

```r
paste0("lower 10% percentile tail diff abs(WT-mut) :",
       round(abs(surf_wt_quant[1] - surf_mut_quant[1]), 2))
```

```
## [1] "lower 10% percentile tail diff abs(WT-mut) :4.1"
```

```r
paste0("upper 10% percentile tail diff abs(WT-mut) :",
       round(abs(surf_wt_quant[2] - surf_mut_quant[2]), 2))
```

```
## [1] "upper 10% percentile tail diff abs(WT-mut) :0.94"
```

```r
xd_func = density(kir21_func$function_score, bw = .15, na.rm = T)
correction_factor_func = xd_func$x[xd_func$y == max(xd_func$y)]
measurement_range_func = abs(xd_func$x[xd_func$y == max(xd_func$y[xd_func$x < -2.5])])
```

```r
paste0(
  "Median fitness error compared to measurement range: ",
  round(
    median(kir21_func$function_SE, na.rm = T) / measurement_range_func * 100,
    1
  ),
  "%"
)
```

```
## [1] "Median fitness error compared to measurement range: 9.4%"
```

```r
paste0(
  "WT IQR compared to measurement range: ",
  round(IQR(kir21_func[kir21_func$is.wt == T, 'function_score'], na.rm = T) /
          measurement_range_func * 100, 1),
  "%"
)
```

```
## [1] "WT IQR compared to measurement range: 12.9%"
```

```r
paste0(
  "WT standard deviation compared to measurement range: ",
  round(sd(kir21_func[kir21_func$is.wt == T, 'function_score'], na.rm = T) /
          measurement_range_func * 100, 1),
  "%"
)
```

```
## [1] "WT standard deviation compared to measurement range: 9.7%"
```

```r
func_median_WT = round(median(kir21_func[kir21_func$is.wt == T, 'function_score'],
                              na.rm = T), 2)
func_SD_WT = round(sd(kir21_func[kir21_func$is.wt == T, 'function_score'],
```

```r
                        na.rm = T), 2)
func_median_MUT = round(median(kir21_func[kir21_func$is.wt == F, 'function_score'],
                                  na.rm = T), 2)
func_SD_MUT = round(sd(kir21_func[kir21_func$is.wt == F, 'function_score'],
                         na.rm = T), 2)
```

```r
paste0("Median WT: ", func_median_WT)
```

```
## [1] "Median WT: 0.07"
```

```r
paste0("Median mutant: ", func_median_MUT)
```

```
## [1] "Median mutant: 0.04"
```

```r
paste0("Standard Dev WT: ", func_SD_WT)
```

```
## [1] "Standard Dev WT: 0.24"
```

```r
paste0("Standard Dev mutant: ", func_SD_MUT)
```

```
## [1] "Standard Dev mutant: 0.6"
```

```r
func_wt_quant = quantile(kir21_func[kir21_func$is.wt == T, 'function_score'],
                         probs = seq(0, 1, 0.1), na.rm = T)[c(2, 10)]
func_mut_quant = quantile(kir21_func[kir21_func$is.wt == F, 'function_score'],
                          probs = seq(0, 1, 0.1), na.rm = T)[c(2, 10)]
```

```r
paste0("lower 10% percentile tail diff abs(WT-mut) :",
       round(abs(func_wt_quant[1] - func_mut_quant[1]), 2))
```

```
## [1] "lower 10% percentile tail diff abs(WT-mut) :0.53"
```

```r
paste0("upper 10% percentile tail diff abs(WT-mut) :",
       round(abs(func_wt_quant[2] - func_mut_quant[2]), 2))
```

```
## [1] "upper 10% percentile tail diff abs(WT-mut) :0.35"
```

#NA correlation Baseline, Surface, Function

```r
baseline_NA = baseline[baseline$position %in% c(2:115, 125:400) &
                         !baseline$is.wt & is.na(baseline$count),
                       c('mutation', 'position')]
baseline_NA$cond = rep(1, nrow(baseline_NA))
baseline_NA$baseline = rep(1, nrow(baseline_NA))
surface_NA = kir21_surf[!kir21_surf$is.wt &
                          is.na(kir21_surf$surface_score),
                        c('mutation', 'position')]
surface_NA$cond = rep(10, nrow(surface_NA))
surface_NA$surface = rep(1, nrow(surface_NA))
func_NA = kir21_func[!kir21_func$is.wt &
                       is.na(kir21_func$function_score),
                     c('mutation', 'position')]
func_NA$cond = rep(100, nrow(func_NA))
func_NA$func = rep(1, nrow(func_NA))
distr_NA = rbind(baseline_NA[, -4], surface_NA[, -4], func_NA[, -4])


distr_NA_matrix = dcast(
  distr_NA,
```

```
  mutation ~ position,
  fun.aggregate = function(x)
    as.character(sum(x)),
  value.var = 'cond'
)
distr_NA_plot = melt(
  distr_NA_matrix,
  id.vars = 'mutation',
  variable.name = 'position',
  value.name = 'cond_int'
)
distr_NA_plot$cond_int = factor(distr_NA_plot$cond_int,
                                levels =
                                  c('0', '1', '101', '111',
                                    '10', '11', '110',
                                    '100'))

list_NA = list(
  baseline = as.character(interaction(
    baseline_NA$mutation, as.character(baseline_NA$position)
  )),
  surface = as.character(interaction(
    surface_NA$mutation, as.character(surface_NA$position)
  )),
  func = as.character(interaction(
    func_NA$mutation, as.character(func_NA$position)
  ))
)

cor_NA = merge(baseline_NA[, -3], surface_NA[, -3], all = T)
cor_NA = merge(cor_NA, func_NA[, -3], all = T)
cor_NA[is.na(cor_NA)] = 0
```

##CLINVAR & GNOMAD #data intake

```
clinvar = openxlsx::read.xlsx('./input_data/KCNJ_Clinvar_0325322(edited_annotations).xlsx')
gnomad = openxlsx::read.xlsx('./input_data/KCNJ_Gnomad_0325322.xlsx')

dbvar = merge(
  gnomad,
  clinvar,
  by.x = c('Gene', 'Mutation'),
  by.y = c('Gene', 'Mutation'),
  all = T
)
dbvar$Source = rep(NA, nrow(dbvar))
dbvar$Source[dbvar$Source1 == 'Clinvar' &
               is.na(dbvar$Source2)] = 'Clinvar only'
dbvar$Source[dbvar$Source2 == 'Gnomad' &
               is.na(dbvar$Source1)] = 'Gnomad only'
dbvar$Source[dbvar$Source1 == 'Clinvar' &
               dbvar$Source2 == 'Gnomad'] = 'Clinvar & Gnomad'
dbvar$Source = as.factor(dbvar$Source)
```

```r
kir_aln2 = read.fasta('./input_data/human_kirs_v2.fas')

kir_aln2$resno = matrix(
  data = NA,
  nrow = nrow(kir_aln2$ali),
  ncol = ncol(kir_aln2$ali)
)

for (i in 1:length(kir_aln2$id)) {
  tmp = data.frame('seq' = kir_aln2$ali[i, ]) %>%
    dplyr::mutate(ifelse(seq == '-', NA, cumsum(seq != '-')))
  kir_aln2$resno[i, ] = as.numeric(tmp[, 2])
}

dbvar$kir21_resno = rep(NA, nrow(dbvar))
dbvar$kir21_resid = rep(NA, nrow(dbvar))
kcnj2_ref = which(kir_aln2$id == 'KCNJ2')
for (i in 1:nrow(dbvar)) {
  idx1 = which(kir_aln2$id == dbvar[i, 'Gene'])
  pos_idx = as.numeric(unlist(strsplit(dbvar[i, 'Mutation'], split = '[A-Z]'))[2])
  idx2 = which(kir_aln2$resno[idx1, ] == pos_idx)
  dbvar$kir21_resno[i] = kir_aln2$resno[1, idx2]
  dbvar$kir21_resid[i] = kir_aln2$ali[1, idx2]
}
dbvar[, c('KCNJx_WT_resid', 'KCNJx_MUT_resid')] = t(sapply(dbvar$Mutation, function(x)
{
  c(gsub('([A-Z])[0-9]*([A-Z])', '\\1', x),
    gsub('([A-Z])[0-9]*([A-Z])', '\\2', x))
}))
dbvar$KCNJx_eq_KCNJ2 = as.factor(ifelse(dbvar$KCNJx_WT_resid == dbvar$kir21_resid, 1, 0))
dbvar$Gene = as.factor(dbvar$Gene)
dbvar$Clinical_Significance = as.factor(dbvar$Clinical_Significance.y)

idx = match(dbvar$kir21_resno, irk$Kir2_1_Resno_mus)
dbvar$kir21_FLAG_resno = irk$Kir2_1_FLAG_Resno_mus[idx]

cv_kir21 = subset(dbvar, Gene == 'KCNJ2')
cv_kir21[, c('Clinical_Significance.x', 'Clinical_Significance.y')] = NULL
cv_kir21 = merge(data.frame('kir21_FLAG_resno' = seq(1, 400, 1)), cv_kir21, all.x = T)

plot_data_cv_kir21 = melt(
  acast(
    data = cv_kir21,
    KCNJx_MUT_resid ~ kir21_FLAG_resno,
    value.var = 'Clinical_Significance',
    fill = NA_real_,
    fun.aggregate = I
  )
)
colnames(plot_data_cv_kir21) = c("KCNJx_MUT_resid",
                                 'kir21_FLAG_resno',
                                 'Clinical_Significance')
#cv_kir21 = merge(plot_data_cv_kir21,cv_kir21, all.x = T)
```

```r
cv_kir21 = merge(plot_data_cv_kir21, cv_kir21[, c('KCNJx_MUT_resid', 'kir21_FLAG_resno',
                                                   'Source')], all.x = T)
cv_kir21$KCNJx_MUT_resid = factor(
  cv_kir21$KCNJx_MUT_resid,
  levels = c(
    'H',
    'K',
    'R',
    'D',
    'E',
    'C',
    'M',
    'N',
    'Q',
    'S',
    'T',
    'A',
    'I',
    'L',
    'V',
    'W',
    'F',
    'Y',
    'G',
    'P'
  )
)
cv_kir21$Clinical_Significance[is.na(cv_kir21$Clinical_Significance) &
                                 cv_kir21$Source == "Gnomad only"] = 5
cv_kir21$Clinical_Significance = as.factor(cv_kir21$Clinical_Significance)
cv_kir21$Clinical_Significance = as.character(plyr::mapvalues(
  cv_kir21$Clinical_Significance,
  from = c('1', '2', '3', '4', '5'),
  to = c('#4E79A7', '#F28E2B', '#E15759',
         '#76B7B2', '#59A14F')
))
cv_kir21$Clinical_Significance[is.na(cv_kir21$Clinical_Significance)] = 'white'
```

##OUTPUT: Fig1a

```r
dbvar$is.KCNJ2 = as.factor(ifelse(dbvar$Gene == 'KCNJ2', 'Kir2.1', 'other Kir'))
table(dbvar$Clinical_Significance, dbvar$is.KCNJ2) %>%
  kbl(caption = 'Fig1a') %>%
  kable_paper('hover', full_width = F, html_font = 'helvetica') %>%
  save_kable(file = paste0(fig_path, 'Fig1a.pdf'))
```

##OUTPUT: Fig2a

```r
Fig2a = ggplot(kir21_surf, aes(x = surface_score, color = is.wt)) +
  geom_density(na.rm = T, bw = 'SJ') +
  ggtitle('Figure 2a')
Fig2a = annotate_figure(Fig2a, fig.lab = 'Fig2a')
ggsave(
  paste0(fig_path, 'Fig2a.pdf'),
  plot = Fig2a,
```

```
  width = 8,
  height = 11,
  units = 'in'
)
```

##OUTPUT: Fig2b

```
Fig2b_row1 = ggplot(data = kir21_surf[kir21_surf$position %in% c(1:100), ],
                    aes(x = position, y = mutation, fill = surface_score)) +
  geom_tile(aes(color = as.factor(is.wt)), size = 0.2) +
  scale_fill_continuous_divergingx(
    palette = 'RdBu',
    mid = 0,
    l1 = 0.2,
    l3 = 0.2,
    p1 = 0.9,
    p3 = .4,
    p4 = 0.9,
    rev = TRUE,
    na.value = 'gray60'
  ) +
  scale_color_manual(values = c(NA, 'green')) +
  scale_x_continuous(breaks = seq(0, 400, by = 5)) +
  coord_fixed(ratio = 1) +
  theme(
    panel.background = element_rect(
      fill = "white",
      colour = "white",
      size = 0.5,
      linetype = "solid"
    ),
    panel.grid.major = element_line(
      size = 0.5,
      linetype = 'solid',
      colour = "lightgray"
    ),
    panel.grid.minor = element_blank(),
    axis.text.x = element_text(angle = 90, hjust = 1),
    axis.text = element_text(size = 7)
  )
Fig2b_row2 = ggplot(data = kir21_surf[kir21_surf$position %in% c(101:200), ],
                    aes(x = position, y = mutation, fill = surface_score)) +
  geom_tile(aes(color = as.factor(is.wt)), size = 0.2) +
  scale_fill_continuous_divergingx(
    palette = 'RdBu',
    mid = 0,
    l1 = 0.2,
    l3 = 0.2,
    p1 = 0.9,
    p3 = .4,
    p4 = 0.9,
    rev = TRUE,
    na.value = 'gray60'
  ) +
```

```
  scale_color_manual(values = c(NA, 'green')) +
  scale_x_continuous(breaks = seq(0, 400, by = 5)) +
  coord_fixed(ratio = 1) +
  theme(
    panel.background = element_rect(
      fill = "white",
      colour = "white",
      size = 0.5,
      linetype = "solid"
    ),
    panel.grid.major = element_line(
      size = 0.5,
      linetype = 'solid',
      colour = "lightgray"
    ),
    panel.grid.minor = element_blank(),
    axis.text.x = element_text(angle = 90, hjust = 1),
    axis.text = element_text(size = 7)
  )
Fig2b_row3 = ggplot(data = kir21_surf[kir21_surf$position %in% c(201:300), ],
                    aes(x = position, y = mutation, fill = surface_score)) +
  geom_tile(aes(color = as.factor(is.wt)), size = 0.2) +
  scale_fill_continuous_divergingx(
    palette = 'RdBu',
    mid = 0,
    l1 = 0.2,
    l3 = 0.2,
    p1 = 0.9,
    p3 = .4,
    p4 = 0.9,
    rev = TRUE,
    na.value = 'gray60'
  ) +
  scale_color_manual(values = c(NA, 'green')) +
  scale_x_continuous(breaks = seq(0, 400, by = 5)) +
  coord_fixed(ratio = 1) +
  theme(
    panel.background = element_rect(
      fill = "white",
      colour = "white",
      size = 0.5,
      linetype = "solid"
    ),
    panel.grid.major = element_line(
      size = 0.5,
      linetype = 'solid',
      colour = "lightgray"
    ),
    panel.grid.minor = element_blank(),
    axis.text.x = element_text(angle = 90, hjust = 1),
    axis.text = element_text(size = 7)
  )
Fig2b_row4 = ggplot(data = kir21_surf[kir21_surf$position %in% c(301:400), ],
```

```
                    aes(x = position, y = mutation, fill = surface_score)) +
  geom_tile(aes(color = as.factor(is.wt)), size = 0.2) +
  scale_fill_continuous_divergingx(
    palette = 'RdBu',
    mid = 0,
    l1 = 0.2,
    l3 = 0.2,
    p1 = 0.9,
    p3 = .4,
    p4 = 0.9,
    rev = TRUE,
    na.value = 'gray60'
  ) +
  scale_color_manual(values = c(NA, 'green')) +
  scale_x_continuous(breaks = seq(0, 400, by = 5)) +
  coord_fixed(ratio = 1) +
  theme(
    panel.background = element_rect(
      fill = "white",
      colour = "white",
      size = 0.5,
      linetype = "solid"
    ),
    panel.grid.major = element_line(
      size = 0.5,
      linetype = 'solid',
      colour = "lightgray"
    ),
    panel.grid.minor = element_blank(),
    axis.text.x = element_text(angle = 90, hjust = 1),
    axis.text = element_text(size = 7)
  )

Fig2b = ggarrange(Fig2b_row1,
                  Fig2b_row2,
                  Fig2b_row3,
                  Fig2b_row4,
                  nrow = 4,
                  ncol = 1)
Fig2b = annotate_figure(Fig2b, fig.lab = 'Fig2b')
ggsave(
  paste0(fig_path, 'Fig2b.pdf'),
  plot = Fig2b,
  width = 8,
  height = 11,
  units = 'in'
)
```

##OUTPUT: Fig2g

```
act_set = c(
  242,
  244,
  245,
```

```
  246,
  247,
  248,
  250,
  252,
  269,
  333,
  335,
  336,
  338,
  340,
  341,
  342,
  345,
  356,
  357,
  358
)
ctd_set = c(190:400)[c(190:400) %!in% act_set]

kir21_surf$wt_resid = as.factor(kir21_surf$wt_resid)

kir21_surf$fmut = plyr::mapvalues(
  kir21_surf$mutation,
  from = c(
    'A',
    'C',
    'D',
    'E',
    'F',
    'G',
    'H',
    'I',
    'K',
    'L',
    'M',
    'N',
    'P',
    'Q',
    'R',
    'S',
    'T',
    'V',
    'W',
    'Y'
  ),
  to = c(
    'NP',
    'P',
    'C',
    'C',
    'A',
    'NP',
```

```
    'C',
    'NP',
    'C',
    'NP',
    'NP',
    'P',
    'NP',
    'P',
    'C',
    'P',
    'P',
    'NP',
    'A',
    'A'
  )
)
kir21_surf$fwt = plyr::mapvalues(
  kir21_surf$wt_resid,
  from = c(
    'A',
    'C',
    'D',
    'E',
    'F',
    'G',
    'H',
    'I',
    'K',
    'L',
    'M',
    'N',
    'P',
    'Q',
    'R',
    'S',
    'T',
    'V',
    'W',
    'Y'
  ),
  to = c(
    'NP',
    'P',
    'C',
    'C',
    'A',
    'NP',
    'C',
    'NP',
    'C',
    'NP',
    'NP',
    'P',
```

```
    'NP',
    'P',
    'C',
    'P',
    'P',
    'NP',
    'A',
    'A'
  )
)

kir21_surf$Int = interaction(kir21_surf$fwt, kir21_surf$fmut)
kir21_surf$Int2 = interaction(kir21_surf$wt_resid, kir21_surf$fmut)
kir21_surf$Int3 = interaction(kir21_surf$wt_resid, kir21_surf$mutation)
kir21_surf$Int4 = ifelse(kir21_surf$fwt == kir21_surf$fmut, 1, 0)

Fig2g = ggplot(kir21_surf[kir21_surf$position %in% act_set, ],
               aes(
                 x = reorder(Int4, surface_score, FUN = median, na.rm = TRUE),
                 y = surface_score
               )) +
  geom_boxplot() +
  geom_jitter(width = 0.1,
              size = 0.5,
              alpha = 0.2) +
  ylim(-6, 4) +
  ggpubr::stat_compare_means(
    aes(label = ..p.format..),
    method = 't.test',
    paired = F,
    method.args = list(alternative = 'less')
  ) +
  facet_wrap(fwt ~ .) +
  ggtitle('Fig2g')
ggsave(
  Fig2g,
  filename = paste0(fig_path, 'Fig2g.pdf'),
  width = 8,
  height = 11,
  units = 'in'
)
```

```
## Warning: Removed 38 rows containing non-finite values (stat_boxplot).

## Warning: Removed 38 rows containing non-finite values (stat_compare_means).

## Warning: Removed 38 rows containing missing values (geom_point).
```

##FOLD STABILITY vs GATING

```
#Regions involved in gating transitions to do not contribute to fold stability####
surf_means = kir21_surf %>%
  group_by(position) %>%
  summarize(mean_surf = mean(surface_score, na.rm = T))
idx = match(surf_means$position, irk$Kir2_1_FLAG_Resno_mus)
surf_means$allosteric = irk$allosteric[idx]
```

```r
surf_means$tol_pos = ifelse(surf_means$mean_surf > -0.2, 1, 0)
surf_means$gat_pos = ifelse(!is.na(surf_means$allosteric), 1, 0)

fisher.test(surf_means$tol_pos, surf_means$gat_pos)
```

```
##
##  Fisher's Exact Test for Count Data
##
## data:  surf_means$tol_pos and surf_means$gat_pos
## p-value = 8.091e-05
## alternative hypothesis: true odds ratio is not equal to 1
## 95 percent confidence interval:
##  1.540354 3.981764
## sample estimates:
## odds ratio
##   2.466179
```

```r
expss::cro(surf_means$tol_pos, surf_means$gat_pos)
```

	surf_means$gat_pos	
	0	1
surf_means$tol_pos		
0	171	43
1	111	69
#Total cases	282	112

##SELECTIVITY FILTER

```r
df = merge(kir21_func, kir21_surf)
df$diff = df$surface_score - df$function_score
idx = df$function_SE > 1 | df$surface_SE > 1
idx[is.na(idx)] = TRUE
df[idx, c('function_score', 'surface_score')] = NA

surf_func_means = df %>%
  group_by(position) %>%
  summarize(
    mean_surf = mean(surface_score, na.rm = T),
    mean_func = mean(function_score, na.rm = T),
    median_surf = median(surface_score, na.rm = T),
    median_func = median(function_score, na.rm = T),
    var_surf = var(surface_score, na.rm = T),
```

```
    var_func = var(function_score, na.rm = T)
  )

df_sel = df[df$position %in% c(140:162), ]
df_sel$filt = ifelse(df_sel$position %in% c(149:155), 1, 0)

df_sel$w = 1 / (df_sel$surface_SE / sum(df_sel$surface_SE, na.rm = T))

df_sel$fmut = plyr::mapvalues(
  df_sel$mutation,
  from = c(
    'A',
    'C',
    'D',
    'E',
    'F',
    'G',
    'H',
    'I',
    'K',
    'L',
    'M',
    'N',
    'P',
    'Q',
    'R',
    'S',
    'T',
    'V',
    'W',
    'Y'
  ),
  to = c(
    'NP',
    'P',
    'C',
    'C',
    'A',
    'NP',
    'C',
    'NP',
    'C',
    'NP',
    'NP',
    'P',
    'NP',
    'P',
    'C',
    'P',
    'P',
    'NP',
    'A',
    'A'
```

```
  )
)

df_sel_plot  = df_sel %>%
  group_by(position, fmut) %>%
  summarise(
    w_mean = weighted.mean(surface_score, w, na.rm = T),
    mean = median(surface_score, na.rm = T),
    sd = sd(surface_score, na.rm = T),
    n = n()
  ) %>%
  dplyr::mutate(
    se = sd / sqrt(n),
    lower.ci = w_mean - qt(1 - (0.1 / 2), n - 1) * se,
    upper.ci = w_mean + qt(1 - (0.1 / 2), n - 1) * se
  )
```

```
## `summarise()` has grouped output by 'position'. You can override using the
## `.groups` argument.
```

##OUTPUT: Fig3c

```
Fig3c = ggplot() +
  geom_point(
    data = df_sel,
    aes(x = position, y = surface_score, color = as.factor(filt)),
    alpha = 0.3
  ) +
  geom_line(data = df_sel_plot, aes(x = position, y = w_mean)) +
  geom_hline(yintercept = surf_median_WT) +
  geom_hline(yintercept = surf_median_WT + 3 * surf_SD_WT, linetype = 'dashed') +
  geom_hline(yintercept = surf_median_WT - 3 * surf_SD_WT, linetype = 'dashed') +
  geom_ribbon(
    data = df_sel_plot,
    aes(
      x = position,
      y = w_mean,
      ymin = lower.ci,
      ymax = upper.ci
    ),
    fill = 'grey90',
    color = "grey70",
    alpha = 0.4
  ) +
  scale_x_continuous(breaks = seq(140, 162, 1)) +
  facet_wrap(fmut ~ .) +
  theme_classic()
ggsave(
  paste0(fig_path, 'Fig3c.pdf'),
  plot = Fig3c,
  width = 8,
  height = 11,
  units = 'in'
)
```

```
## Warning: Removed 78 rows containing missing values (geom_point).
```

##OUTPUT: Fig3d

```
Fig3d = ggplot(df_sel, aes(x = surface_score, color = as.factor(filt))) +
  stat_ecdf() +
  theme_classic()
ggsave(
  paste0(fig_path, 'Fig3d.pdf'),
  plot = Fig3d,
  width = 8,
  height = 11,
  units = 'in'
)
```

```
## Warning: Removed 78 rows containing non-finite values (stat_ecdf).
```

```
ks.test(df_sel[df_sel$filt == 1, 'surface_score'], df_sel[df_sel$filt == 0,
                                                         'surface_score'])
```

```
##
##  Two-sample Kolmogorov-Smirnov test
##
## data:  df_sel[df_sel$filt == 1, "surface_score"] and df_sel[df_sel$filt == 0, "surface_score"]
## D = 0.45511, p-value = 1.918e-13
## alternative hypothesis: two-sided
```

##OUTPUT: Fig4a

```
Fig4a = ggplot(kir21_func, aes(x = function_score, color = is.wt)) +
  geom_density(na.rm = T, bw = 'SJ') +
  ggtitle('Figure 4a')
ggsave(
  paste0(fig_path, 'Fig4a.pdf'),
  plot = Fig4a,
  width = 8,
  height = 11,
  units = 'in'
)
```

##OUTPUT: Fig4c

```
Fig4c_row1 = ggplot(data = kir21_func[kir21_func$position %in% c(1:100), ],
                    aes(x = position, y = mutation, fill = function_score)) +
  geom_tile(aes(color = as.factor(is.wt)), size = 0.2) +
  scale_fill_continuous_divergingx(
    palette = 'PrGn',
    mid = 0,
    l1 = 0,
    l3 = 0.3,
    p1 = 0.4,
    p3 = .6,
    p4 = 0.9,
    rev = TRUE,
    na.value = 'gray60'
  ) +
  scale_color_manual(values = c(NA, 'red')) +
  scale_x_continuous(breaks = seq(0, 400, by = 5)) +
```

```
  coord_fixed(ratio = 1) +
  theme(
    panel.background = element_rect(
      fill = "white",
      colour = "white",
      size = 0.5,
      linetype = "solid"
    ),
    panel.grid.major = element_line(
      size = 0.5,
      linetype = 'solid',
      colour = "lightgray"
    ),
    panel.grid.minor = element_blank(),
    axis.text.x = element_text(angle = 90, hjust = 1),
    axis.text = element_text(size = 7)
  )
Fig4c_row2 = ggplot(data = kir21_func[kir21_func$position %in% c(101:200), ],
                    aes(x = position, y = mutation, fill = function_score)) +
  geom_tile(aes(color = as.factor(is.wt)), size = 0.2) +
  scale_fill_continuous_divergingx(
    palette = 'PrGn',
    mid = 0,
    l1 = 0,
    l3 = 0.3,
    p1 = 0.4,
    p3 = .6,
    p4 = 0.9,
    rev = TRUE,
    na.value = 'gray60'
  ) +
  scale_color_manual(values = c(NA, 'red')) +
  scale_x_continuous(breaks = seq(0, 400, by = 5)) +
  coord_fixed(ratio = 1) +
  theme(
    panel.background = element_rect(
      fill = "white",
      colour = "white",
      size = 0.5,
      linetype = "solid"
    ),
    panel.grid.major = element_line(
      size = 0.5,
      linetype = 'solid',
      colour = "lightgray"
    ),
    panel.grid.minor = element_blank(),
    axis.text.x = element_text(angle = 90, hjust = 1),
    axis.text = element_text(size = 7)
  )
Fig4c_row3 = ggplot(data = kir21_func[kir21_func$position %in% c(201:300), ],
                    aes(x = position, y = mutation, fill = function_score)) +
  geom_tile(aes(color = as.factor(is.wt)), size = 0.2) +
```

```
  scale_fill_continuous_divergingx(
    palette = 'PrGn',
    mid = 0,
    l1 = 0,
    l3 = 0.3,
    p1 = 0.4,
    p3 = .6,
    p4 = 0.9,
    rev = TRUE,
    na.value = 'gray60'
  ) +
  scale_color_manual(values = c(NA, 'red')) +
  scale_x_continuous(breaks = seq(0, 400, by = 5)) +
  coord_fixed(ratio = 1) +
  theme(
    panel.background = element_rect(
      fill = "white",
      colour = "white",
      size = 0.5,
      linetype = "solid"
    ),
    panel.grid.major = element_line(
      size = 0.5,
      linetype = 'solid',
      colour = "lightgray"
    ),
    panel.grid.minor = element_blank(),
    axis.text.x = element_text(angle = 90, hjust = 1),
    axis.text = element_text(size = 7)
  )
Fig4c_row4 = ggplot(data = kir21_func[kir21_func$position %in% c(301:400), ],
                    aes(x = position, y = mutation, fill = function_score)) +
  geom_tile(aes(color = as.factor(is.wt)), size = 0.2) +
  scale_fill_continuous_divergingx(
    palette = 'PrGn',
    mid = 0,
    l1 = 0,
    l3 = 0.3,
    p1 = 0.4,
    p3 = .6,
    p4 = 0.9,
    rev = TRUE,
    na.value = 'gray60'
  ) +
  scale_color_manual(values = c(NA, 'red')) +
  scale_x_continuous(breaks = seq(0, 400, by = 5)) +
  coord_fixed(ratio = 1) +
  theme(
    panel.background = element_rect(
      fill = "white",
      colour = "white",
      size = 0.5,
      linetype = "solid"
```

```
    ),
    panel.grid.major = element_line(
      size = 0.5,
      linetype = 'solid',
      colour = "lightgray"
    ),
    panel.grid.minor = element_blank(),
    axis.text.x = element_text(angle = 90, hjust = 1),
    axis.text = element_text(size = 7)
  )

Fig4c = ggarrange(Fig4c_row1,
                  Fig4c_row2,
                  Fig4c_row3,
                  Fig4c_row4,
                  nrow = 4,
                  ncol = 1)
Fig4c = annotate_figure(Fig4c, fig.lab = 'Fig4c')
ggsave(
  paste0(fig_path, 'Fig4c.pdf'),
  plot = Fig4c,
  width = 8,
  height = 11,
  units = 'in'
)
```

##OUTPUT: Fig4d-g

```
sel_set1 = c(382:388, 2:31, 377:381)

pos_fig4_surf1 = acast(
  data = kir21_surf[kir21_surf$position %in% sel_set1, ],
  mutation ~ position,
  value.var = 'surface_score',
  fill = NA_real_,
  fun.aggregate = I
)
pos_fig4_func1 = acast(
  data = kir21_func[kir21_func$position %in% sel_set1, ],
  mutation ~ position,
  value.var = 'function_score',
  fill = NA_real_,
  fun.aggregate = I
)

tmpplot = gplots::heatmap.2(
  pos_fig4_surf1,
  Rowv = T,
  Colv = T,
  scale = 'none',
  dendrogram = 'none',
  trace = 'none',
  symkey = F,
  symbreaks = T,
```

```
  col = scico::scico(20, palette = 'vik', direction = -1),
  na.color = NA,
  ylab = 'Position',
  xlab = 'Mutation',
  #main = 'all',
  distfun = function(x)
    dist(x, method = "maximum"),
  hclustfun = function(x)
    hclust(x, method = "complete"),
  reorderfun = function(d, w)
    reorder(d, w, agglo.FUN = mean)
)
```

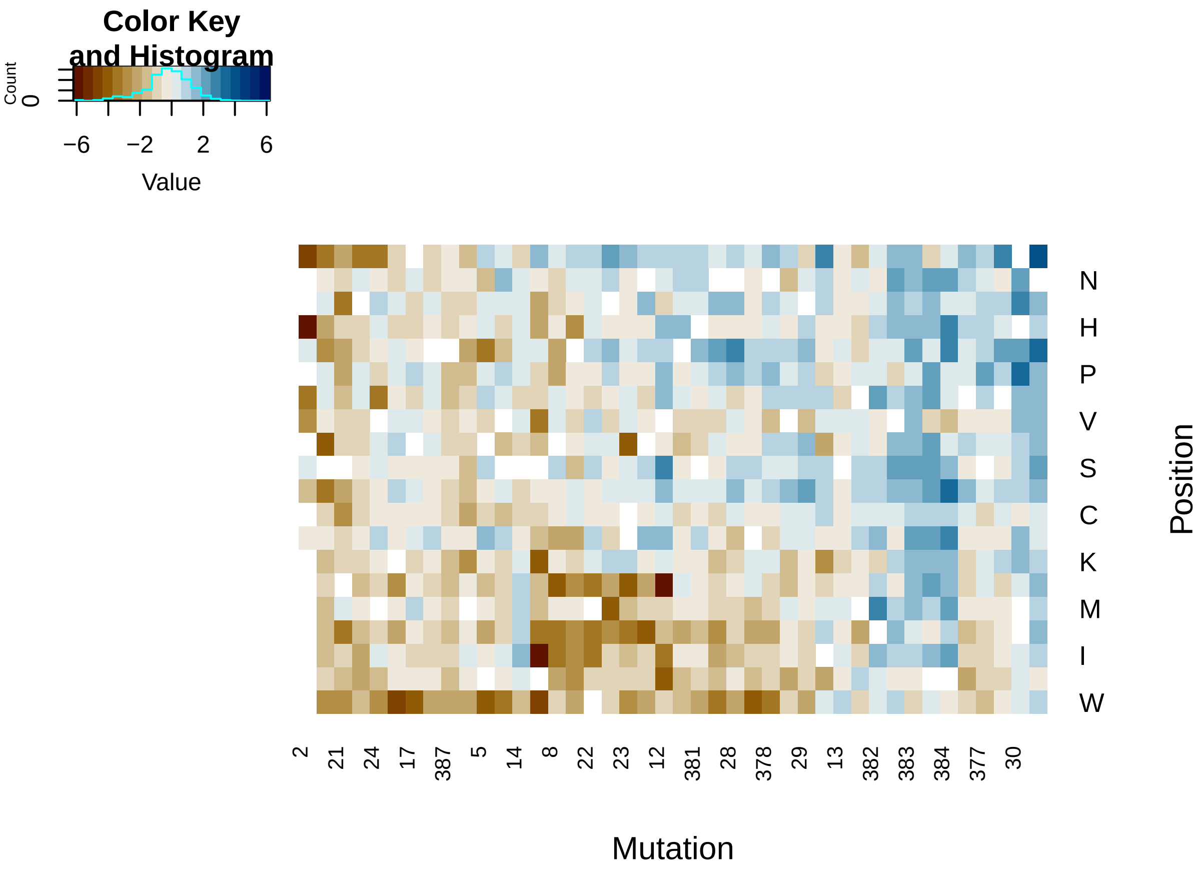


```
fig4_surf1_rowInd = tmpplot$rowInd
fig4_surf1_colInd = tmpplot$colInd

col_fun1 = circlize::colorRamp2(c(-6, -4, -2, 0, 2, 4, 6),
                                divergingx_hcl(7, palette = 'RdBu'))
col_fun2 = circlize::colorRamp2(c(-3, -2, -1, 0, 1, 2, 3),
                                divergingx_hcl(7, palette = 'PrGn'))


fig4_surf1 = ggplotify::as.ggplot(
  ComplexHeatmap::Heatmap(
    pos_fig4_surf1[rev(fig4_surf1_rowInd), fig4_surf1_colInd],
    na_col = 'gray60',
```

```
    cluster_rows = F,
    cluster_columns = F,
    col = col_fun1,
    heatmap_legend_param = list(at = c(-6, 0, 6))
  )
)


fig4_func1 = ggplotify::as.ggplot(
  ComplexHeatmap::Heatmap(
    pos_fig4_func1[rev(fig4_surf1_rowInd), fig4_surf1_colInd],
    na_col = 'gray60',
    cluster_rows = F,
    cluster_columns = F,
    col = col_fun2,
    heatmap_legend_param = list(at = c(-3, 0, 3))
  )
)


sel_set2 = c(80:82, 190:198, 225:235, 303:320)

pos_fig4_surf2 = acast(
  data = kir21_surf[kir21_surf$position %in% sel_set2 &
                      is.na(kir21_surf$trust), ],
  mutation ~ position,
  value.var = 'surface_score',
  fill = NA_real_,
  fun.aggregate = I
)
pos_fig4_func2 = acast(
  data = kir21_func[kir21_func$position %in% sel_set2 &
                      is.na(kir21_func$trust), ],
  mutation ~ position,
  value.var = 'function_score',
  fill = NA_real_,
  fun.aggregate = I
)

tmpplot = gplots::heatmap.2(
  pos_fig4_func2,
  Rowv = T,
  Colv = T,
  scale = 'none',
  dendrogram = 'none',
  trace = 'none',
  symkey = F,
  symbreaks = T,
  col = scico::scico(20, palette = 'vik', direction = -1),
  na.color = NA,
  ylab = 'Position',
  xlab = 'Mutation',
  #main = 'all',
```

```
  distfun = function(x)
    dist(x, method = "maximum"),
  hclustfun = function(x)
    hclust(x, method = "ward.D2"),
  reorderfun = function(d, w)
    reorder(d, w, agglo.FUN = mean)
)
```

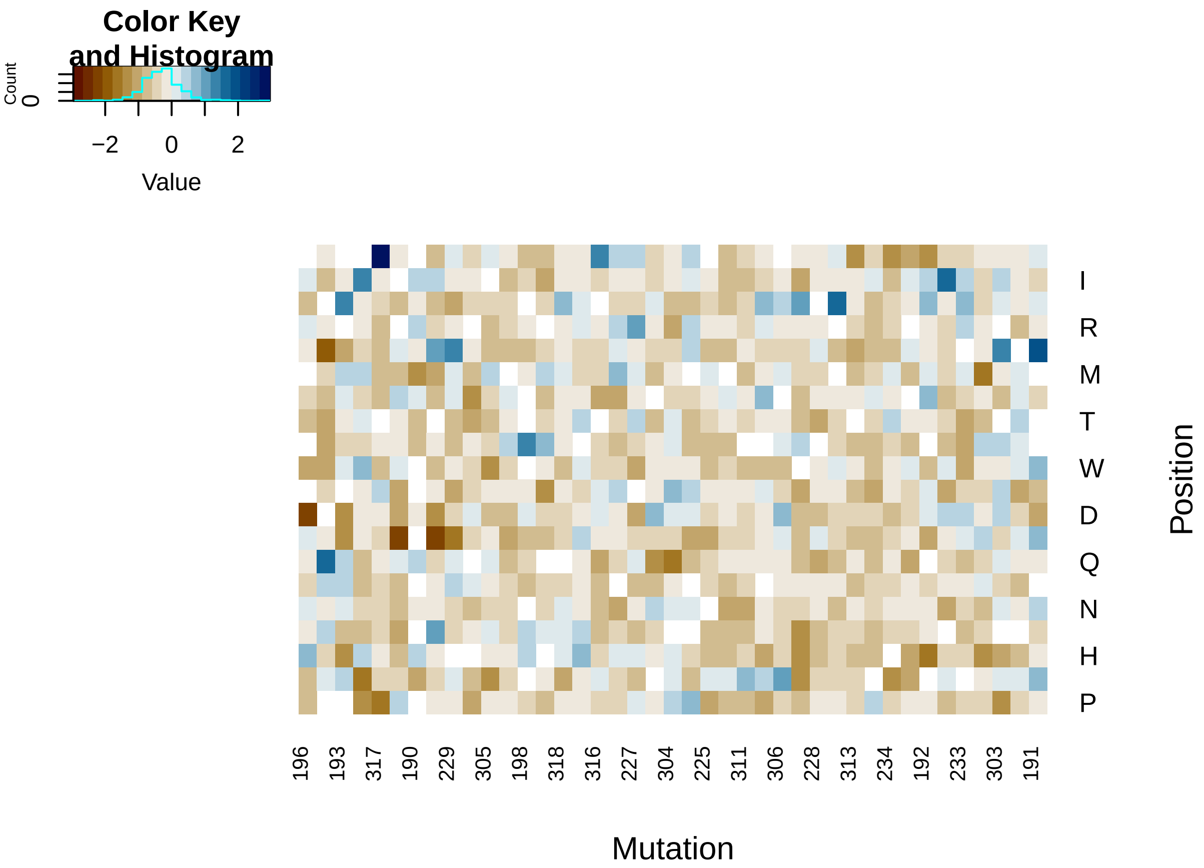


```
fig4_func2_rowInd = tmpplot$rowInd
fig4_func2_colInd = tmpplot$colInd

fig4_surf2 = ggplotify::as.ggplot(
  ComplexHeatmap::Heatmap(
    pos_fig4_surf2[rev(fig4_func2_rowInd), fig4_func2_colInd],
    na_col = 'gray60',
    cluster_rows = F,
    cluster_columns = F,
    col = col_fun1,
    heatmap_legend_param = list(at = c(-6, 0, 6))
  )
)

fig4_func2 = ggplotify::as.ggplot(
  ComplexHeatmap::Heatmap(
    pos_fig4_func2[rev(fig4_func2_rowInd), fig4_func2_colInd],
    na_col = 'gray60',
```

```
    cluster_rows = F,
    cluster_columns = F,
    col = col_fun2,
    heatmap_legend_param = list(at = c(-3, 0, 3))
  )
)

Fig4d_g = ggarrange(
  fig4_surf1,
  fig4_surf2,
  fig4_func1,
  fig4_func2,
  ncol = 2,
  nrow = 2,
  labels = c('d', 'f', 'e', 'g')
)
Fig4d_g = annotate_figure(Fig4d_g, fig.lab = 'Fig4d-g')
Fig4d_g
```

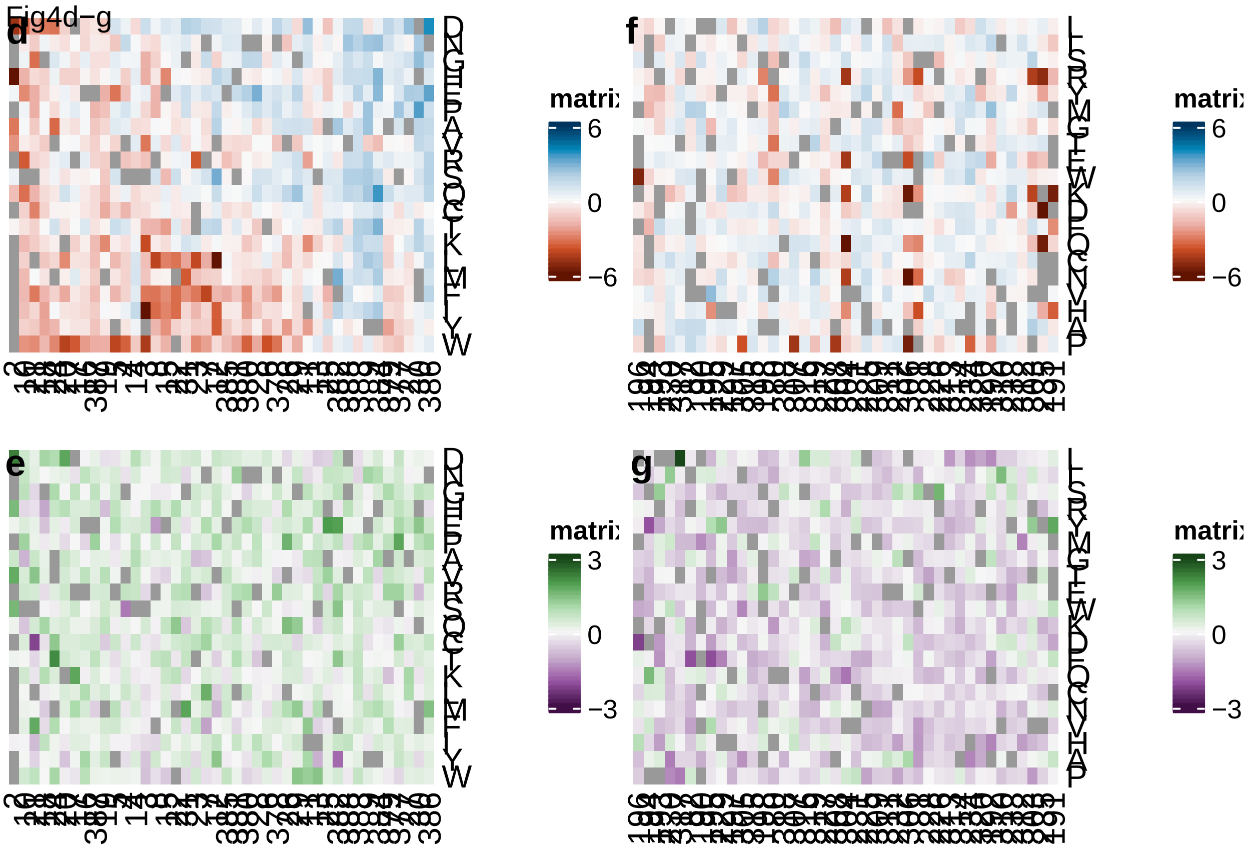


```
ggsave(
  paste0(fig_path, 'Fig4d_g.pdf'),
  plot = Fig4d_g,
  width = 8,
  height = 11,
  units = 'in'
)
```

##OUTPUT: Fig5c

```
surf_func_means_plot = data.frame(
  position = rep(surf_func_means$position, 2),
  score_type = c(rep(
    "surf", length(surf_func_means$position)
  ), rep(
    "func", length(surf_func_means$position)
  )),
  pairing = rep(1:length(surf_func_means$position), 2),
  diff_sign = rep(interaction(
    sign(surf_func_means$mean_surf - surf_median_WT),
    sign(surf_func_means$mean_func - func_median_WT)
  ), 2),
  score = c(surf_func_means$mean_surf, surf_func_means$mean_func)
)


Fig5c = ggplot(surf_func_means_plot, aes(x = position, y = score)) +
  geom_line(aes(group = pairing, color = diff_sign)) +
  geom_point(aes(color = score_type)) +
  geom_hline(yintercept = func_median_WT, color = '#76B7B2') +
  geom_hline(
    yintercept = func_median_WT + func_SD_WT,
    linetype = 'dashed',
    color = '#76B7B2'
  ) +
  geom_hline(
    yintercept = func_median_WT - func_SD_WT,
    linetype = 'dashed',
    color = '#76B7B2'
  ) +
  geom_hline(yintercept = surf_median_WT, color = '#EDC948') +
  geom_hline(
    yintercept = surf_median_WT + surf_SD_WT,
    linetype = 'dashed',
    color = '#EDC948'
  ) +
  geom_hline(
    yintercept = surf_median_WT - surf_SD_WT,
    linetype = 'dashed',
    color = '#EDC948'
  ) +
  scale_color_manual(values = c(
    '#4E79A7',
    '#F28E2B',
    '#E15759',
    '#59A14F',
    '#76B7B2',
    '#EDC948',
    '#BAB0AC'
  )) +
  theme_classic2() +
  scale_x_continuous(breaks = seq(0, 400, 10)) +
```

```r
  #theme(legend.position = "none") +
  ggtitle('Fig5c')
ggsave(
  paste0(fig_path, 'Fig5c.pdf'),
  plot = Fig5c,
  width = 8,
  height = 11,
  units = 'in'
)
```

##Comparison to Literature

```r
#Ma & Welling
welling_comp = openxlsx::read.xlsx('./input_data/welling_comparison.xlsx')
welling_comp = merge(welling_comp, df)

#Minor & Jan
minor_comp = openxlsx::read.xlsx('./input_data/minor_comparison.xlsx')
df_trim = df[df$position %in% unique(minor_comp$position), ]
minor_comp = merge(
  minor_comp,
  df_trim,
  by = c("position", 'mutation', 'wt_resid'),
  all.y = TRUE
)
minor_comp$is.present = ifelse(is.na(minor_comp$minor_count), 0, 1)

#Dart & Sutcliffe####
sutcliffe_comp = openxlsx::read.xlsx('./input_data/sutcliffe_comp.xlsx')
sutcliffe_comp = merge(sutcliffe_comp, df)


ma_comp_a = ggplot(welling_comp, aes(x = welling_surface_score, y = surface_score)) +
  geom_smooth(method = 'lm') +
  ggrepel::geom_label_repel(
    aes(label = label),
    box.padding   = 1.5,
    point.padding = 1.7,
    segment.color = 'grey50'
  ) +
  geom_point() +
  geom_errorbar(aes(ymin = surface_score - surface_SE,
                    ymax = surface_score + surface_SE)) +
  ggpubr::stat_cor(method = 'pearson') +
  theme_classic()

minor_comp_a = ggplot(minor_comp, aes(x = surface_score,
                                      color = as.factor(is.present))) +
  stat_ecdf() + theme_classic()
minor_comp_b = ggplot(minor_comp, aes(x = function_score,
                                      color = as.factor(is.present))) +
  stat_ecdf() + theme_classic()

ks.test(minor_comp$surface_score[minor_comp$is.present == 1],
```

```
       minor_comp$surface_score[minor_comp$is.present == 0])
```

```
##
##  Two-sample Kolmogorov-Smirnov test
##
## data:  minor_comp$surface_score[minor_comp$is.present == 1] and minor_comp$surface_score[minor_comp$i
## D = 0.38638, p-value = 7.65e-11
## alternative hypothesis: two-sided
```

```
ks.test(minor_comp$function_score[minor_comp$is.present == 1],
        minor_comp$function_score[minor_comp$is.present == 0])
```

```
##
##  Two-sample Kolmogorov-Smirnov test
##
## data:  minor_comp$function_score[minor_comp$is.present == 1] and minor_comp$function_score[minor_comp
## D = 0.31094, p-value = 3.584e-07
## alternative hypothesis: two-sided
```

```
dart_comp_a = ggplot(sutcliffe_comp, aes(x = ag_block, y = surface_score)) +
  geom_smooth(method = 'lm') +
  ggrepel::geom_label_repel(
    aes(label = label),
    box.padding   = 0.35,
    point.padding = 0.5,
    segment.color = 'grey50'
  ) +
  geom_point() +
  geom_errorbar(aes(ymin = surface_score - surface_SE,
                    ymax = surface_score + surface_SE)) +
  ggpubr::stat_cor(method = 'pearson') +
  theme_classic()

dart_comp_b = ggplot(sutcliffe_comp,
                     aes(x = ag_block, y = function_score / surface_score)) +
  geom_smooth(method = 'lm') +
  ggrepel::geom_label_repel(
    aes(label = label),
    box.padding   = 0.35,
    point.padding = 0.5,
    segment.color = 'grey50'
  ) +
  geom_point() +
  geom_errorbar(aes(
    ymin = (function_score / surface_score) - function_SE,
    ymax = (function_score / surface_score) + function_SE
  )) +
  ggpubr::stat_cor(method = 'pearson') +
  theme_classic()
```

##OUTPUT: Fig6

```
lit_comp = ggarrange(
  ma_comp_a,
  minor_comp_a,
  dart_comp_a,
```

```
  minor_comp_b,
  dart_comp_b,
  ncol = 2,
  nrow = 3
)
```

```
## `geom_smooth()` using formula 'y ~ x'

## Warning: Removed 108 rows containing non-finite values (stat_ecdf).

## `geom_smooth()` using formula 'y ~ x'

## Warning: Removed 1 rows containing non-finite values (stat_smooth).

## Warning: Removed 1 rows containing non-finite values (stat_cor).

## Warning: Removed 1 rows containing missing values (geom_label_repel).

## Warning: Removed 1 rows containing missing values (geom_point).

## Warning: Removed 108 rows containing non-finite values (stat_ecdf).

## `geom_smooth()` using formula 'y ~ x'

## Warning: Removed 1 rows containing non-finite values (stat_smooth).

## Warning: Removed 1 rows containing non-finite values (stat_cor).

## Warning: Removed 1 rows containing missing values (geom_label_repel).

## Warning: Removed 1 rows containing missing values (geom_point).
```

```
ggsave(
  paste0(fig_path, 'Fig6.pdf'),
  plot = lit_comp,
  width = 8,
  height = 11,
  units = 'in'
)
```

```
## Warning: ggrepel: 3 unlabeled data points (too many overlaps). Consider
## increasing max.overlaps
```

##OUTPUT: Fig7a-b

```
cv_kir21_df = left_join(df,
                        dbvar[dbvar$Gene == 'KCNJ2',],
                        by = c('position' = 'kir21_FLAG_resno',
                               'mutation' = 'KCNJx_MUT_resid'))

surf_cv_kir21 = ggplot(cv_kir21_df, aes(x = surface_score, color = KCNJx_eq_KCNJ2)) +
  stat_ecdf()
func_cv_kir21 = ggplot(cv_kir21_df, aes(x = function_score, color = KCNJx_eq_KCNJ2)) +
  stat_ecdf()
Fig7ab = gridExtra::grid.arrange(surf_cv_kir21, func_cv_kir21, nrow = 2)
```

```
## Warning: Removed 985 rows containing non-finite values (stat_ecdf).
## Removed 985 rows containing non-finite values (stat_ecdf).
```

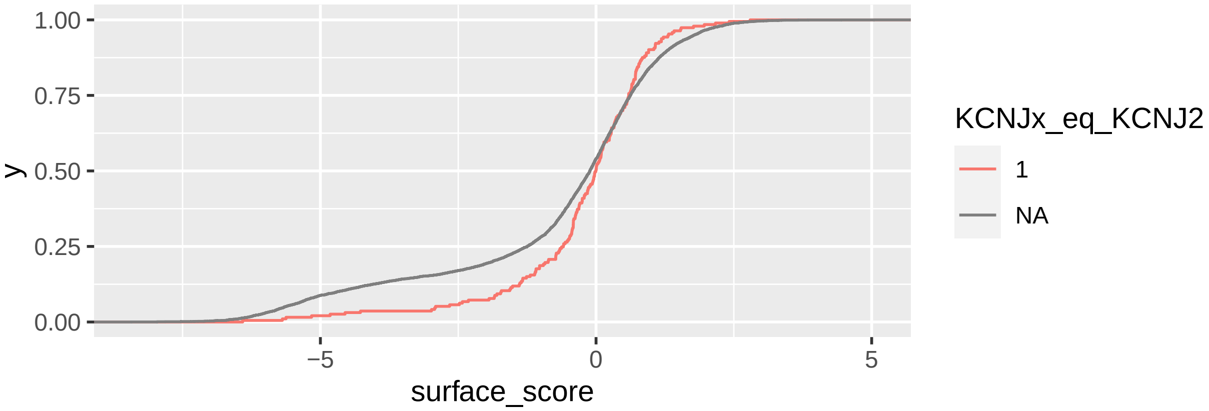

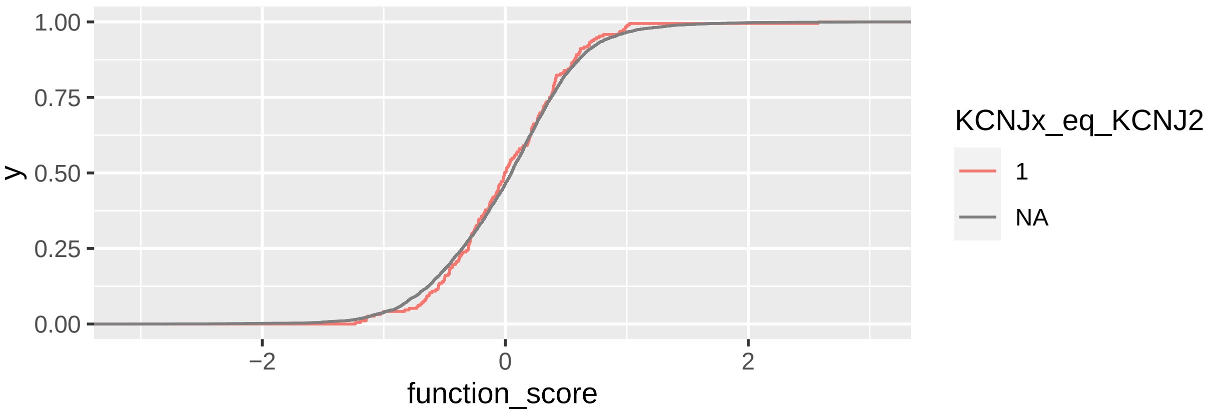


```
Fig7ab = annotate_figure(Fig7ab, fig.lab = 'Figure 7a-b')
ggsave(
  paste0(fig_path, 'Fig7ab.pdf'),
  plot = Fig7ab,
  width = 8,
  height = 11,
  units = 'in'
)
```

##VARIANT REPRESENTATION IN ClinVar and gnomAD

```
ks.test(cv_kir21_df[is.na(cv_kir21_df$KCNJx_eq_KCNJ2), 'surface_score'],
        cv_kir21_df[!is.na(cv_kir21_df$KCNJx_eq_KCNJ2), 'surface_score'])
```

```
##
##  Two-sample Kolmogorov-Smirnov test
##
## data:  cv_kir21_df[is.na(cv_kir21_df$KCNJx_eq_KCNJ2), "surface_score"] and cv_kir21_df[!is.na(cv_kir
## D = 0.12538, p-value = 0.005563
## alternative hypothesis: two-sided
```

```
ks.test(cv_kir21_df[is.na(cv_kir21_df$KCNJx_eq_KCNJ2), 'function_score'],
        cv_kir21_df[!is.na(cv_kir21_df$KCNJx_eq_KCNJ2), 'function_score'])
```

```
## Warning in ks.test(cv_kir21_df[is.na(cv_kir21_df$KCNJx_eq_KCNJ2),
## "function_score"], : p-value will be approximate in the presence of ties
```

```
##
##  Two-sample Kolmogorov-Smirnov test
```

```
##
## data:  cv_kir21_df[is.na(cv_kir21_df$KCNJx_eq_KCNJ2), "function_score"] and cv_kir21_df[!is.na(cv_ki
## D = 0.050348, p-value = 0.7298
## alternative hypothesis: two-sided
```

##clinvar assignment by score via ecdf

```
cv_df = left_join(df,
                  dbvar,
                  by = c('position' = 'kir21_FLAG_resno',
                         'mutation' = 'KCNJx_MUT_resid'))

benign_fq = quantile(cv_df[cv_df$Clinical_Significance == 'Benign', 'function_score'],
                     na.rm = T,
                     probs = c(0.1, 0.9))
patho_fq = quantile(cv_df[cv_df$Clinical_Significance == 'Pathogenic', 'function_score'],
                    na.rm = T,
                    probs = c(0.1, 0.9))
benign_sq = quantile(cv_df[cv_df$Clinical_Significance == 'Benign', 'surface_score'],
                     na.rm = T,
                     probs = c(0.1, 0.9))
patho_sq = quantile(cv_df[cv_df$Clinical_Significance == 'Pathogenic', 'surface_score'],
                    na.rm = T,
                    probs = c(0.1, 0.9))

dbvar_func = ggplot(cv_df[(cv_df$KCNJx_eq_KCNJ2 == 1 |
                             is.na(cv_df$KCNJx_eq_KCNJ2)) &
                            !is.na(cv_df$Clinical_Significance), ],
                    aes(x = function_score, color = Clinical_Significance)) +
  stat_ecdf() +
  geom_vline(xintercept = benign_fq,
             color = "#4E79A7",
             linetype = 'dashed') +
  geom_vline(xintercept = patho_fq,
             color = "#E15759",
             linetype = 'dashed') +
  ggthemes::scale_color_tableau() +
  ggtitle('cv_df function by clinvar assignment')

dbvar_surf = ggplot(cv_df[(cv_df$KCNJx_eq_KCNJ2 == 1 |
                             is.na(cv_df$KCNJx_eq_KCNJ2)) &
                            !is.na(cv_df$Clinical_Significance), ],
                    aes(x = surface_score, color = Clinical_Significance)) +
  stat_ecdf() +
  ggthemes::scale_color_tableau() +
  geom_vline(xintercept = benign_sq,
             color = "#4E79A7",
             linetype = 'dashed') +
  geom_vline(xintercept = patho_sq,
             color = "#E15759",
             linetype = 'dashed') +
  ggtitle('cv_df function by clinvar assignment')
```

##OUTPUT: Fig7d-e

```
Fig7de = gridExtra::grid.arrange(dbvar_func, dbvar_surf, nrow = 2)
```

```
## Warning: Removed 28 rows containing non-finite values (stat_ecdf).
## Removed 28 rows containing non-finite values (stat_ecdf).
```

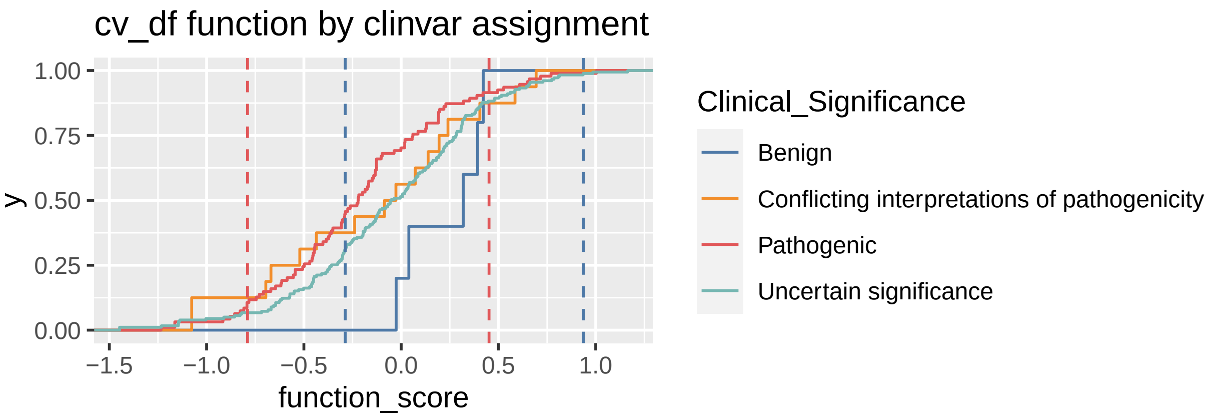

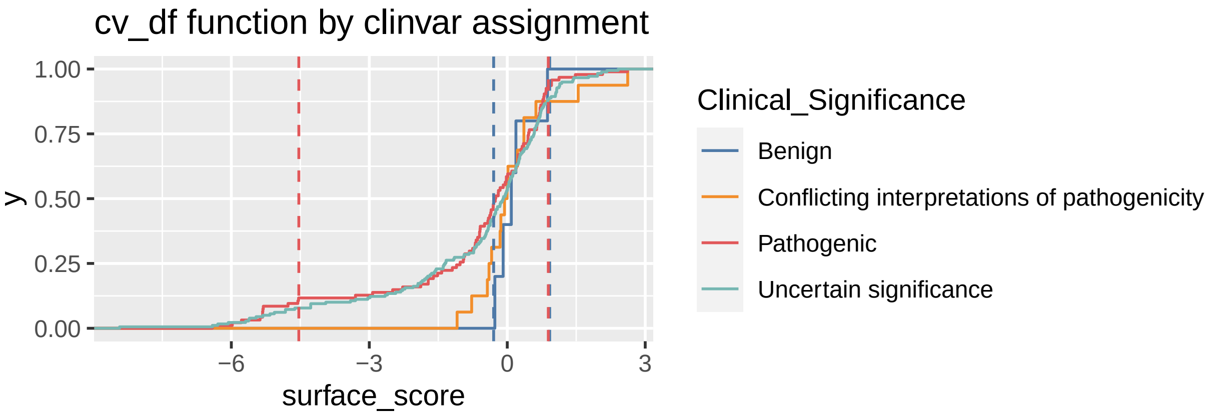


```
Fig7de = annotate_figure(Fig7de, fig.lab = 'Figure 7d-e')
ggsave(
  paste0(fig_path, 'Fig7de.pdf'),
  plot = Fig7de,
  width = 8,
  height = 11,
  units = 'in'
)
```

##OUTPUT: Fig7c (KCNJ2 VUS mechanism prediction)

```
gof_s = cv_df[cv_df$Clinical_Significance %in%
                c('Conflicting interpretations of pathogenicity',
                                                  'Uncertain significance') &
                cv_df$Gene == 'KCNJ2' &
                cv_df$surface_score >= benign_sq[2] &
                cv_df$surface_score >= patho_sq[2], ]
gof_s = gof_s[!is.na(gof_s$position), ]
gof_s$VUSpred = rep('gof_s', nrow(gof_s))

lof_s = cv_df[cv_df$Clinical_Significance %in%
                c('Conflicting interpretations of pathogenicity',
                                                  'Uncertain significance') &
```

```
                cv_df$Gene == 'KCNJ2' &
                cv_df$surface_score <= benign_sq[1], ]
lof_s = lof_s[!is.na(lof_s$position), ]
lof_s$VUSpred = rep('lof_s', nrow(lof_s))

gof_f = cv_df[cv_df$Clinical_Significance %in%
                c('Conflicting interpretations of pathogenicity',
                                                'Uncertain significance') &
                cv_df$Gene == 'KCNJ2' &
                cv_df$function_score >= benign_fq[2], ]
gof_f = gof_f[!is.na(gof_f$position), ]
gof_f$VUSpred = rep('gof_f', nrow(gof_f))
lof_f = cv_df[cv_df$Clinical_Significance %in%
                c('Conflicting interpretations of pathogenicity',
                                                'Uncertain significance') &
                cv_df$Gene == 'KCNJ2' &
                cv_df$function_score <= benign_fq[1], ]
lof_f = lof_f[!is.na(lof_f$position), ]
lof_f$VUSpred = rep('lof_f', nrow(lof_f))

VUSpred = rbind(gof_s, gof_f, lof_s, lof_f)
VUSpred = VUSpred[!is.na(VUSpred$position), ]
```

##OUTPUT: Fig7c (KCNJ2 VUS mechanism prediction)

```
Fig7c_vuspred = ggplot(VUSpred, aes(x = position, fill = VUSpred)) +
  geom_dotplot(method = 'histodot',
               binwidth = 1,
               stackgroups = T) +
  scale_x_continuous(breaks = seq(0, 400, by = 10)) +
  coord_fixed(ratio = 8) +
  ggtitle('Fig7c mechanism of VUS')
ggsave(
  paste0(fig_path, 'Fig7c_vuspred_10per.pdf'),
  plot = Fig7c_vuspred,
  width = 8,
  height = 11,
  units = 'in'
)
```

##VUS variant explained

```
VUSexpl = nrow(VUSpred) /
  nrow(cv_df[cv_df$Clinical_Significance %in%
               c('Uncertain significance',
                 'Conflicting interpretations of pathogenicity') &
               cv_df$Gene == 'KCNJ2',]) * 100
paste0(nrow(VUSpred),
       ' VUS of ',
       nrow(cv_df[cv_df$Clinical_Significance %in% c('Uncertain significance') &
                    cv_df$Gene == 'KCNJ2',]),
       ' explained: ',
       VUSexpl,
       '%')
```

```
## [1] "75 VUS of 89 explained: 75.7575757575758%"
```

```
rep_VUS_expl = as.data.frame(summary(droplevels(as.factor(VUSpred$Mutation))))
rep_VUS_expl$ID = rownames(rep_VUS_expl)
colnames(rep_VUS_expl) = c('n_pred', 'ID')

rep_VUS_expl %>%
  group_by(n_pred) %>%
  summarise(no_rows = length(n_pred))
```

```
## # A tibble: 2 x 2
##   n_pred no_rows
##    <int>   <int>
## 1      1      43
## 2      2      16
```

##predict mechanism of know pathogenic mutations

```
p_gof_s = cv_df[cv_df$Clinical_Significance %in%
                  c('Pathogenic', 'Conflicting interpretations of pathogenicity') &
                  (cv_df$Gene == 'KCNJ2' |
                     cv_df$KCNJx_eq_KCNJ2 == 1) &
                  cv_df$surface_score >= benign_sq[2] &
                  cv_df$surface_SE <= 0.5
                , ]
p_gof_s = p_gof_s[!is.na(p_gof_s$position), ]
p_gof_s$Pathog_pred = rep('p_gof_s', nrow(p_gof_s))

p_lof_s = cv_df[cv_df$Clinical_Significance %in%
                  c('Pathogenic', 'Conflicting interpretations of pathogenicity') &
                  (cv_df$Gene == 'KCNJ2' |
                     cv_df$KCNJx_eq_KCNJ2 == 1) &
                  cv_df$surface_score <= benign_sq[1] &
                  cv_df$surface_SE <= 0.5
                , ]
p_lof_s = p_lof_s[!is.na(p_lof_s$position), ]
p_lof_s$Pathog_pred = rep('p_lof_s', nrow(p_lof_s))

p_gof_f = cv_df[cv_df$Clinical_Significance %in%
                  c('Pathogenic', 'Conflicting interpretations of pathogenicity') &
                  (cv_df$Gene == 'KCNJ2' |
                     cv_df$KCNJx_eq_KCNJ2 == 1) &
                  cv_df$function_score >= benign_fq[2] &
                  cv_df$function_SE <= 0.5
                , ]
p_gof_f = p_gof_f[!is.na(p_gof_f$position), ]
p_gof_f$Pathog_pred = rep('p_gof_f', nrow(p_gof_f))

p_lof_f = cv_df[cv_df$Clinical_Significance %in%
                  c('Pathogenic', 'Conflicting interpretations of pathogenicity') &
                  (cv_df$Gene == 'KCNJ2' |
                     cv_df$KCNJx_eq_KCNJ2 == 1) &
                  cv_df$function_score <= benign_fq[1]  &
                  cv_df$function_SE <= 0.5
                , ]
p_lof_f = p_lof_f[!is.na(p_lof_f$position), ]
```

```
p_lof_f$Pathog_pred = rep('p_lof_f', nrow(p_lof_f))

Pathog_pred = rbind(p_gof_s, p_gof_f, p_lof_s, p_lof_f)

p_unc = cv_df[cv_df$Clinical_Significance %in% c('Pathogenic') &
                (cv_df$Gene == 'KCNJ2' |
                   cv_df$KCNJx_eq_KCNJ2 == 1), ]
p_unc = p_unc[!is.na(p_unc$position), ]
p_unc  = setdiff(p_unc, subset(Pathog_pred, select = -c(Pathog_pred)))
p_unc$Pathog_pred = rep('p_unc', nrow(p_unc))

Pathog_pred = rbind(Pathog_pred, p_unc)
```

##predict mechanism of all mutations

```
cv_df2 = cv_df
cv_df2$Gene[is.na(cv_df2$Gene)] = 'KCNJ2'

p_gof_s_a = cv_df2[(cv_df$Gene == 'KCNJ2' |
                      cv_df$KCNJx_eq_KCNJ2 == 1) &
                     cv_df2$surface_score >= benign_sq[2]
                   , ]
p_gof_s_a = p_gof_s_a[!is.na(p_gof_s_a$position), ]
p_gof_s_a$Pathog_pred = rep('p_gof_s_a', nrow(p_gof_s_a))

p_lof_s_a = cv_df2[(cv_df$Gene == 'KCNJ2' |
                      cv_df$KCNJx_eq_KCNJ2 == 1) &
                     cv_df2$surface_score <= benign_sq[1]
                   , ]
p_lof_s_a = p_lof_s_a[!is.na(p_lof_s_a$position), ]
p_lof_s_a$Pathog_pred = rep('p_lof_s_a', nrow(p_lof_s_a))

p_gof_f_a = cv_df2[(cv_df$Gene == 'KCNJ2' |
                      cv_df$KCNJx_eq_KCNJ2 == 1) &
                     cv_df2$function_score >= benign_fq[2]
                   , ]
p_gof_f_a = p_gof_f_a[!is.na(p_gof_f_a$position), ]
p_gof_f_a$Pathog_pred = rep('p_gof_f_a', nrow(p_gof_f_a))

p_lof_f_a = cv_df2[(cv_df$Gene == 'KCNJ2' |
                      cv_df$KCNJx_eq_KCNJ2 == 1) &
                     cv_df2$function_score <= benign_fq[1]
                   , ]
p_lof_f_a = p_lof_f_a[!is.na(p_lof_f_a$position), ]
p_lof_f_a$Pathog_pred = rep('p_lof_f_a', nrow(p_lof_f_a))

Pathog_pred_a = rbind(p_gof_s_a, p_gof_f_a, p_lof_s_a, p_lof_f_a)
```

##OUTPUT: Fig7c (KCNJ2 know pathgenic mechanism)

```
Fig7c_pathopred = ggplot(Pathog_pred, aes(x = position, fill = Pathog_pred)) +
  geom_dotplot(
    method = 'histodot',
    binwidth = 1,
    size = 2,
```

```r
    stackgroups = T
  ) +
  scale_x_continuous(breaks = seq(0, 400, by = 10)) +
  coord_fixed(ratio = 8) +
  ggtitle('Fig7c mechanism of know pathogenic mutations')
```

```
## Warning: Ignoring unknown parameters: size
```

```r
ggsave(
  paste0(fig_path, 'Fig7c_pathopred_10per.pdf'),
  plot = Fig7c_pathopred,
  width = 8,
  height = 11,
  units = 'in'
)
```

##Pathogenic variant explained

```r
Pathog_pred_wo_punc = Pathog_pred[!Pathog_pred$Pathog_pred == 'p_unc' & Pathog_pred$Gene == 'KCNJ2', ]
n_pathog_expl = length(unique(droplevels(
  interaction(Pathog_pred_wo_punc$mutation, Pathog_pred_wo_punc$position)
)))
n_pathog_KCNJ2 = nrow(dbvar[dbvar$Clinical_Significance %in%
                              c('Pathogenic',
                                'Conflicting interpretations of pathogenicity') &
                              dbvar$Gene == 'KCNJ2',])
paste0(
  n_pathog_expl,
  ' VUS of ',
  n_pathog_KCNJ2,
  ' explained: ',
  n_pathog_expl / n_pathog_KCNJ2 * 100,
  '%'
)
```

```
## [1] "26 VUS of 53 explained: 49.0566037735849%"
```

```r
rep_pathog_expl = as.data.frame(summary(droplevels(
  as.factor(Pathog_pred_wo_punc$Mutation)
), maxsum = 500))
rep_pathog_expl$ID = rownames(rep_pathog_expl)
colnames(rep_pathog_expl) = c('n_pred', 'ID')

rep_pathog_expl %>%
  group_by(n_pred) %>%
  summarise(no_rows = length(n_pred))
```

```
## # A tibble: 2 x 2
##   n_pred no_rows
##    <int>   <int>
## 1      1      20
## 2      2       6
```

##This study vs ClinVar; Odds Ratio of finding Loss of Surface Expression

```r
ts_ps = intersect(p_lof_s$Mutation, p_lof_s_a$Mutation)
ts_pf = intersect(p_lof_s$Mutation, p_lof_f_a$Mutation)
```

```
tf_ps = intersect(p_lof_f$Mutation, p_lof_s_a$Mutation)
tf_pf = intersect(p_lof_f$Mutation, p_lof_f_a$Mutation)

ttable = matrix(
  c(length(ts_ps),
    length(ts_pf),
    length(tf_ps),
    length(tf_pf)),
  nrow = 2,
  dimnames = list(
    Pathogenic = c("LOS", "LOF"),
    Predicted = c("LOS", "LOF")
  )
)

ttable
```

```
##           Predicted
## Pathogenic LOS LOF
##        LOS  36  20
##        LOF  15  39
```

```
fisher.test(ttable, alternative = 'greater')
```

```
##
##  Fisher's Exact Test for Count Data
##
## data:  ttable
## p-value = 0.0001145
## alternative hypothesis: true odds ratio is greater than 1
## 95 percent confidence interval:
##  2.196839      Inf
## sample estimates:
## odds ratio
##   4.608436
```

##OUTPUT: Figure 1-figure supplement 1

```
ClinSigSuppFig_row1 = ggplot(data = cv_kir21[cv_kir21$kir21_FLAG_resno %in% c(0:100) &
                                               !is.na(cv_kir21$KCNJx_MUT_resid),],
                             aes(x = kir21_FLAG_resno, y = KCNJx_MUT_resid,
                                 fill = Clinical_Significance)) +
  geom_tile(color = 'black', size = 0.1) +
  scale_x_continuous(breaks = seq(0, 400, by = 5)) +
  scale_fill_identity(na.value = 'white') +
  coord_fixed(ratio = 1) +
  theme(
    panel.background = element_rect(
      fill = "white",
      colour = "white",
      size = 0.5,
      linetype = "solid"
    ),
    panel.grid.major = element_line(
      size = 0.5,
```

```
      linetype = 'solid',
      colour = "lightgray"
    ),
    panel.grid.minor = element_blank(),
    axis.text.x = element_text(angle = 90, hjust = 1),
    axis.text = element_text(size = 3)
  )


ClinSigSuppFig_row2 = ggplot(data = cv_kir21[cv_kir21$kir21_FLAG_resno %in% c(101:200) &
                                               !is.na(cv_kir21$KCNJx_MUT_resid),],
                             aes(x = kir21_FLAG_resno, y = KCNJx_MUT_resid,
                                 fill = Clinical_Significance)) +
  geom_tile(color = 'black', size = 0.1) +
  scale_x_continuous(breaks = seq(0, 400, by = 5)) +
  scale_fill_identity(na.value = 'white') +
  coord_fixed(ratio = 1) +
  theme(
    panel.background = element_rect(
      fill = "white",
      colour = "white",
      size = 0.5,
      linetype = "solid"
    ),
    panel.grid.major = element_line(
      size = 0.5,
      linetype = 'solid',
      colour = "lightgray"
    ),
    panel.grid.minor = element_blank(),
    axis.text.x = element_text(angle = 90, hjust = 1),
    axis.text = element_text(size = 3)
  )
ClinSigSuppFig_row3 = ggplot(data = cv_kir21[cv_kir21$kir21_FLAG_resno %in% c(201:300) &
                                               !is.na(cv_kir21$KCNJx_MUT_resid),],
                             aes(x = kir21_FLAG_resno, y = KCNJx_MUT_resid,
                                 fill = Clinical_Significance)) +
  geom_tile(color = 'black', size = 0.1) +
  scale_x_continuous(breaks = seq(0, 400, by = 5)) +
  scale_fill_identity(na.value = 'white') +
  coord_fixed(ratio = 1) +
  theme(
    panel.background = element_rect(
      fill = "white",
      colour = "white",
      size = 0.5,
      linetype = "solid"
    ),
    panel.grid.major = element_line(
      size = 0.5,
      linetype = 'solid',
      colour = "lightgray"
    ),
```

```
    panel.grid.minor = element_blank(),
    axis.text.x = element_text(angle = 90, hjust = 1),
    axis.text = element_text(size = 3)
  )
ClinSigSuppFig_row4 = ggplot(data = cv_kir21[cv_kir21$kir21_FLAG_resno %in% c(301:400) &
                                               !is.na(cv_kir21$KCNJx_MUT_resid),],
                             aes(x = kir21_FLAG_resno, y = KCNJx_MUT_resid,
                                 fill = Clinical_Significance)) +
  geom_tile(color = 'black', size = 0.1) +
  scale_x_continuous(breaks = seq(0, 400, by = 5)) +
  scale_fill_identity(na.value = 'white') +
  coord_fixed(ratio = 1) +
  theme(
    panel.background = element_rect(
      fill = "white",
      colour = "white",
      size = 0.5,
      linetype = "solid"
    ),
    panel.grid.major = element_line(
      size = 0.5,
      linetype = 'solid',
      colour = "lightgray"
    ),
    panel.grid.minor = element_blank(),
    axis.text.x = element_text(angle = 90, hjust = 1),
    axis.text = element_text(size = 3)
  )

ClinSigSuppFig = ggarrange(
  ClinSigSuppFig_row1,
  ClinSigSuppFig_row2,
  ClinSigSuppFig_row3,
  ClinSigSuppFig_row4,
  nrow = 4,
  ncol = 1
)
annotate_figure(ClinSigSuppFig, fig.lab = 'ClinSigSuppFig')
```

ClinSigSuppFig

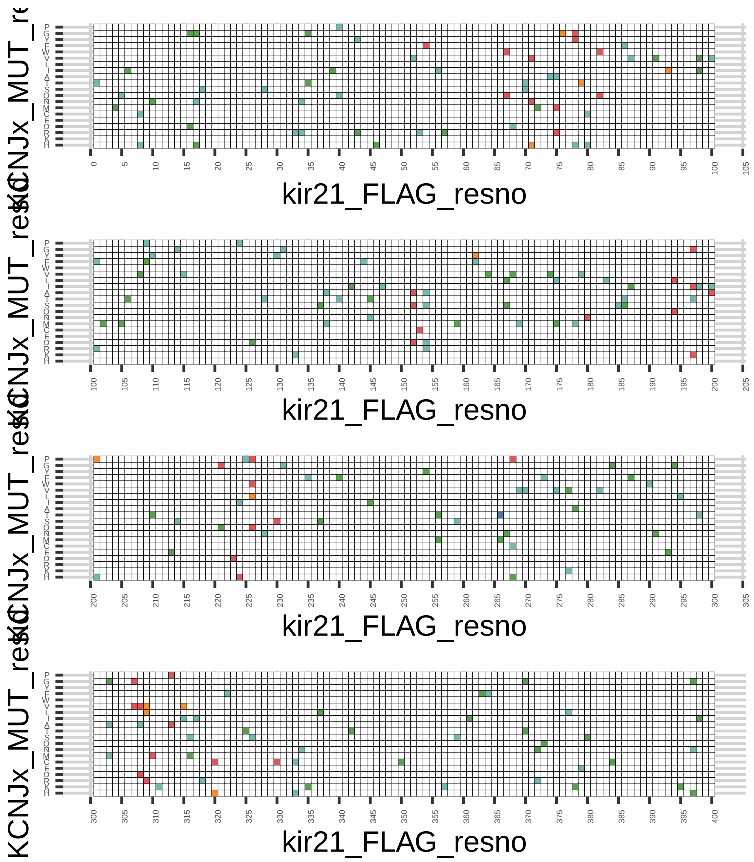


```r
ggsave(
  paste0(fig_path, 'Figure 1-figure supplement 1.pdf'),
  plot = ClinSigSuppFig,
  width = 8,
  height = 11,
  units = 'in'
)
print(paste0(
  'Clinvar & Gnomad has data for ',
  round(
    sum(cv_kir21$Clinical_Significance != 'white') / nrow(cv_kir21),
    3
  ) * 100,
  '% of KCNJ2 positions'
))
```

```
## [1] "Clinvar & Gnomad has data for 2.7% of KCNJ2 positions"
```

```r
print(paste0(
  'Clinvar & Gnomad has data for ',
  round(1 - (sum(
    is.na(cv_kir21$Clinical_Significance)
  ) / nrow(cv_kir21)), 3) * 100,
  '% of KCNJ2 positions'
))
```

```
## [1] "Clinvar & Gnomad has data for 100% of KCNJ2 positions"
```

##OUTPUT: Figure 1-figure supplement 2a

```
Baseline_SuppFig_row1 = ggplot(data = baseline[baseline$position %in% c(1:100), ],
                              aes(x = position, y = mutation, fill = log10(count))) +
  geom_tile(aes(color = as.factor(is.wt)), size = 0.2) +
  scale_fill_continuous_divergingx(
    palette = 'PuOr',
    mid = 0,
    rev = TRUE,
    na.value = '#FFA1E0'
  ) +
  scale_color_manual(values = c(NA, 'green')) +
  scale_x_continuous(breaks = seq(0, 400, by = 5)) +
  coord_fixed(ratio = 1) +
  theme(
    panel.background = element_rect(
      fill = "white",
      colour = "white",
      size = 0.5,
      linetype = "solid"
    ),
    panel.grid.major = element_line(
      size = 0.5,
      linetype = 'solid',
      colour = "lightgray"
    ),
    panel.grid.minor = element_blank(),
    axis.text.x = element_text(angle = 90, hjust = 1),
    axis.text = element_text(size = 7)
  )


Baseline_SuppFig_row2 = ggplot(data = baseline[baseline$position %in% c(101:200), ],
                              aes(x = position, y = mutation, fill = log10(count))) +
  geom_tile(aes(color = as.factor(is.wt)), size = 0.2) +
  scale_fill_continuous_divergingx(
    palette = 'PuOr',
    mid = 0,
    rev = TRUE,
    na.value = '#FFA1E0'
  ) +
  scale_color_manual(values = c(NA, 'green')) +
  scale_x_continuous(breaks = seq(0, 400, by = 5)) +
  coord_fixed(ratio = 1) +
  theme(
    panel.background = element_rect(
      fill = "white",
      colour = "white",
      size = 0.5,
      linetype = "solid"
    ),
    panel.grid.major = element_line(
      size = 0.5,
      linetype = 'solid',
```

```
      colour = "lightgray"
    ),
    panel.grid.minor = element_blank(),
    axis.text.x = element_text(angle = 90, hjust = 1),
    axis.text = element_text(size = 7)
  )

Baseline_SuppFig_row3 = ggplot(data = baseline[baseline$position %in% c(201:300), ],
                               aes(x = position, y = mutation, fill = log10(count))) +
  geom_tile(aes(color = as.factor(is.wt)), size = 0.2) +
  scale_fill_continuous_divergingx(
    palette = 'PuOr',
    mid = 0,
    rev = TRUE,
    na.value = '#FFA1E0'
  ) +
  scale_color_manual(values = c(NA, 'green')) +
  scale_x_continuous(breaks = seq(0, 400, by = 5)) +
  coord_fixed(ratio = 1) +
  theme(
    panel.background = element_rect(
      fill = "white",
      colour = "white",
      size = 0.5,
      linetype = "solid"
    ),
    panel.grid.major = element_line(
      size = 0.5,
      linetype = 'solid',
      colour = "lightgray"
    ),
    panel.grid.minor = element_blank(),
    axis.text.x = element_text(angle = 90, hjust = 1),
    axis.text = element_text(size = 7)
  )

Baseline_SuppFig_row4 = ggplot(data = baseline[baseline$position %in% c(301:400), ],
                               aes(x = position, y = mutation, fill = log10(count))) +
  geom_tile(aes(color = as.factor(is.wt)), size = 0.2) +
  scale_fill_continuous_divergingx(
    palette = 'PuOr',
    mid = 0,
    rev = TRUE,
    na.value = '#FFA1E0'
  ) +
  scale_color_manual(values = c(NA, 'green')) +
  scale_x_continuous(breaks = seq(0, 400, by = 5)) +
  coord_fixed(ratio = 1) +
  theme(
    panel.background = element_rect(
      fill = "white",
      colour = "white",
      size = 0.5,
```

```
      linetype = "solid"
    ),
    panel.grid.major = element_line(
      size = 0.5,
      linetype = 'solid',
      colour = "lightgray"
    ),
    panel.grid.minor = element_blank(),
    axis.text.x = element_text(angle = 90, hjust = 1),
    axis.text = element_text(size = 7)
  )


Baseline_SuppFig = ggarrange(
  Baseline_SuppFig_row1,
  Baseline_SuppFig_row2,
  Baseline_SuppFig_row3,
  Baseline_SuppFig_row4,
  nrow = 4,
  ncol = 1
)
Baseline_SuppFig = annotate_figure(Baseline_SuppFig, fig.lab = 'Figure 1-figure supplement 2a')
ggsave(
  paste0(fig_path, 'Figure 1-figure supplement 2a.pdf'),
  plot = Baseline_SuppFig,
  width = 8,
  height = 11,
  units = 'in'
)
```

##OUTPUT: Figure 1-figure supplement 2b

```
baseline_ecdf = ggplot(baseline, aes(x = count)) + stat_ecdf() + scale_x_log10()
baseline_ecdf = annotate_figure(baseline_ecdf, fig.lab = 'Figure 1-figure supplement 2b')
```

```
## Warning: Removed 1389 rows containing non-finite values (stat_ecdf).
```

```
ggsave(
  paste0(fig_path, 'Figure 1-figure supplement 2b.pdf'),
  plot = baseline_ecdf,
  width = 8,
  height = 11,
  units = 'in'
)
```

##OUTPUT: Figure 2-figure supplement 1a

```
NA_venn = ggVennDiagram::ggVennDiagram(list_NA) + scale_fill_distiller(palette = "Reds", direction = 1)
NA_venn = annotate_figure(NA_venn, fig.lab = 'Figure 2-figure supplement 1a')
ggsave(
  paste0(fig_path, 'Figure 2-figure supplement 1a.pdf'),
  plot = NA_venn,
  width = 8,
  height = 11,
  units = 'in'
)
```

##OUTPUT: Figure 2-figure supplement 1b

```
cor.prob(cor_NA[, 3:5]) %>%
  kbl(caption = 'Figure 2-figure supplement 1b') %>%
  kable_paper('hover', full_width = F, html_font = 'helvetica') %>%
  save_kable(file = paste0(fig_path, 'Figure 2-figure supplement 1b.pdf'))
```

##OUTPUT: Figure 2-figure supplement 2

```
surf_rep_corr = ggarrange(d_neg_rep,
                          d_low_rep,
                          d_up_rep,
                          d_high_rep,
                          ncol = 2,
                          nrow = 2)
```

```
## `geom_smooth()` using formula 'y ~ x'

## Warning: Removed 369 rows containing non-finite values (stat_smooth).

## Warning: Removed 369 rows containing non-finite values (stat_cor).

## Warning: Removed 369 rows containing missing values (geom_point).

## `geom_smooth()` using formula 'y ~ x'

## Warning: Removed 233 rows containing non-finite values (stat_smooth).

## Warning: Removed 233 rows containing non-finite values (stat_cor).

## Warning: Removed 233 rows containing missing values (geom_point).

## `geom_smooth()` using formula 'y ~ x'

## Warning: Removed 443 rows containing non-finite values (stat_smooth).

## Warning: Removed 443 rows containing non-finite values (stat_cor).

## Warning: Removed 443 rows containing missing values (geom_point).

## `geom_smooth()` using formula 'y ~ x'

## Warning: Removed 634 rows containing non-finite values (stat_smooth).

## Warning: Removed 634 rows containing non-finite values (stat_cor).

## Warning: Removed 634 rows containing missing values (geom_point).
```

```
surf_rep_corr = annotate_figure(surf_rep_corr, fig.lab = 'Figure 2-figure supplement 2')
ggsave(
  paste0(fig_path, 'Figure 2-figure supplement 2.pdf'),
  plot = surf_rep_corr,
  width = 8,
  height = 11,
  units = 'in'
)
```

##OUTPUT: Figure 2-figure supplement 3

```
surf_depth = ggplot(d_count_melt, aes(x = as.numeric(value), color = variable)) + stat_ecdf() +
  facet_wrap(cond ~ .) +
  scale_x_log10() +
  geom_vline(xintercept = 30)
surf_depth = annotate_figure(surf_depth, fig.lab = 'Figure 2-figure supplement 3')
```

```
## Warning: Removed 1679 rows containing non-finite values (stat_ecdf).
```

```
ggsave(
  paste0(fig_path, 'Figure 2-figure supplement 3.pdf'),
  plot = surf_depth,
  width = 8,
  height = 11,
  units = 'in'
)
```

##OUTPUT: Figure 2-figure supplement 5

```
SurfSE_row1 = ggplot(data = kir21_surf[kir21_surf$position %in% c(1:100), ],
                     aes(x = position, y = mutation, fill = surface_SE)) +
  geom_tile(aes(color = as.factor(is.wt)), size = 0.2) +
  scale_fill_continuous_divergingx(
    palette = 'PuOr',
    mid = 0,
    rev = TRUE,
    na.value = 'magenta'
  ) +
  scale_color_manual(values = c(NA, 'green')) +
  scale_x_continuous(breaks = seq(0, 400, by = 5)) +
  coord_fixed(ratio = 1) +
  theme(
    panel.background = element_rect(
      fill = "white",
      colour = "white",
      size = 0.5,
      linetype = "solid"
    ),
    panel.grid.major = element_line(
      size = 0.5,
      linetype = 'solid',
      colour = "lightgray"
    ),
    panel.grid.minor = element_blank(),
    axis.text.x = element_text(angle = 90, hjust = 1),
    axis.text = element_text(size = 7)
  )
SurfSE_row2 = ggplot(data = kir21_surf[kir21_surf$position %in% c(101:200), ],
                     aes(x = position, y = mutation, fill = surface_SE)) +
  geom_tile(aes(color = as.factor(is.wt)), size = 0.2) +
  scale_fill_continuous_divergingx(
    palette = 'PuOr',
    mid = 0,
    rev = TRUE,
    na.value = 'magenta'
  ) +
  scale_color_manual(values = c(NA, 'green')) +
  scale_x_continuous(breaks = seq(0, 400, by = 5)) +
  coord_fixed(ratio = 1) +
  theme(
    panel.background = element_rect(
      fill = "white",
```

```
      colour = "white",
      size = 0.5,
      linetype = "solid"
    ),
    panel.grid.major = element_line(
      size = 0.5,
      linetype = 'solid',
      colour = "lightgray"
    ),
    panel.grid.minor = element_blank(),
    axis.text.x = element_text(angle = 90, hjust = 1),
    axis.text = element_text(size = 7)
  )
SurfSE_row3 = ggplot(data = kir21_surf[kir21_surf$position %in% c(201:300), ],
                     aes(x = position, y = mutation, fill = surface_SE)) +
  geom_tile(aes(color = as.factor(is.wt)), size = 0.2) +
  scale_fill_continuous_divergingx(
    palette = 'PuOr',
    mid = 0,
    rev = TRUE,
    na.value = 'magenta'
  ) +
  scale_color_manual(values = c(NA, 'green')) +
  scale_x_continuous(breaks = seq(0, 400, by = 5)) +
  coord_fixed(ratio = 1) +
  theme(
    panel.background = element_rect(
      fill = "white",
      colour = "white",
      size = 0.5,
      linetype = "solid"
    ),
    panel.grid.major = element_line(
      size = 0.5,
      linetype = 'solid',
      colour = "lightgray"
    ),
    panel.grid.minor = element_blank(),
    axis.text.x = element_text(angle = 90, hjust = 1),
    axis.text = element_text(size = 7)
  )
SurfSE_row4 = ggplot(data = kir21_surf[kir21_surf$position %in% c(301:400), ],
                     aes(x = position, y = mutation, fill = surface_SE)) +
  geom_tile(aes(color = as.factor(is.wt)), size = 0.2) +
  scale_fill_continuous_divergingx(
    palette = 'PuOr',
    mid = 0,
    rev = TRUE,
    na.value = 'magenta'
  ) +
  scale_color_manual(values = c(NA, 'green')) +
  scale_x_continuous(breaks = seq(0, 400, by = 5)) +
  coord_fixed(ratio = 1) +
```

```
  theme(
    panel.background = element_rect(
      fill = "white",
      colour = "white",
      size = 0.5,
      linetype = "solid"
    ),
    panel.grid.major = element_line(
      size = 0.5,
      linetype = 'solid',
      colour = "lightgray"
    ),
    panel.grid.minor = element_blank(),
    axis.text.x = element_text(angle = 90, hjust = 1),
    axis.text = element_text(size = 7)
  )
```

```
SurfSE_hist = ggplot(kir21_surf, aes(x = surface_SE)) +
  geom_histogram(color = "black", fill = "white", bins = 100) + theme_bw() +
  ggtitle('Figure 2-figure supplement 5a')
ggsave(
  paste0(fig_path, 'Figure 2-figure supplement 5a.pdf'),
  plot = SurfSE_hist,
  width = 8,
  height = 11,
  units = 'in'
)
```

```
## Warning: Removed 893 rows containing non-finite values (stat_bin).
```

```
SurfSE_fig = ggarrange(
  SurfSE_row1,
  SurfSE_row2,
  SurfSE_row3,
  SurfSE_row4,
  nrow = 4,
  ncol = 1
)
annotate_figure(SurfSE_fig, fig.lab = 'Figure 2-figure supplement 5b')
```

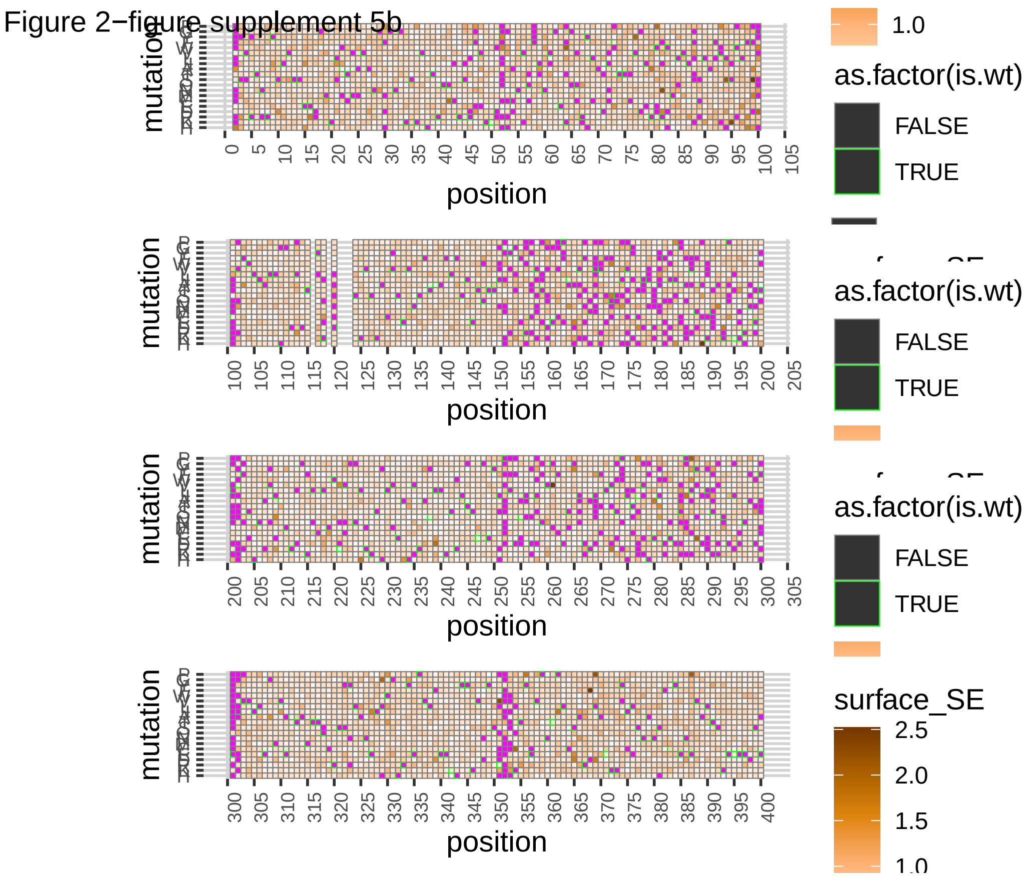


```
ggsave(
  paste0(fig_path, 'Figure 2-figure supplement 5b.pdf'),
  plot = SurfSE_fig,
  width = 8,
  height = 11,
  units = 'in'
)
```

##OUTPUT: Figure 4-figure supplement 1

```
func_rep_corr = ggarrange(f_neg_plot,
                          f_low_plot,
                          f_high_plot,
                          ncol = 2,
                          nrow = 2)
```

```
## `geom_smooth()` using formula 'y ~ x'

## Warning: Removed 610 rows containing non-finite values (stat_smooth).

## Warning: Removed 610 rows containing non-finite values (stat_cor).

## Warning: Removed 610 rows containing missing values (geom_point).

## `geom_smooth()` using formula 'y ~ x'

## Warning: Removed 285 rows containing non-finite values (stat_smooth).

## Warning: Removed 285 rows containing non-finite values (stat_cor).

## Warning: Removed 285 rows containing missing values (geom_point).
```

```
## `geom_smooth()` using formula 'y ~ x'

## Warning: Removed 301 rows containing non-finite values (stat_smooth).

## Warning: Removed 301 rows containing non-finite values (stat_cor).

## Warning: Removed 301 rows containing missing values (geom_point).
```

```
func_rep_corr = annotate_figure(func_rep_corr, fig.lab = 'Figure 4-figure supplement 1')
ggsave(
  paste0(fig_path, 'Figure 4-figure supplement 1.pdf'),
  plot = func_rep_corr,
  width = 8,
  height = 11,
  units = 'in'
)
```

##OUTPUT: Figure 4-figure supplement 2

```
func_depth = ggplot(f_count_melt, aes(x = as.numeric(value), color = variable)) + stat_ecdf() +
  facet_wrap(cond ~ .) +
  scale_x_log10() +
  geom_vline(xintercept = 30)
func_depth = annotate_figure(func_depth, fig.lab = 'Figure 4-figure supplement 2')
```

```
## Warning: Removed 1196 rows containing non-finite values (stat_ecdf).
```

```
ggsave(
  paste0(fig_path, 'Figure 4-figure supplement 2.pdf'),
  plot = func_depth,
  width = 8,
  height = 11,
  units = 'in'
)
```

##OUTPUT: Figure 4-figure supplement 4a

```
funcSE_hist = ggplot(kir21_func, aes(x = function_SE)) +
  geom_histogram(color = "black", fill = "white", bins = 100) + theme_bw() +
  ggtitle('Figure 4-figure supplement 4a')
ggsave(
  paste0(fig_path, 'Figure 4-figure supplement 4a.pdf'),
  plot = funcSE_hist,
  width = 8,
  height = 11,
  units = 'in'
)
```

```
## Warning: Removed 847 rows containing non-finite values (stat_bin).
```

##OUTPUT: Figure 4-figure supplement 4b

```
funcSE_row1 = ggplot(data = kir21_func[kir21_func$position %in% c(1:100), ],
                     aes(x = position, y = mutation, fill = function_SE)) +
  geom_tile(aes(color = as.factor(is.wt)), size = 0.2) +
  scale_fill_continuous_divergingx(
    palette = 'PuOr',
    mid = 0,
    rev = TRUE,
    na.value = 'magenta'
```

```
  ) +
  scale_color_manual(values = c(NA, 'green')) +
  scale_x_continuous(breaks = seq(0, 400, by = 5)) +
  coord_fixed(ratio = 1) +
  theme(
    panel.background = element_rect(
      fill = "white",
      colour = "white",
      size = 0.5,
      linetype = "solid"
    ),
    panel.grid.major = element_line(
      size = 0.5,
      linetype = 'solid',
      colour = "lightgray"
    ),
    panel.grid.minor = element_blank(),
    axis.text.x = element_text(angle = 90, hjust = 1),
    axis.text = element_text(size = 7)
  )
funcSE_row2 = ggplot(data = kir21_func[kir21_func$position %in% c(101:200), ],
                     aes(x = position, y = mutation, fill = function_SE)) +
  geom_tile(aes(color = as.factor(is.wt)), size = 0.2) +
  scale_fill_continuous_divergingx(
    palette = 'PuOr',
    mid = 0,
    rev = TRUE,
    na.value = 'magenta'
  ) +
  scale_color_manual(values = c(NA, 'green')) +
  scale_x_continuous(breaks = seq(0, 400, by = 5)) +
  coord_fixed(ratio = 1) +
  theme(
    panel.background = element_rect(
      fill = "white",
      colour = "white",
      size = 0.5,
      linetype = "solid"
    ),
    panel.grid.major = element_line(
      size = 0.5,
      linetype = 'solid',
      colour = "lightgray"
    ),
    panel.grid.minor = element_blank(),
    axis.text.x = element_text(angle = 90, hjust = 1),
    axis.text = element_text(size = 7)
  )
funcSE_row3 = ggplot(data = kir21_func[kir21_func$position %in% c(201:300), ],
                     aes(x = position, y = mutation, fill = function_SE)) +
  geom_tile(aes(color = as.factor(is.wt)), size = 0.2) +
  scale_fill_continuous_divergingx(
    palette = 'PuOr',
```

```
    mid = 0,
    rev = TRUE,
    na.value = 'magenta'
  ) +
  scale_color_manual(values = c(NA, 'green')) +
  scale_x_continuous(breaks = seq(0, 400, by = 5)) +
  coord_fixed(ratio = 1) +
  theme(
    panel.background = element_rect(
      fill = "white",
      colour = "white",
      size = 0.5,
      linetype = "solid"
    ),
    panel.grid.major = element_line(
      size = 0.5,
      linetype = 'solid',
      colour = "lightgray"
    ),
    panel.grid.minor = element_blank(),
    axis.text.x = element_text(angle = 90, hjust = 1),
    axis.text = element_text(size = 7)
  )
funcSE_row4 = ggplot(data = kir21_func[kir21_func$position %in% c(301:400), ],
                     aes(x = position, y = mutation, fill = function_SE)) +
  geom_tile(aes(color = as.factor(is.wt)), size = 0.2) +
  scale_fill_continuous_divergingx(
    palette = 'PuOr',
    mid = 0,
    rev = TRUE,
    na.value = 'magenta'
  ) +
  scale_color_manual(values = c(NA, 'green')) +
  scale_x_continuous(breaks = seq(0, 400, by = 5)) +
  coord_fixed(ratio = 1) +
  theme(
    panel.background = element_rect(
      fill = "white",
      colour = "white",
      size = 0.5,
      linetype = "solid"
    ),
    panel.grid.major = element_line(
      size = 0.5,
      linetype = 'solid',
      colour = "lightgray"
    ),
    panel.grid.minor = element_blank(),
    axis.text.x = element_text(angle = 90, hjust = 1),
    axis.text = element_text(size = 7)
  )

funcSE = ggarrange(
```

```
  funcSE_row1,
  funcSE_row2,
  funcSE_row3,
  funcSE_row4,
  nrow = 4,
  ncol = 1
)
funcSE = annotate_figure(funcSE, fig.lab = 'Figure 4-figure supplement 4b')
ggsave(
  paste0(fig_path, 'Figure 4-figure supplement 4b.pdf'),
  plot = funcSE,
  width = 8,
  height = 11,
  units = 'in'
)
```

```
c_3spi = read.csv('./input_data/contact_3spi.txt',
                  header = T,
                  sep = '\t')
c_6m84 = read.csv('./input_data/contact_6m84.txt',
                  header = T,
                  sep = '\t')

c_3spi$sub_int = ifelse(c_3spi$Chain1 != c_3spi$Chain2, 1, 0)
c_6m84$sub_int = ifelse(c_6m84$Chain1 != c_6m84$Chain2, 1, 0)

colselect = c('Chain1', 'ResNum1', 'Chain2', 'ResNum2')
c1 = dplyr::intersect(c_3spi[c_3spi$sub_int == 1, colselect],
                      c_6m84[c_6m84$sub_int == 1, colselect])
c2 = dplyr::intersect(c_3spi[c_3spi$sub_int == 0, colselect],
                      c_6m84[c_6m84$sub_int == 0, colselect])


c1_rb = as.data.frame(rbind(as.matrix(c1[, c('Chain1', 'ResNum1')]),
                            as.matrix(c1[, c('Chain2', 'ResNum2')])))
colnames(c1_rb) = c('chain', 'resno')

c2_rb = as.data.frame(rbind(as.matrix(c2[, c('Chain1', 'ResNum1')]),
                            as.matrix(c2[, c('Chain2', 'ResNum2')])))
colnames(c2_rb) = c('chain', 'resno')


c1_rb_un = irk$Kir2_1_FLAG_Resno_mus[match(unique(as.numeric(c1_rb$resno)),
                                           irk$Kir2_2_Resno_NC)]
c1_rb_un = c1_rb_un[!is.na(c1_rb_un)]
c2_rb_un = irk$Kir2_1_FLAG_Resno_mus[match(unique(as.numeric(c2_rb$resno)),
                                           irk$Kir2_2_Resno_NC)]
c2_rb_un = c2_rb_un[!is.na(c2_rb_un)]
c3_total = union(c1_rb_un, c2_rb_un)

inter_int = setdiff(c3_total, c2_rb_un)
intra_int = setdiff(c3_total, c1_rb_un)
both_int = intersect(c1_rb_un, c2_rb_un)
```

```r
df$inter_int = rep(0, nrow(df))
df$intra_int = rep(0, nrow(df))
df$both_int = rep(0, nrow(df))


df$inter_int[!is.na(match(df$position, inter_int))] = 1
df$intra_int[!is.na(match(df$position, intra_int))] = 1
df$both_int[!is.na(match(df$position, both_int))] = 1
df$ctd = ifelse(df$position %in% c(76:190), 0, 1)
df$ctd_contact = interaction(df$ctd, df$inter_int, df$intra_int, df$both_int)

ks.test(df$surface_score[df$ctd_contact == '1.0.1.0'],
        df$surface_score[df$ctd_contact == '1.0.0.1'])
```

```
##
##  Two-sample Kolmogorov-Smirnov test
##
## data:  df$surface_score[df$ctd_contact == "1.0.1.0"] and df$surface_score[df$ctd_contact == "1.0.0.1
## D = 0.16846, p-value < 2.2e-16
## alternative hypothesis: two-sided
```

```r
ks.test(df$function_score[df$ctd_contact == '1.0.0.1'],
        df$function_score[df$ctd_contact == '1.0.1.0'])
```

```
##
##  Two-sample Kolmogorov-Smirnov test
##
## data:  df$function_score[df$ctd_contact == "1.0.0.1"] and df$function_score[df$ctd_contact == "1.0.1
## D = 0.12907, p-value = 3.713e-10
## alternative hypothesis: two-sided
```

##OUTPUT: Figure 5-figure supplement 1

```r
contact_ecdf1 = ggplot(df[df$ctd == 1,], aes(x = surface_score, color = ctd_contact)) +
  stat_ecdf() +
  theme_classic()
contact_ecdf2 = ggplot(df[df$ctd == 1,], aes(x = function_score, color = ctd_contact)) +
  stat_ecdf() +
  theme_classic()
contact_ecdf = ggarrange(contact_ecdf1,
                         contact_ecdf2,
                         nrow = 2,
                         ncol = 1)
```

```
## Warning: Removed 652 rows containing non-finite values (stat_ecdf).
## Removed 652 rows containing non-finite values (stat_ecdf).
```

```r
ggsave(
  paste0(fig_path, 'Figure 5-figure supplement 1.pdf'),
  plot = contact_ecdf,
  width = 8,
  height = 11,
  units = 'in'
)
```

##OUTPUT: Figure 7-figure supplement 1

```
ttable %>%
  kbl(caption = 'Figure 7-figure supplement 1') %>%
  kable_paper('hover', full_width = F, html_font = 'helvetica') %>%
  save_kable(file = paste0(fig_path, 'Figure 7-figure supplement 1.pdf'))
```

##OUTPUT: Supplementary File 1

```
dbvar_output = data.frame(
  Variant_ID = seq(1:nrow(dbvar)),
  Gene = dbvar$Gene,
  Mutation = dbvar$Mutation,
  Source = dbvar$Source,
  Clinical_Significance = dbvar$Clinical_Significance,
  ClinVar_ID = dbvar$ClinVar_ID,
  Condition = dbvar$Condition,
  kir21_resno = dbvar$kir21_resno,
  kir21_resid = dbvar$kir21_resid,
  kir21_FLAG_resno = dbvar$kir21_FLAG_resno,
  KCNJx_eq_KCNJ2 = dbvar$KCNJx_eq_KCNJ2
)
write.csv(dbvar_output, paste0(fig_path, 'Supplementary File 1.csv'))
```

##OUTPUT: Figure 2-figure supplement 4

```
SuppTable1b = summary_table(d_count_melt,
                            summaries = read_count_summary,
                            by = c('variable', 'cond'))
SuppTable1b %>%
  kbl(caption = 'Figure 2-figure supplement 4') %>%
  kable_paper('hover', full_width = F, html_font = 'helvetica') %>%
  save_kable(file = paste0(fig_path, 'Figure 2-figure supplement 4.pdf'))
```

##OUTPUT: Figure 4-figure supplement 3

```
SuppTable1c = summary_table(f_count_melt,
                            summaries = read_count_summary,
                            by = c('variable', 'cond'))
SuppTable1c %>%
  kbl(caption = 'Figure 4-figure supplement 3') %>%
  kable_paper('hover', full_width = F, html_font = 'helvetica') %>%
  save_kable(file = paste0(fig_path, 'Figure 4-figure supplement 3.pdf'))
```

##OUTPUT: Supplementary File 3

```
VUSpred_output = data.frame(
  Variant_ID = seq(1:nrow(VUSpred)),
  Gene = VUSpred$Gene,
  Mutation = VUSpred$Mutation,
  Source = VUSpred$Source,
  Clinical_Significance = VUSpred$Clinical_Significance,
  ClinVar_ID = VUSpred$ClinVar_ID,
  Condition = VUSpred$Condition,
  kir21_resno = VUSpred$kir21_resno,
  kir21_resid = VUSpred$kir21_resid,
  kir21_FLAG_resno = VUSpred$position,
  predicted_phenotype = VUSpred$VUSpred
```

```
)
write.csv(VUSpred_output, file = paste0(fig_path, 'Supplementary File 3.csv'))
```

##OUTPUT: Supplementary File 4

```
patho_pred_output = data.frame(
  Variant_ID = seq(1:nrow(Pathog_pred_wo_punc)),
  Gene = Pathog_pred_wo_punc$Gene,
  Mutation = Pathog_pred_wo_punc$Mutation,
  Source = Pathog_pred_wo_punc$Source,
  Clinical_Significance = Pathog_pred_wo_punc$Clinical_Significance,
  ClinVar_ID = Pathog_pred_wo_punc$ClinVar_ID,
  Condition = Pathog_pred_wo_punc$Condition,
  kir21_resno = Pathog_pred_wo_punc$kir21_resno,
  kir21_resid = Pathog_pred_wo_punc$kir21_resid,
  kir21_FLAG_resno = Pathog_pred_wo_punc$position,
  predicted_phenotype = Pathog_pred_wo_punc$Pathog_pred
)
write.csv(patho_pred_output, file = paste0(fig_path, 'Supplementary File 4.csv'))
```
