# Supplementary material for: Determinants of trafficking, conduction, and disease within a K+ channel revealed through multiparametric deep mutational scanning
Source: eLife. 2022 May 31;11:e76903. doi: 10.7554/eLife.76903 (PMC9273215; doi:10.7554/eLife.76903)
Supplement: Source data 1. [file elife-76903-data1.zip › SourceData/figure_output/Fig1a.pdf]

Table 1: Fig1a

	Kir2.1	other Kir
Benign	2	23
Conflicting interpretations of pathogenicity	13	30
Pathogenic	40	112
Uncertain significance	107	314
