# Supplementary material for: Determinants of trafficking, conduction, and disease within a K+ channel revealed through multiparametric deep mutational scanning
Source: eLife. 2022 May 31;11:e76903. doi: 10.7554/eLife.76903 (PMC9273215; doi:10.7554/eLife.76903)
Supplement: Source data 1. [file elife-76903-data1.zip › SourceData/figure_output/Figure 2-figure supplement 1b.pdf]

Table 1: Figure 2-figure supplement 1b

	baseline	surface	func
baseline	1.0000000	0.1887416	7.4e-06
surface	0.0511067	1.0000000	0.0e+00
func	0.1730571	-0.2142445	1.0e+00
