# Supplementary material for: Determinants of trafficking, conduction, and disease within a K+ channel revealed through multiparametric deep mutational scanning
Source: eLife. 2022 May 31;11:e76903. doi: 10.7554/eLife.76903 (PMC9273215; doi:10.7554/eLife.76903)
Supplement: Source data 1. [file elife-76903-data1.zip › SourceData/figure_output/Figure 7-figure supplement 1.pdf]

Table 1: Figure 7-figure supplement 1

	LOS	LOF
LOS	36	20
LOF	15	39
