# Supplementary material for: Determinants of trafficking, conduction, and disease within a K+ channel revealed through multiparametric deep mutational scanning
Source: eLife. 2022 May 31;11:e76903. doi: 10.7554/eLife.76903 (PMC9273215; doi:10.7554/eLife.76903)
Supplement: Source data 1. [file elife-76903-data1.zip › SourceData/figure_output/Figure 2-figure supplement 4.pdf]

Table 1: Figure 2-figure supplement 4

	count1.neg (N = 7282)	count2.neg (N = 7282)	count1.low
median	103.0000	174.0000	
mean	342.3466	344.6385	
>30-fold [%]	71.5000	82.2000	
