# Supplementary material for: Determinants of trafficking, conduction, and disease within a K+ channel revealed through multiparametric deep mutational scanning
Source: eLife. 2022 May 31;11:e76903. doi: 10.7554/eLife.76903 (PMC9273215; doi:10.7554/eLife.76903)
Supplement: Source data 1. [file elife-76903-data1.zip › SourceData/figure_output/Figure 4-figure supplement 3.pdf]

Table 1: Figure 4-figure supplement 3

	count1.neg (N = 7168)	count2.neg (N = 7168)	count1.low
median	178.000	165.0000	
mean	281.561	297.1245	
>30-fold [%]	79.200	79.3000	
