# Supplementary figures and images for: Determinants of trafficking, conduction, and disease within a K+ channel revealed through multiparametric deep mutational scanning
Source: eLife. 2022 May 31;11:e76903. doi: 10.7554/eLife.76903 (PMC9273215; doi:10.7554/eLife.76903)

Fig2a Figure 2a

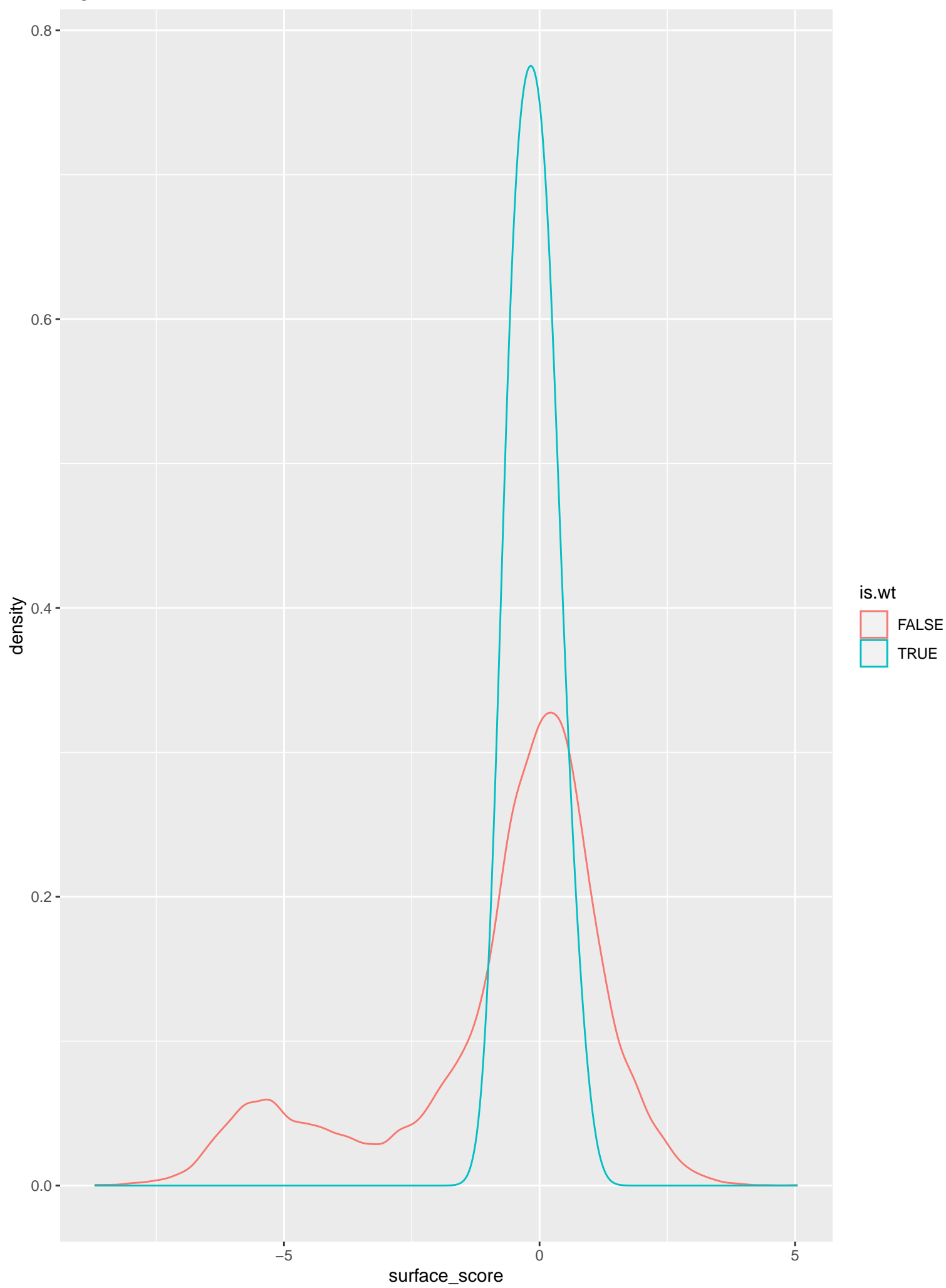

Supplement: Source data 1. [file elife-76903-data1.zip › SourceData/figure_output/Fig2a.pdf]

Fig2b

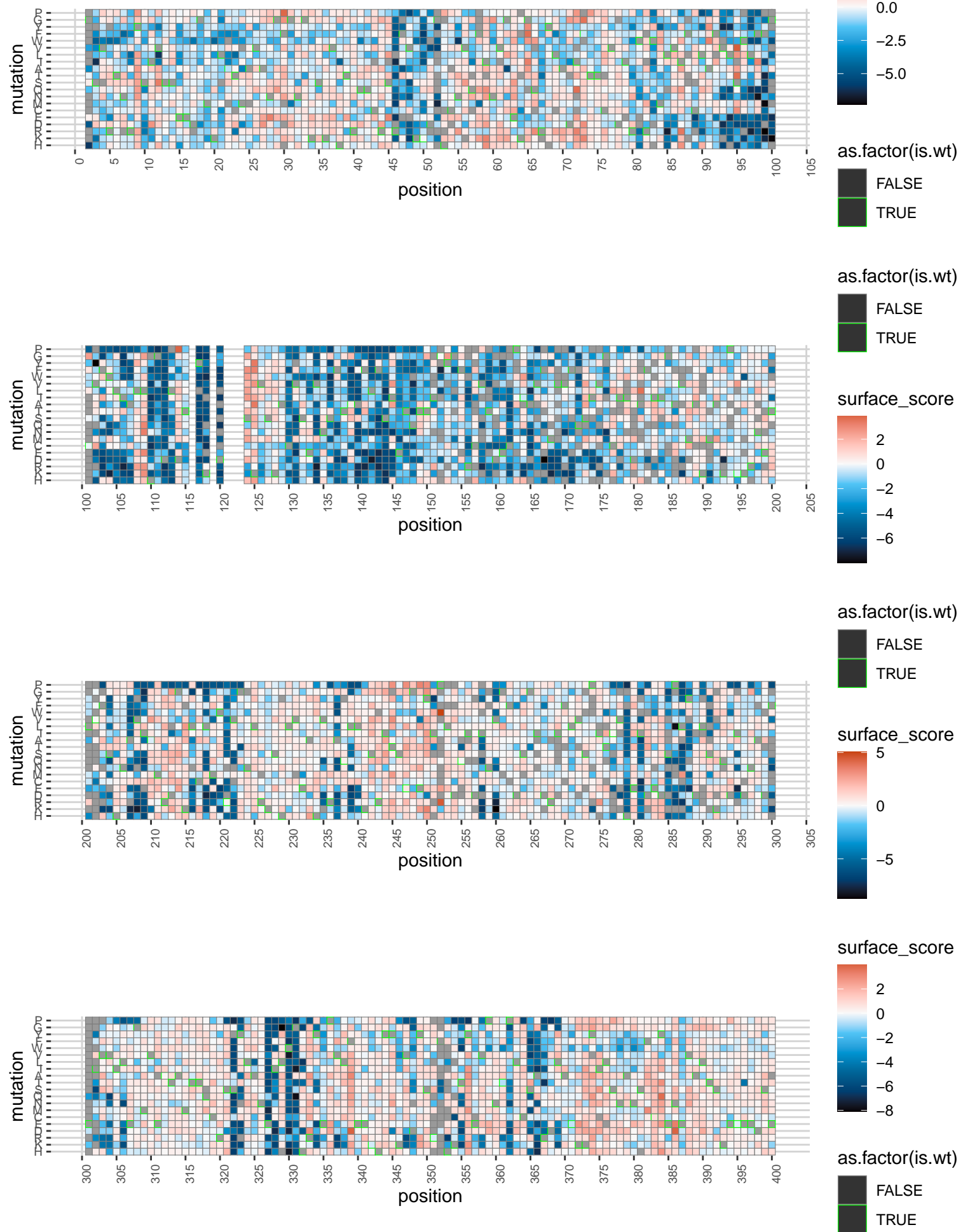

Supplement: Source data 1. [file elife-76903-data1.zip › SourceData/figure_output/Fig2b.pdf]

Fig2g

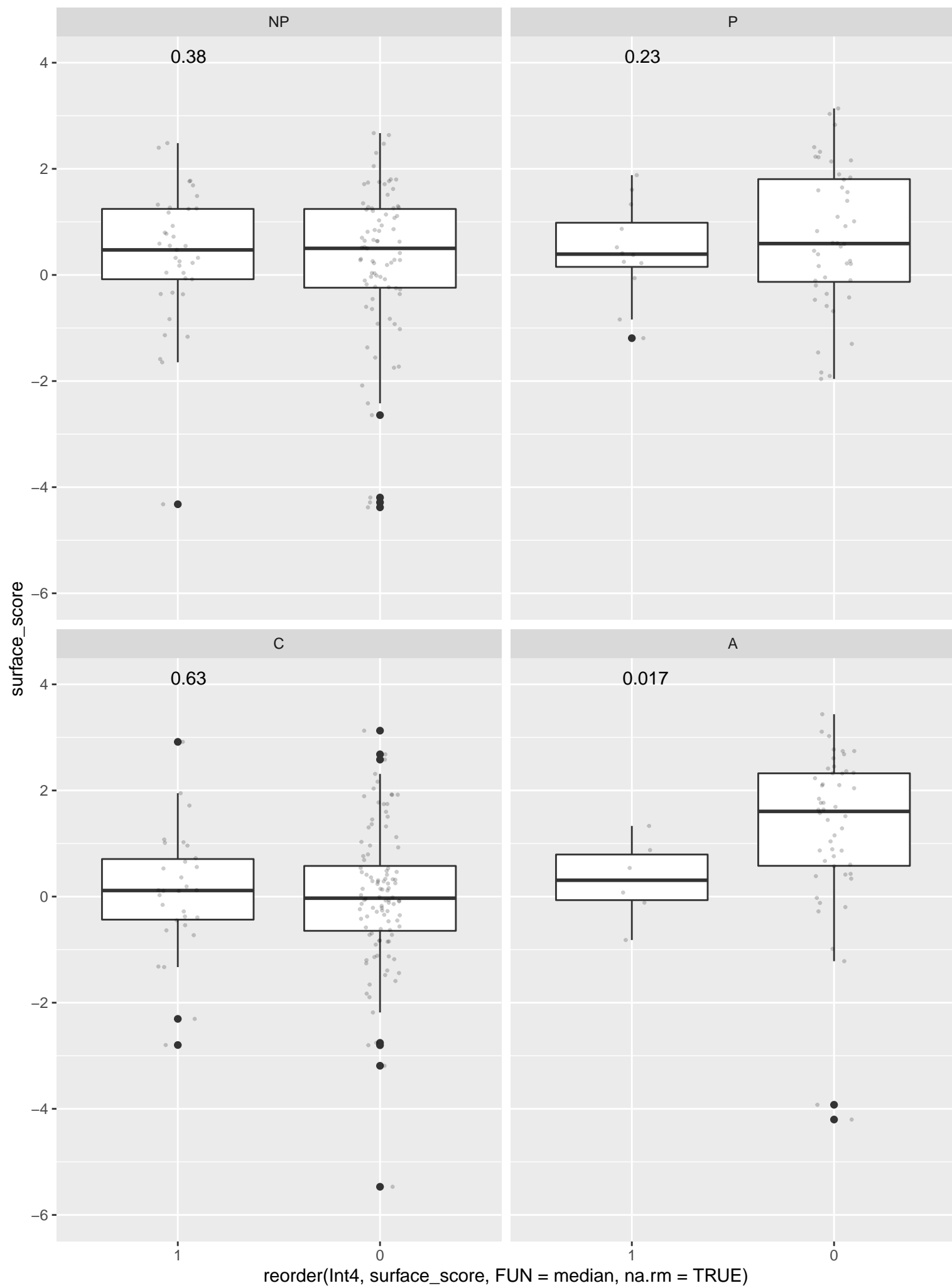

Supplement: Source data 1. [file elife-76903-data1.zip › SourceData/figure_output/Fig2g.pdf]

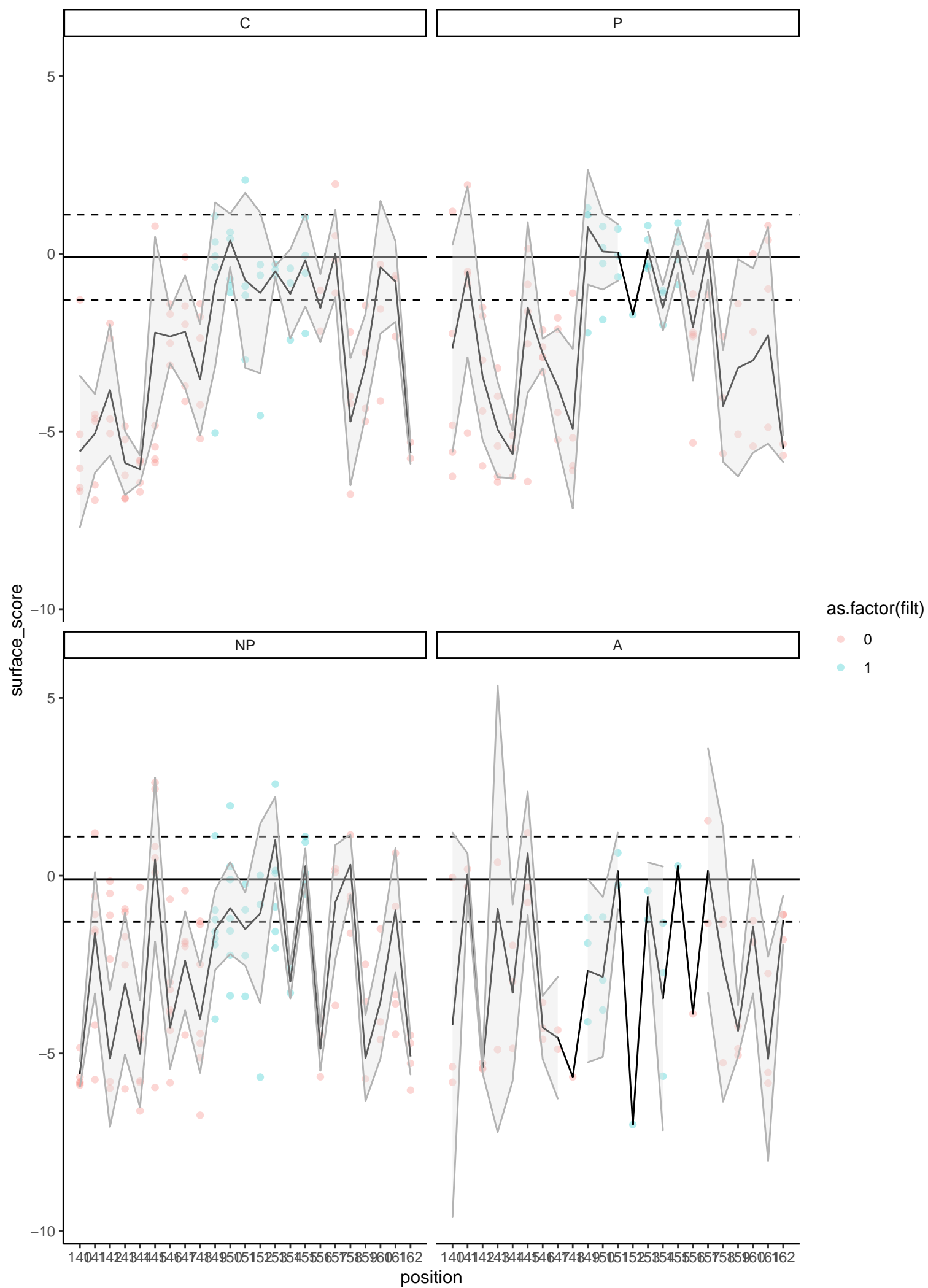

Supplement: Source data 1. [file elife-76903-data1.zip › SourceData/figure_output/Fig3c.pdf]

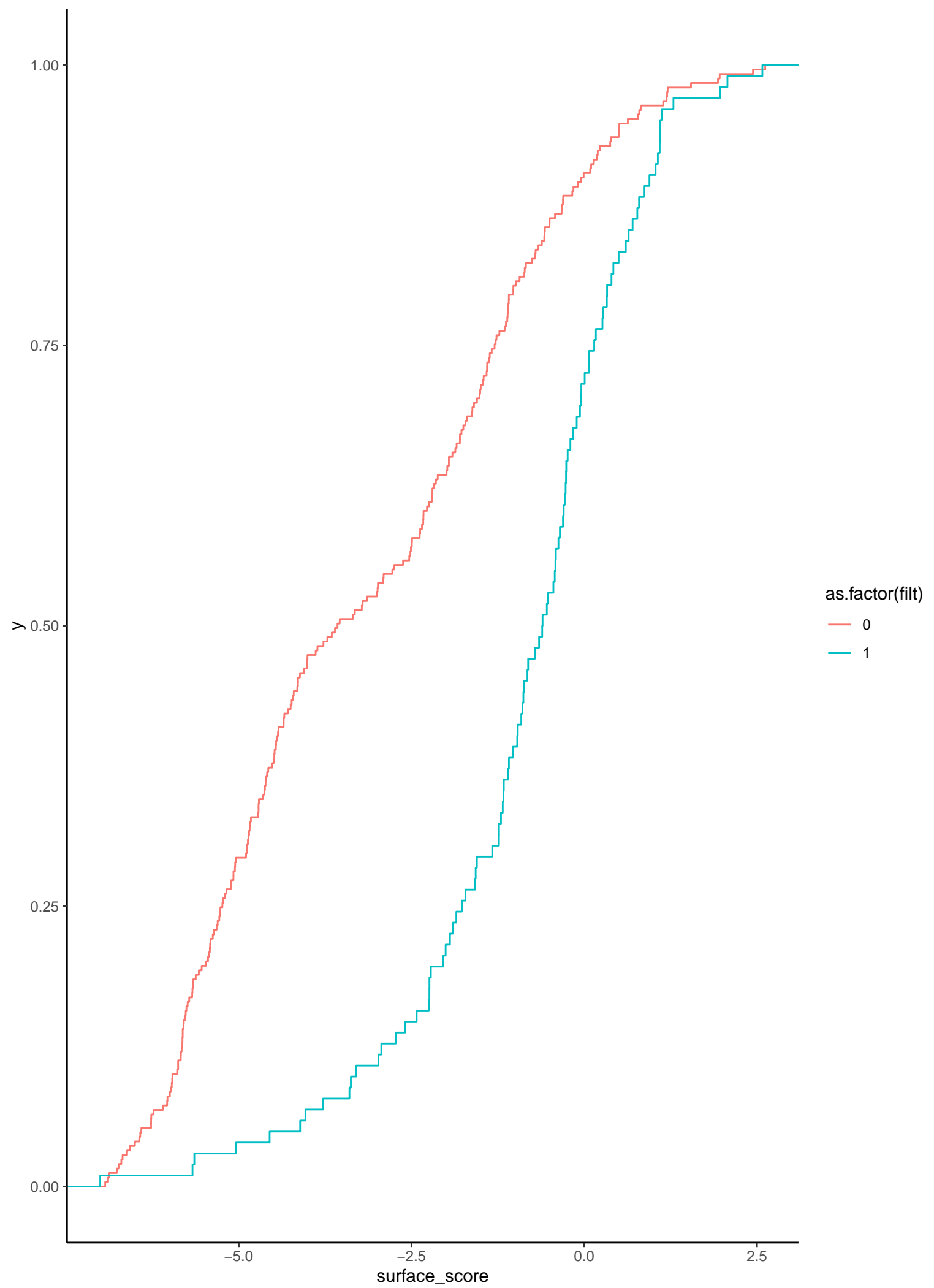

Supplement: Source data 1. [file elife-76903-data1.zip › SourceData/figure_output/Fig3d.pdf]

Figure 4a

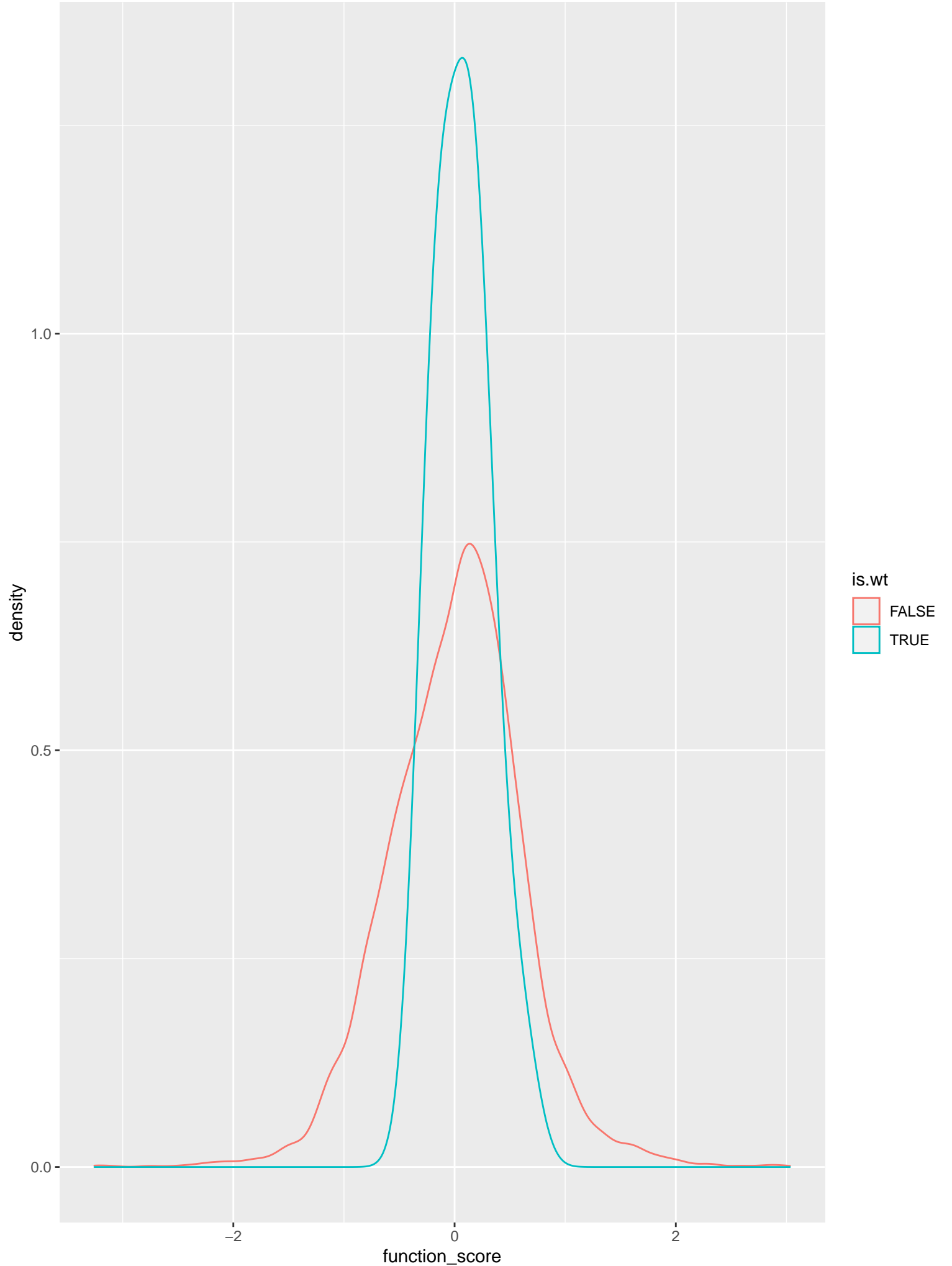

Supplement: Source data 1. [file elife-76903-data1.zip › SourceData/figure_output/Fig4a.pdf]

Fig4c

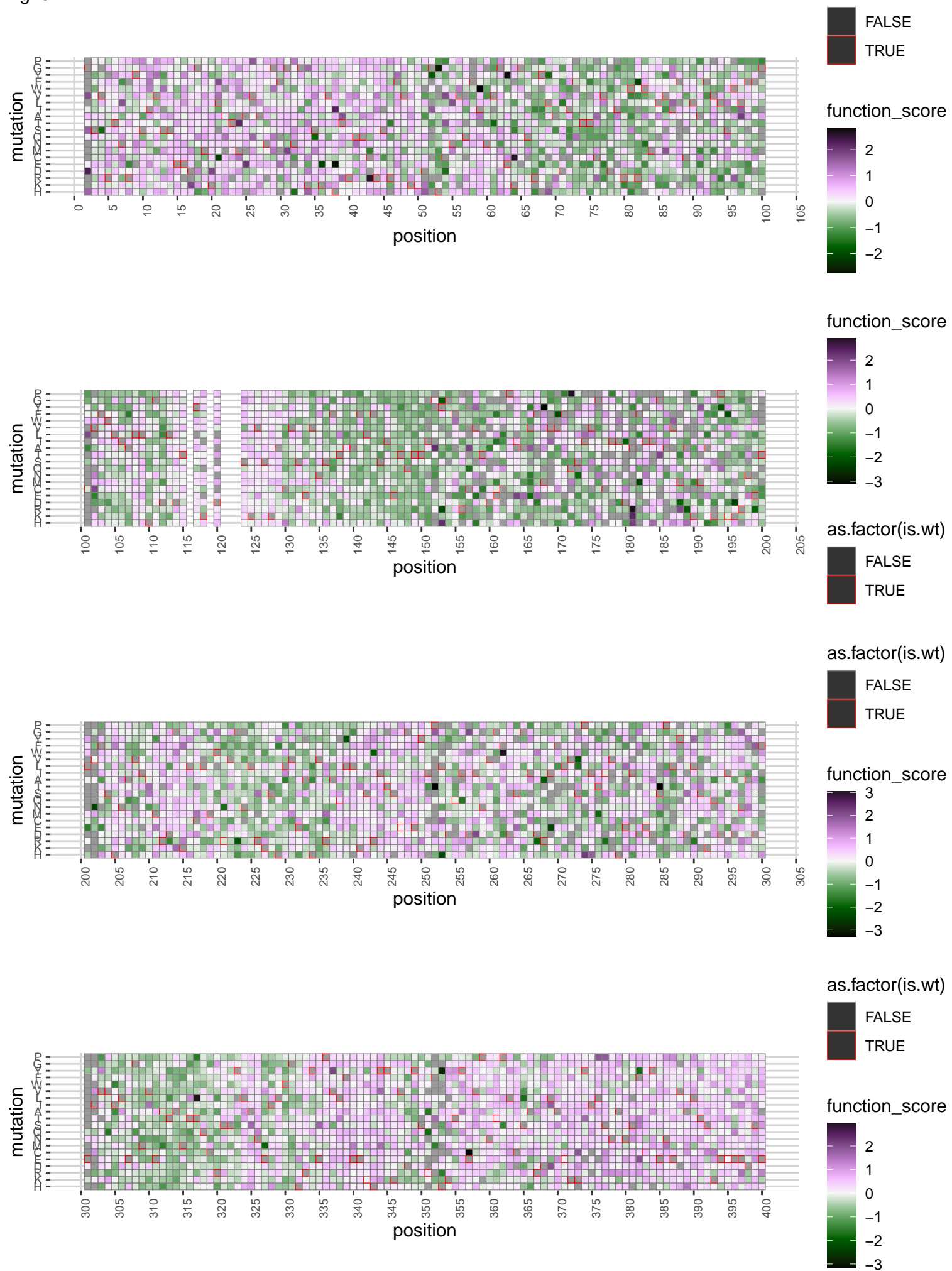

Supplement: Source data 1. [file elife-76903-data1.zip › SourceData/figure_output/Fig4c.pdf]

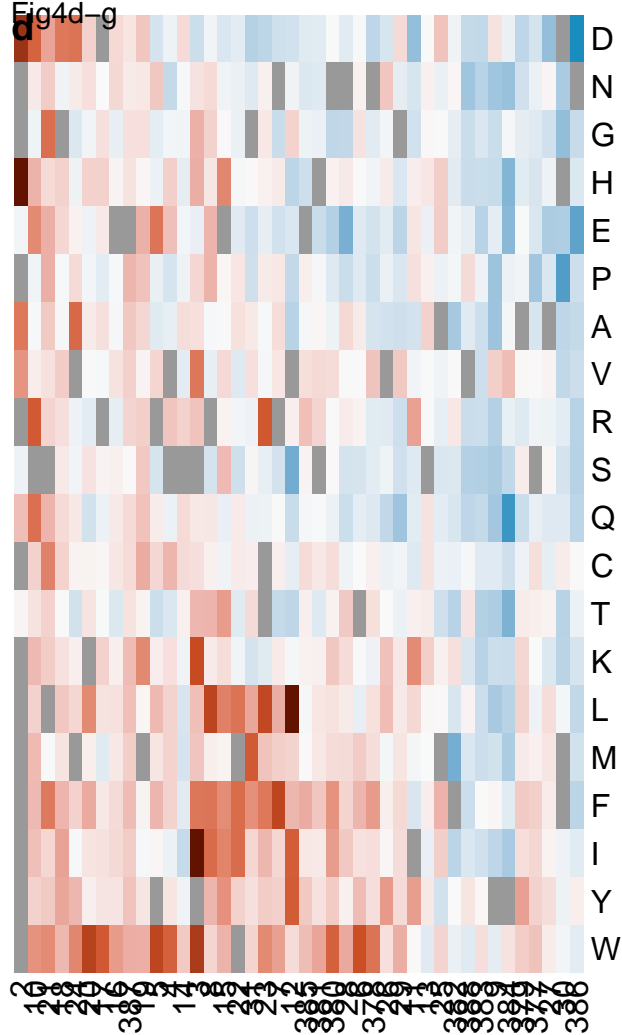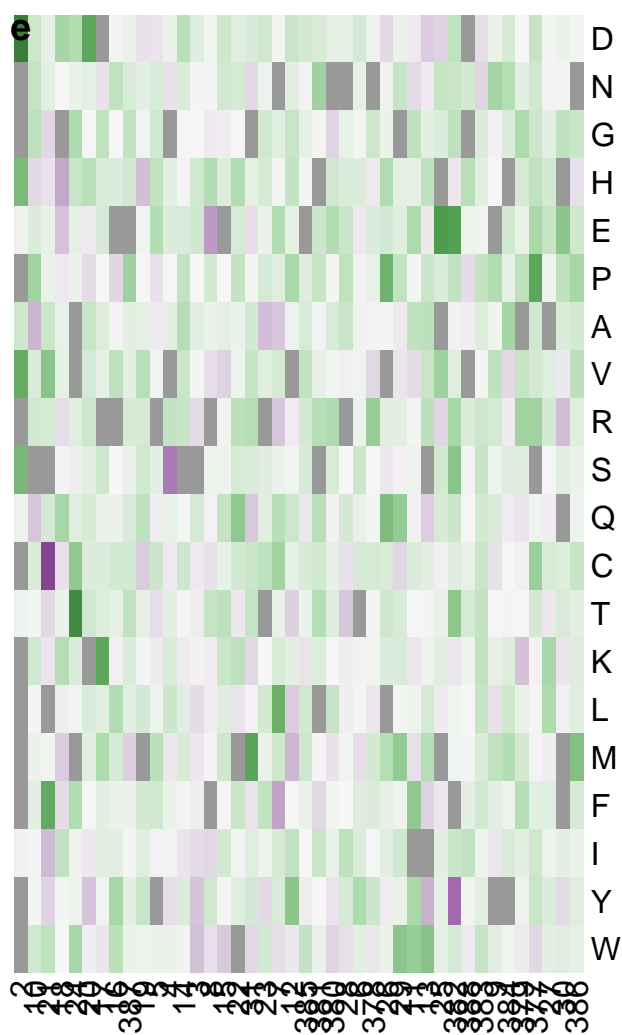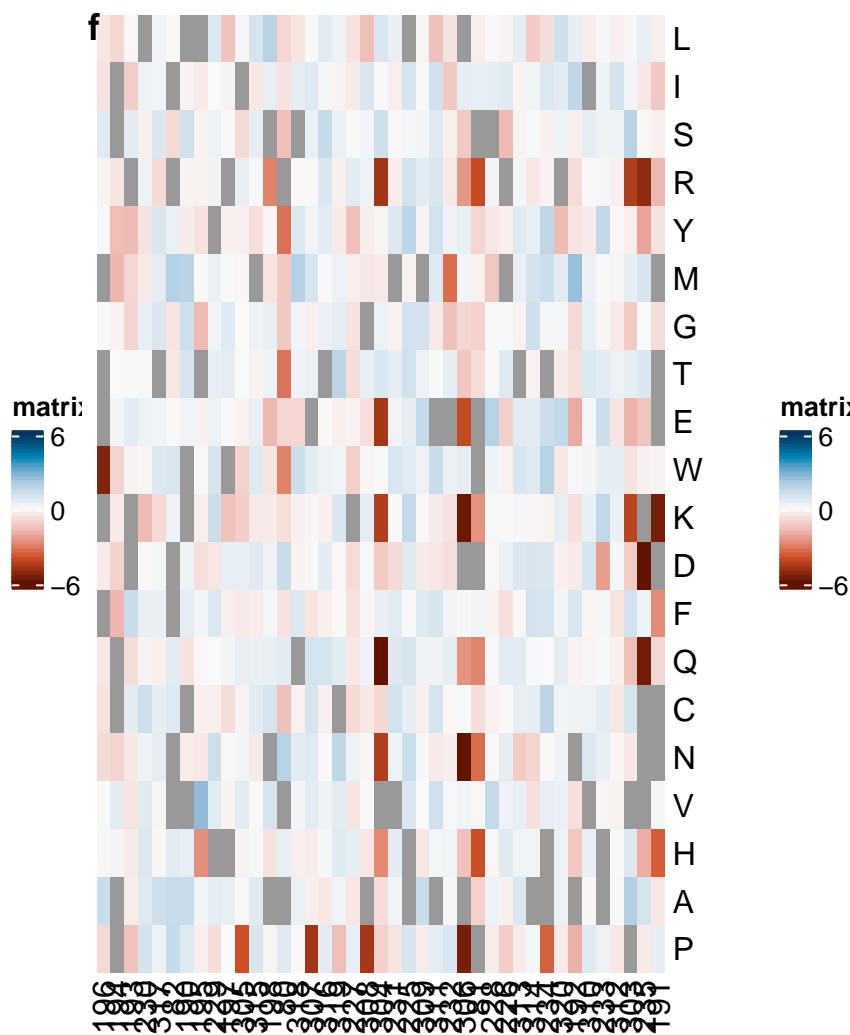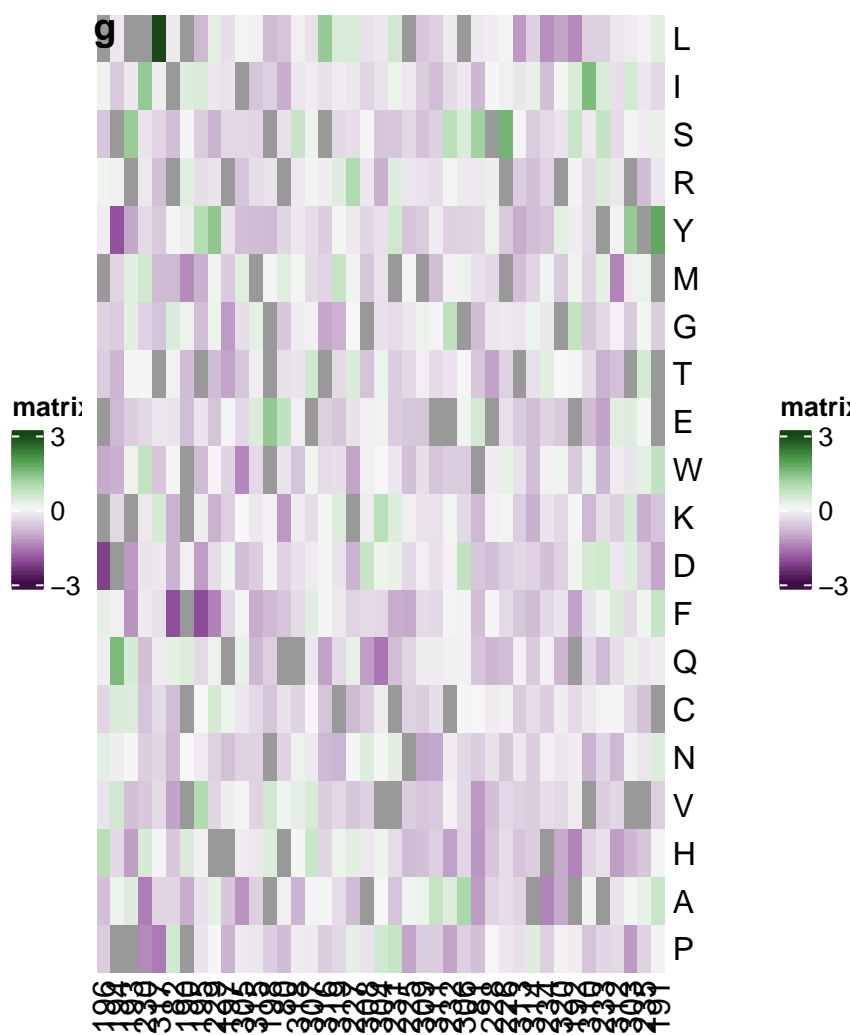

Supplement: Source data 1. [file elife-76903-data1.zip › SourceData/figure_output/Fig4d_g.pdf]

Fig5c

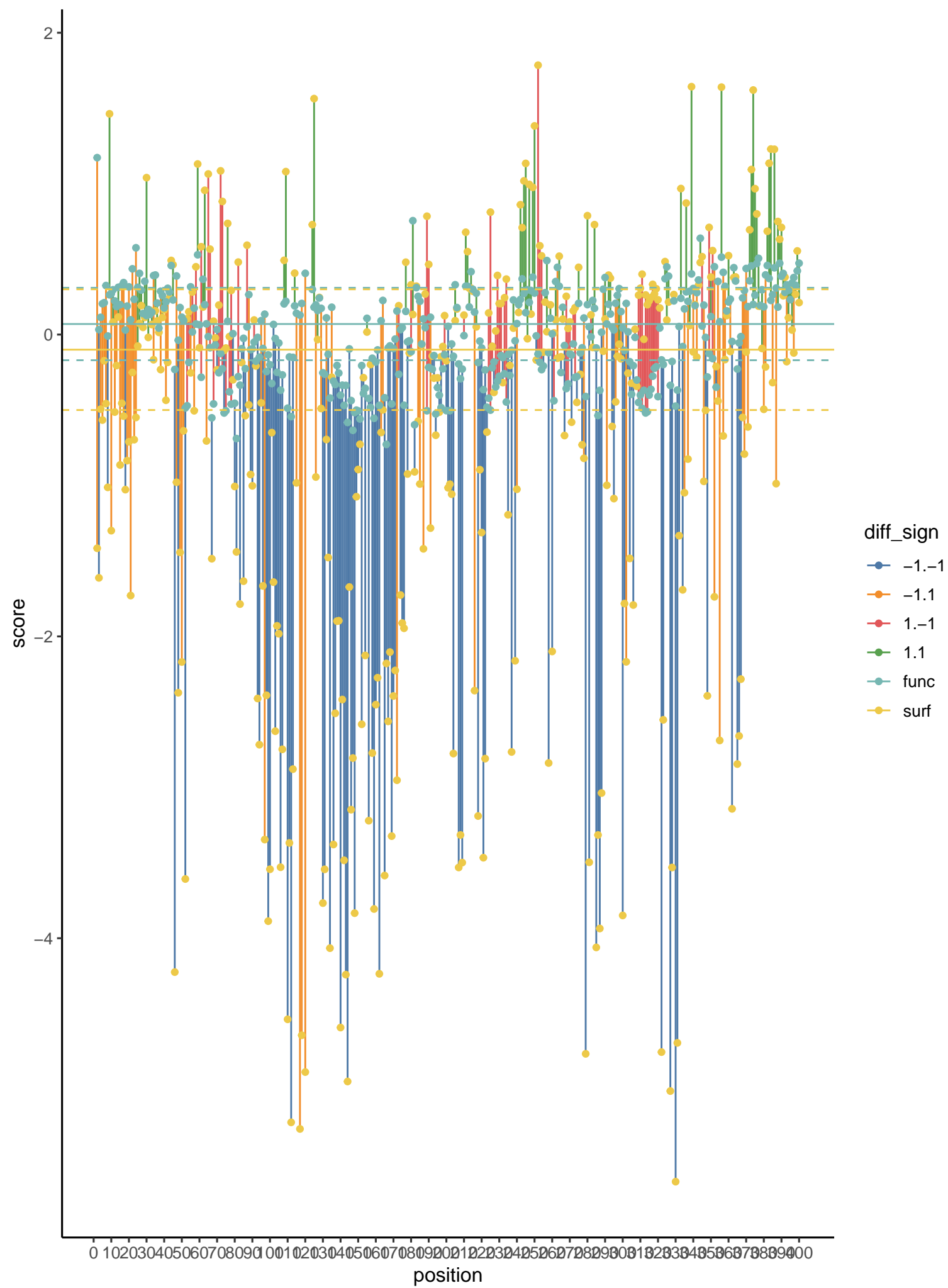

Supplement: Source data 1. [file elife-76903-data1.zip › SourceData/figure_output/Fig5c.pdf]

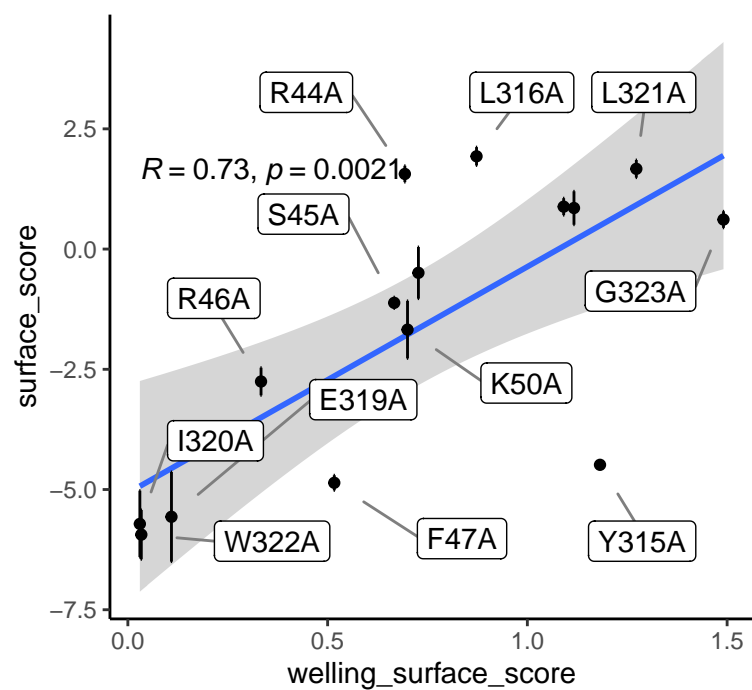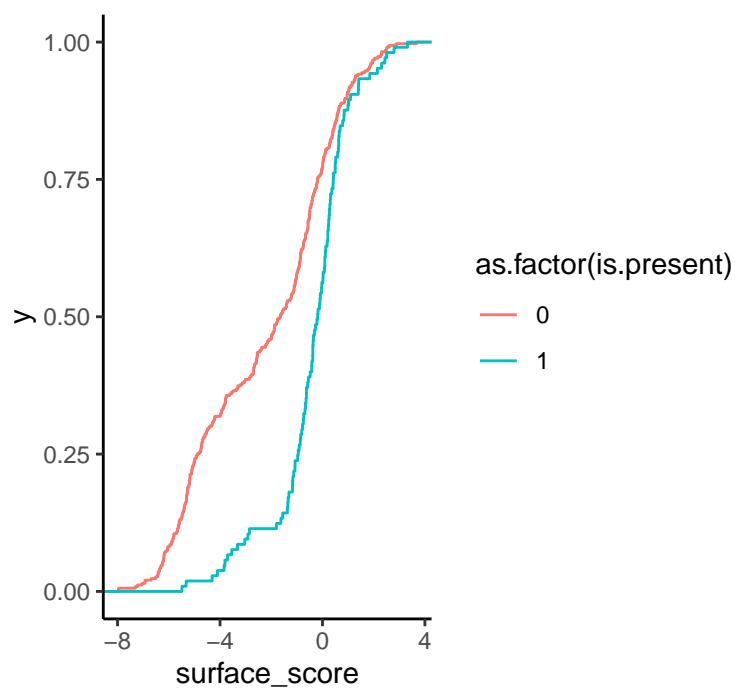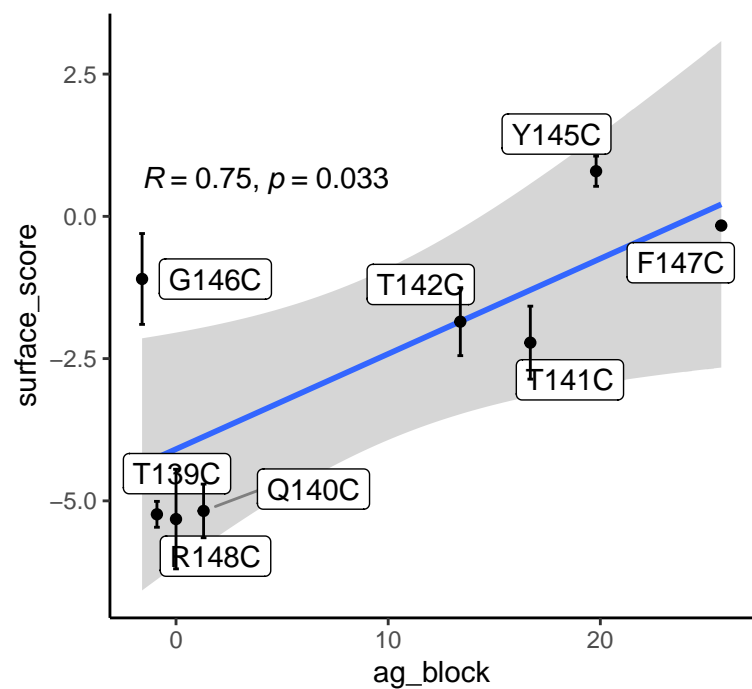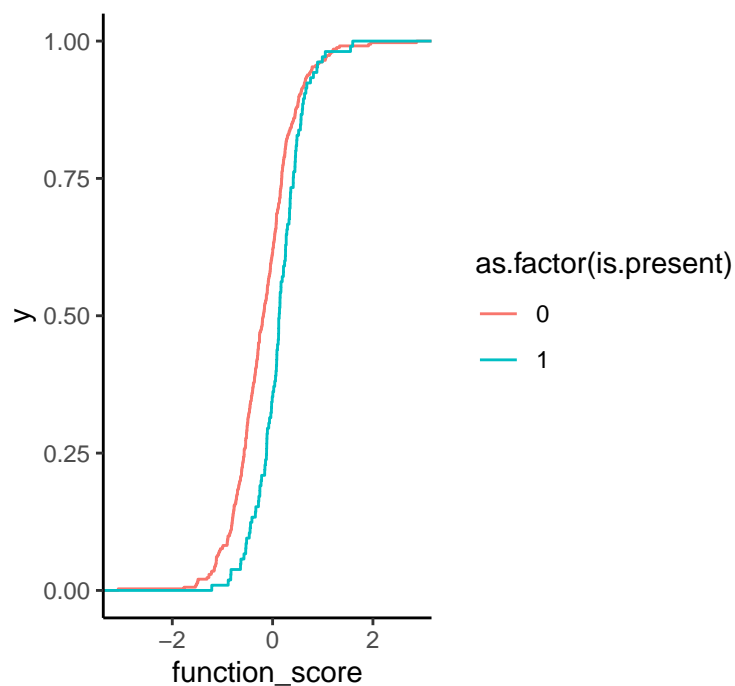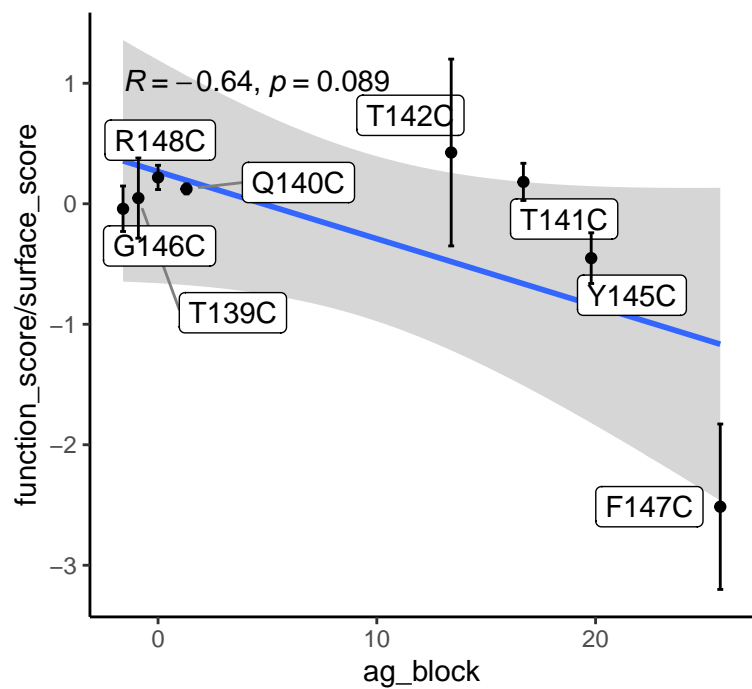

Supplement: Source data 1. [file elife-76903-data1.zip › SourceData/figure_output/Fig6.pdf]

Figure 7a-b

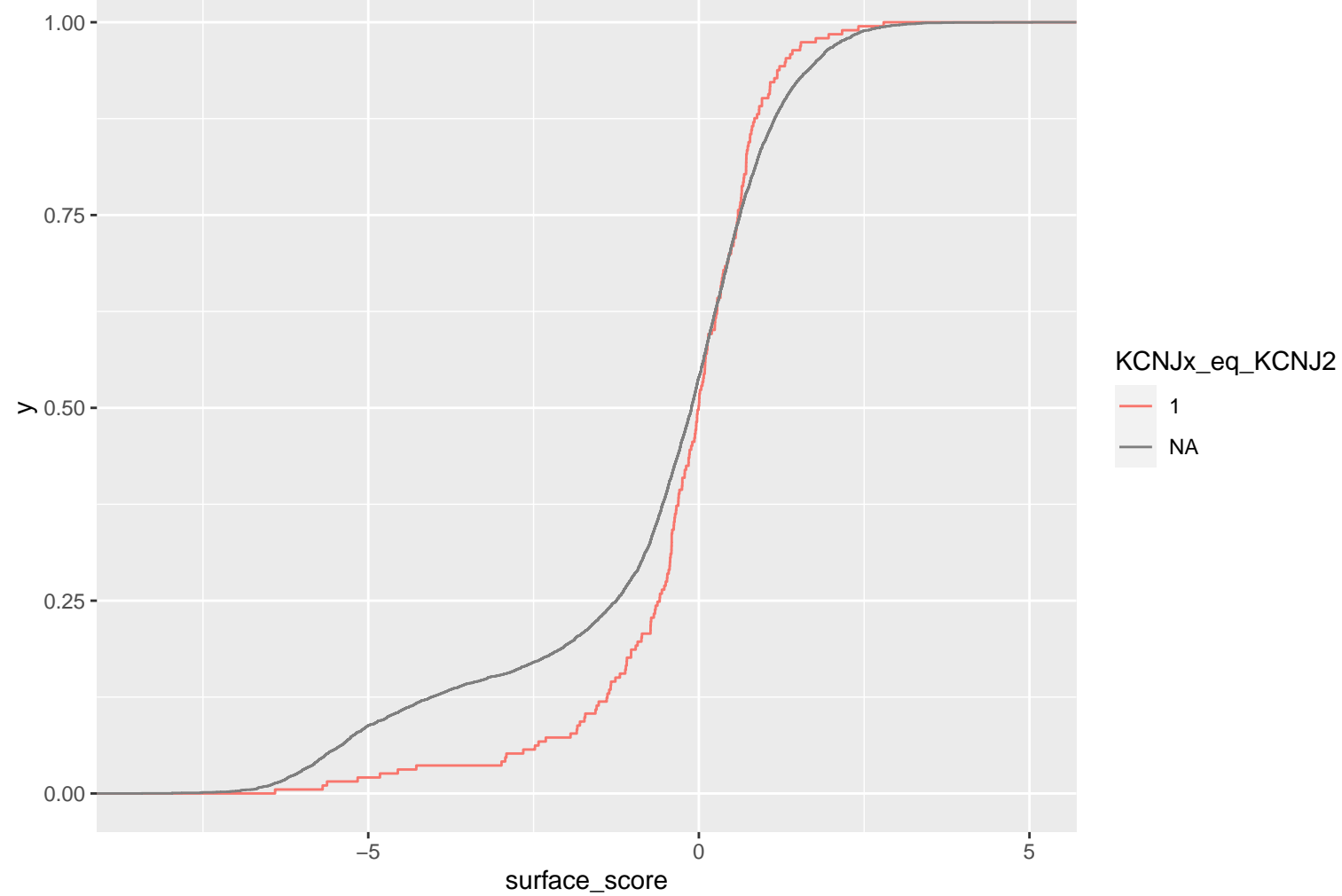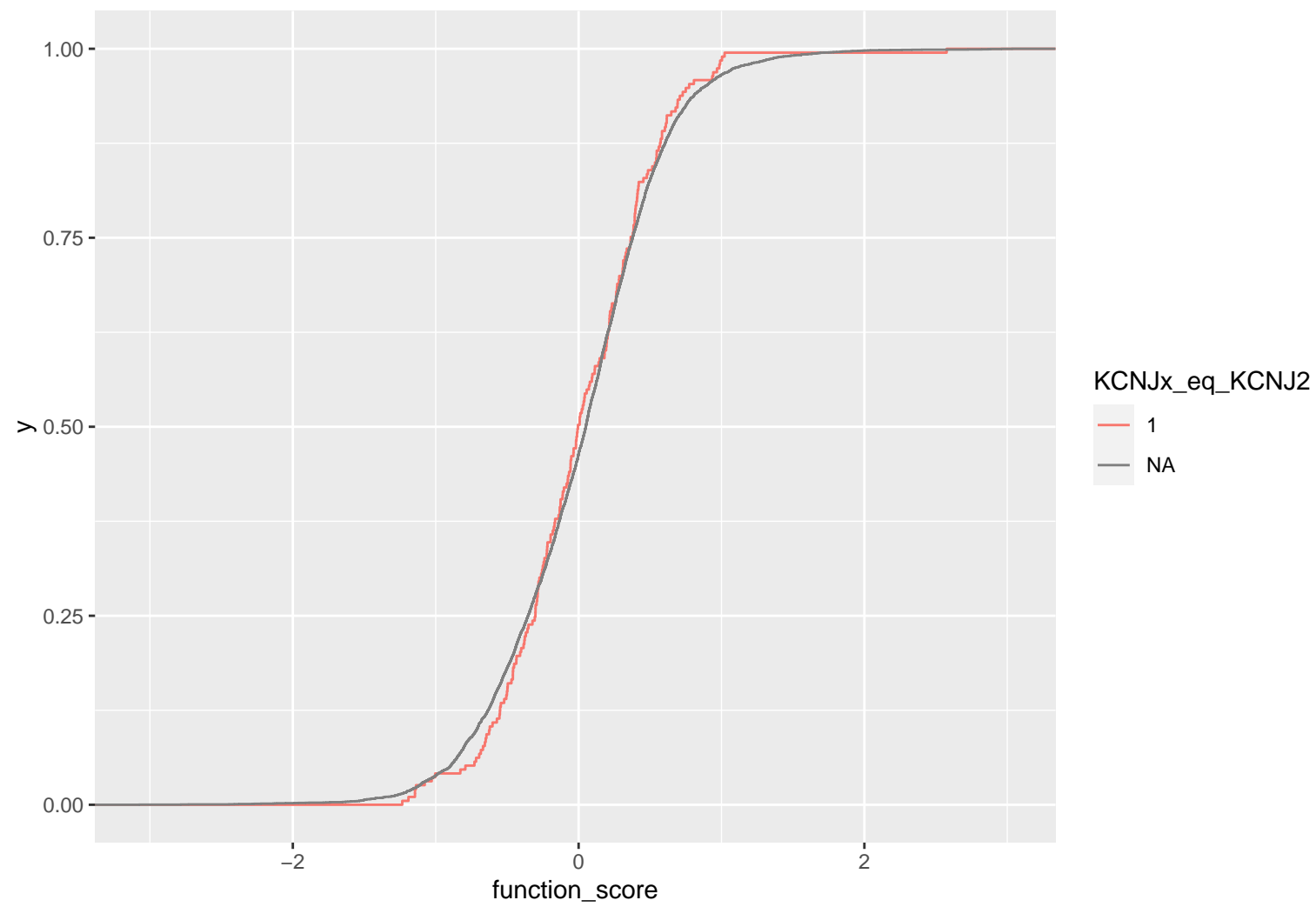

Supplement: Source data 1. [file elife-76903-data1.zip › SourceData/figure_output/Fig7ab.pdf]

Figure 7d-e  
cv\_df function by clinvar assignment

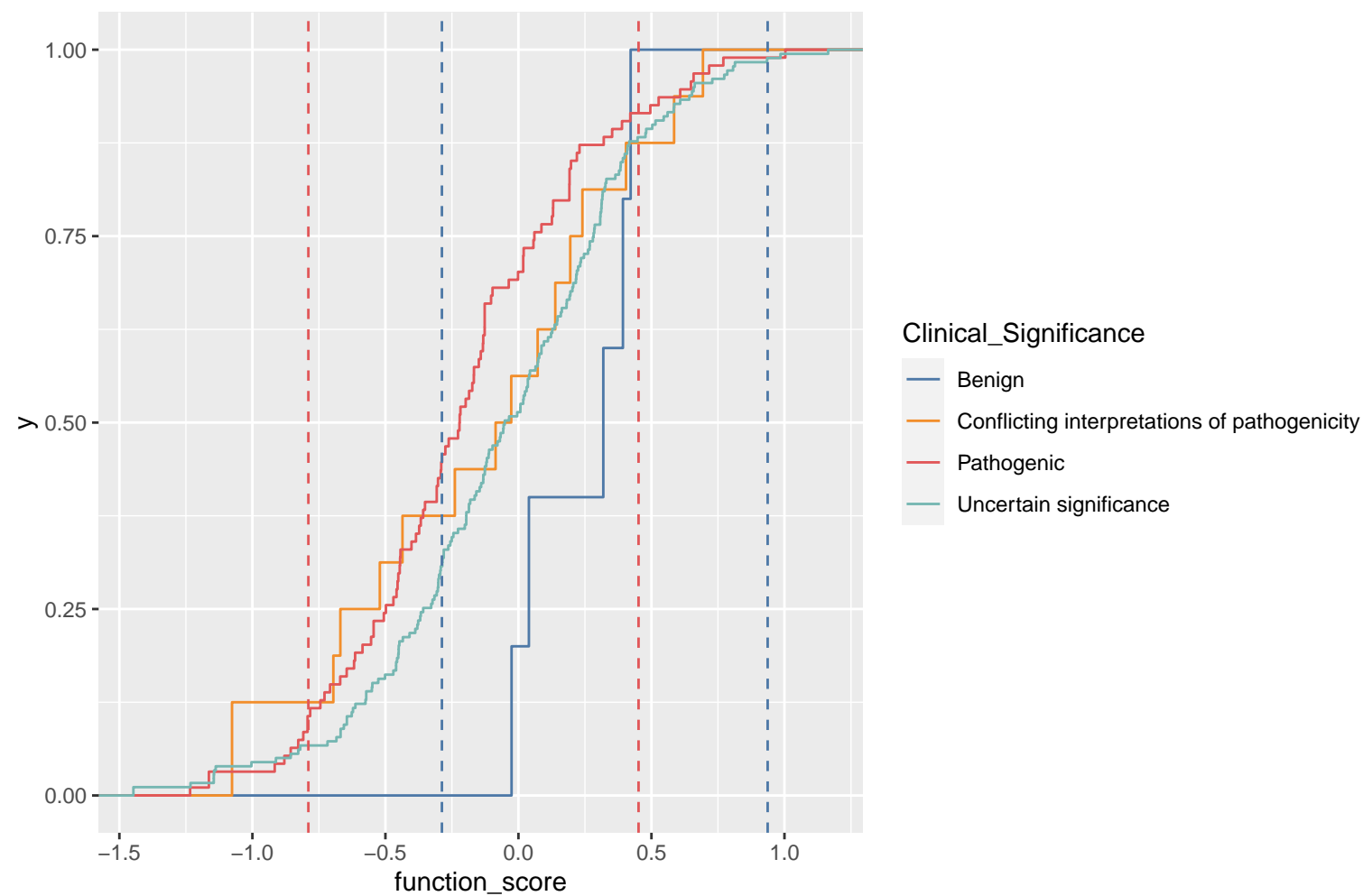

cv\_df function by clinvar assignment

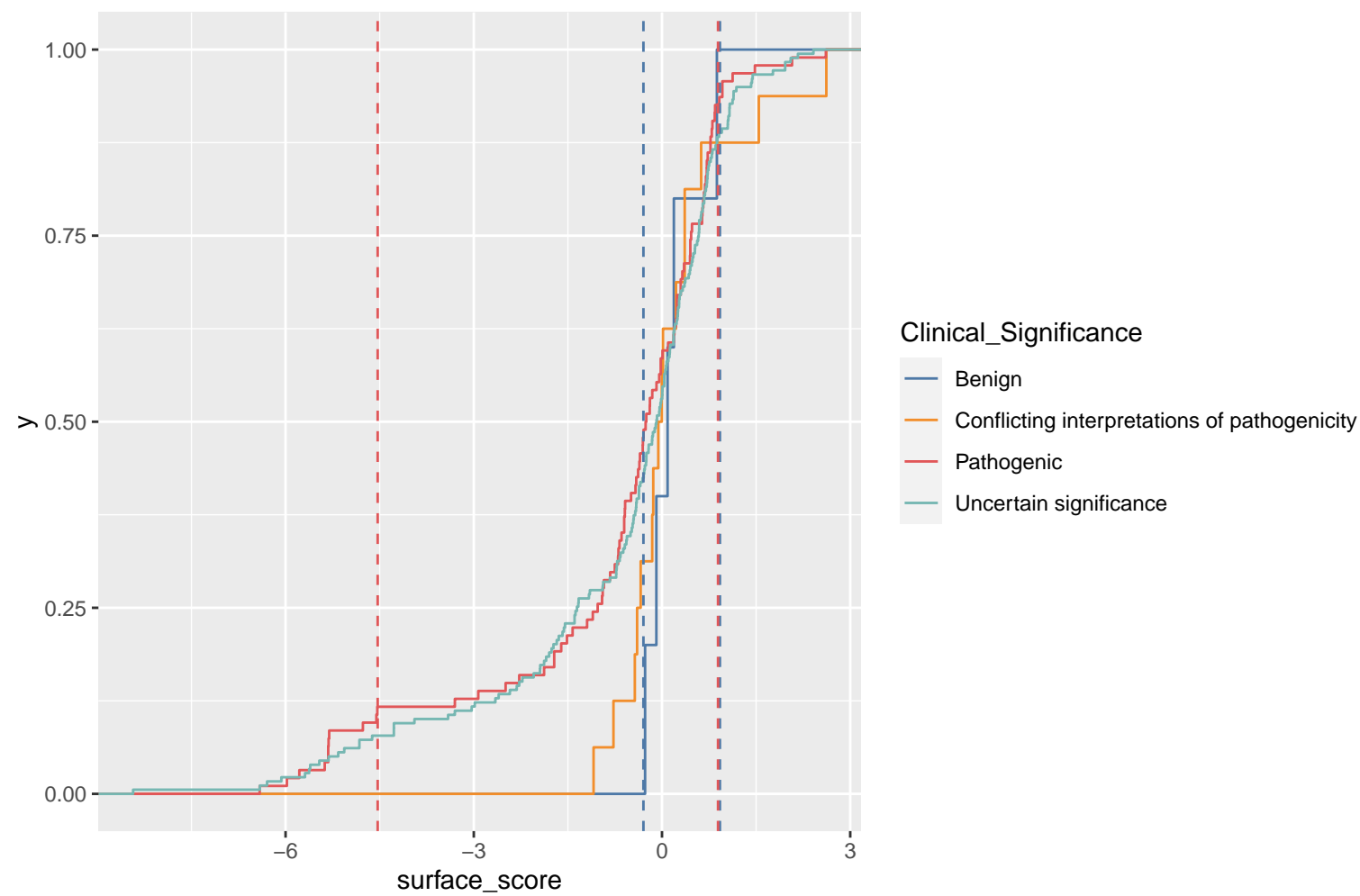

Supplement: Source data 1. [file elife-76903-data1.zip › SourceData/figure_output/Fig7de.pdf]

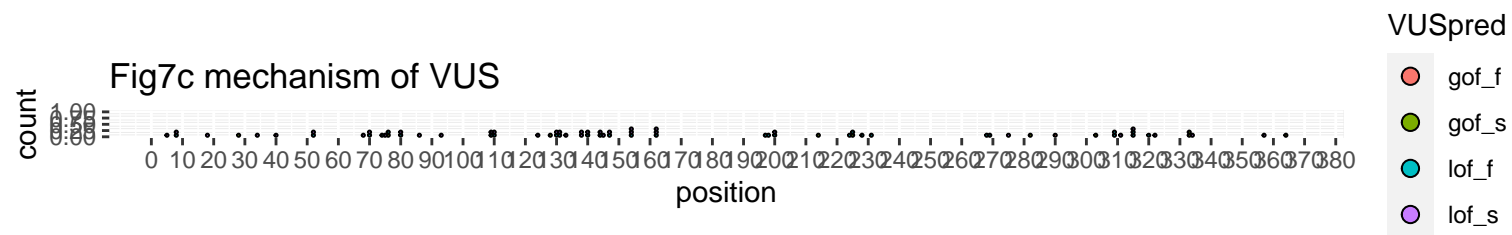

Supplement: Source data 1. [file elife-76903-data1.zip › SourceData/figure_output/Fig7c_vuspred_10per.pdf]

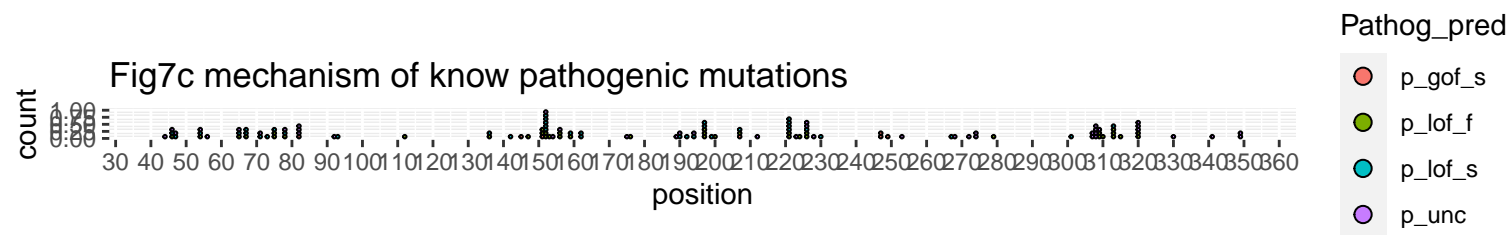

Supplement: Source data 1. [file elife-76903-data1.zip › SourceData/figure_output/Fig7c_pathopred_10per.pdf]

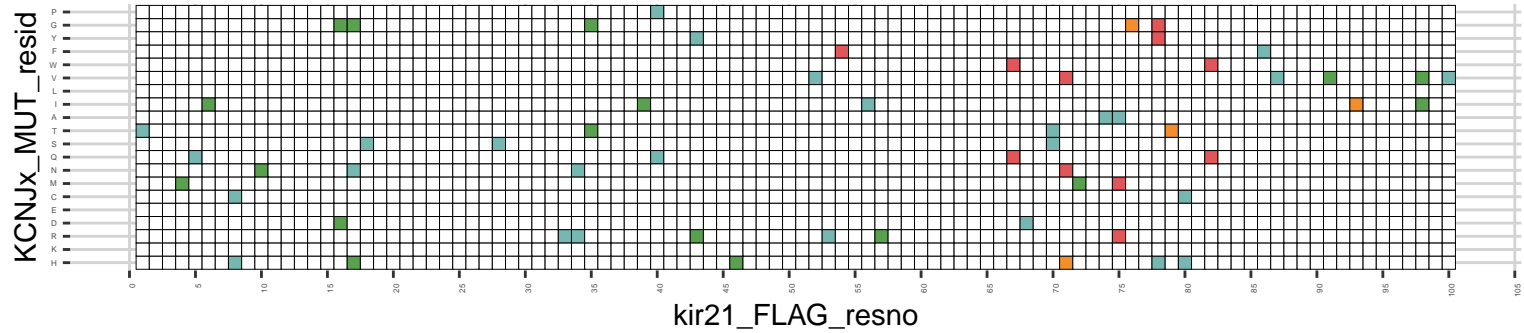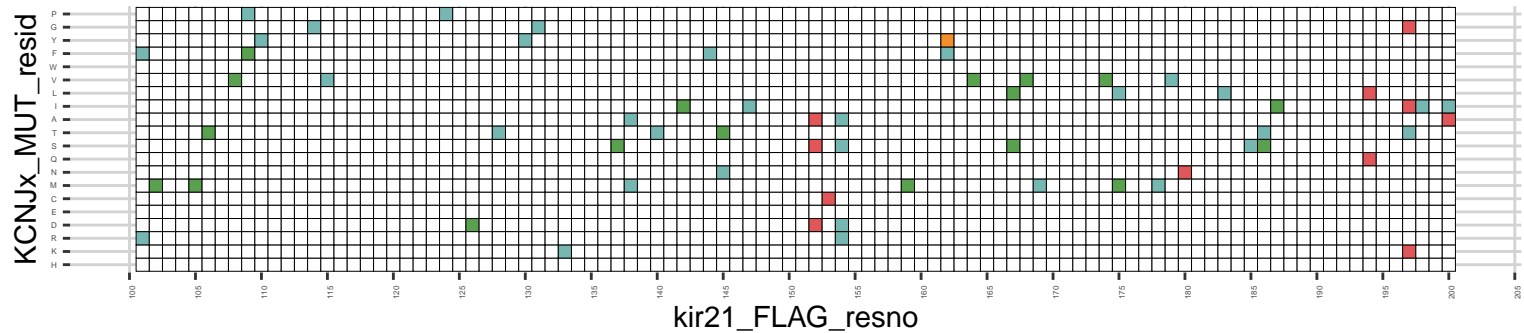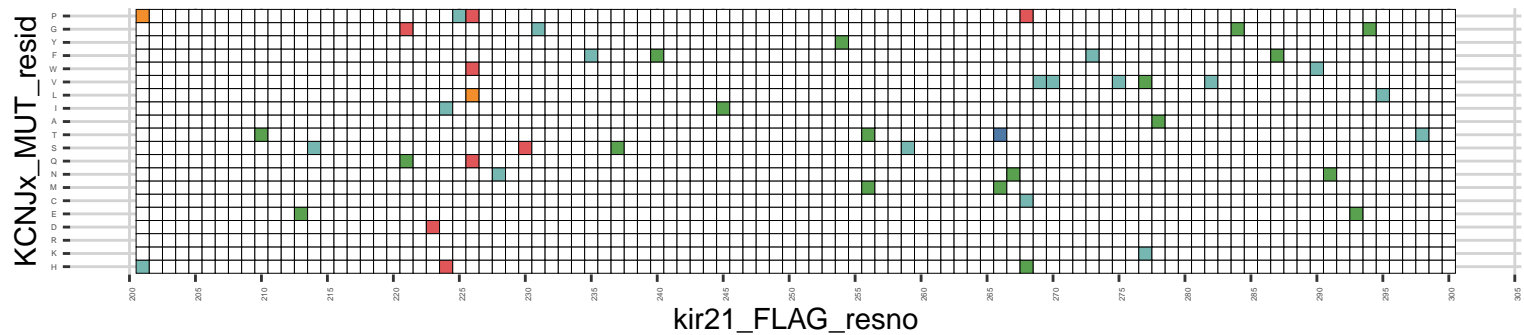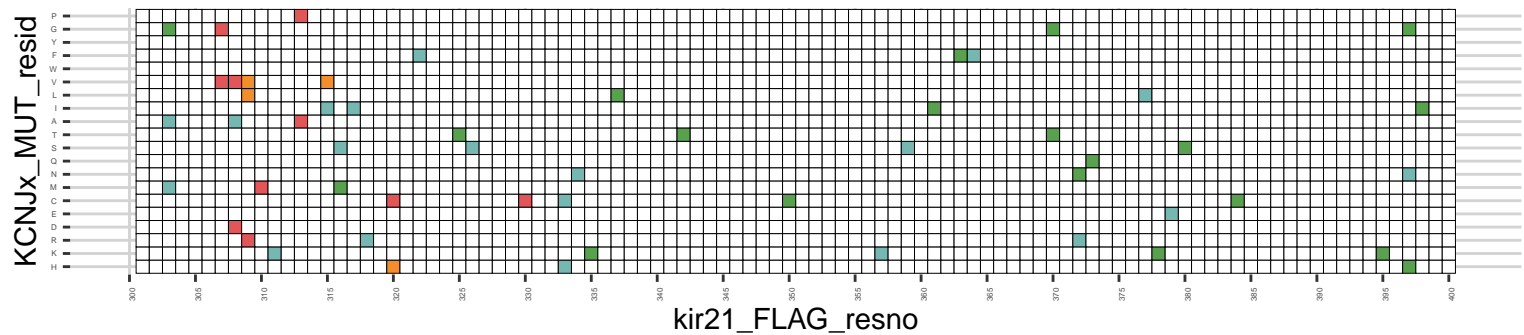

Supplement: Source data 1. [file elife-76903-data1.zip › SourceData/figure_output/Figure 1-figure supplement 1.pdf]

Figure 1—figure supplement 2a

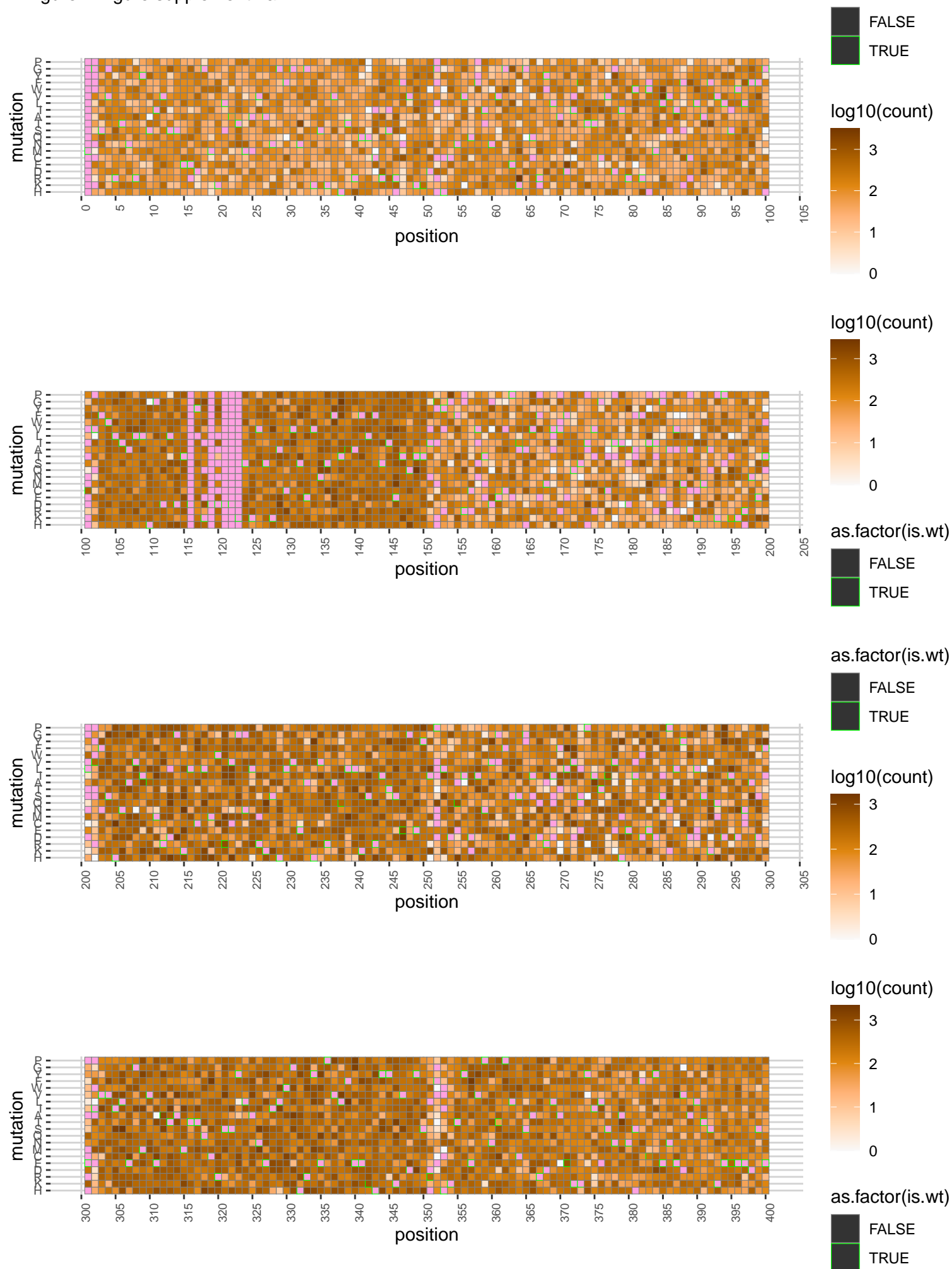

Supplement: Source data 1. [file elife-76903-data1.zip › SourceData/figure_output/Figure 1-figure supplement 2a.pdf]

Figure 1—figure supplement 2b

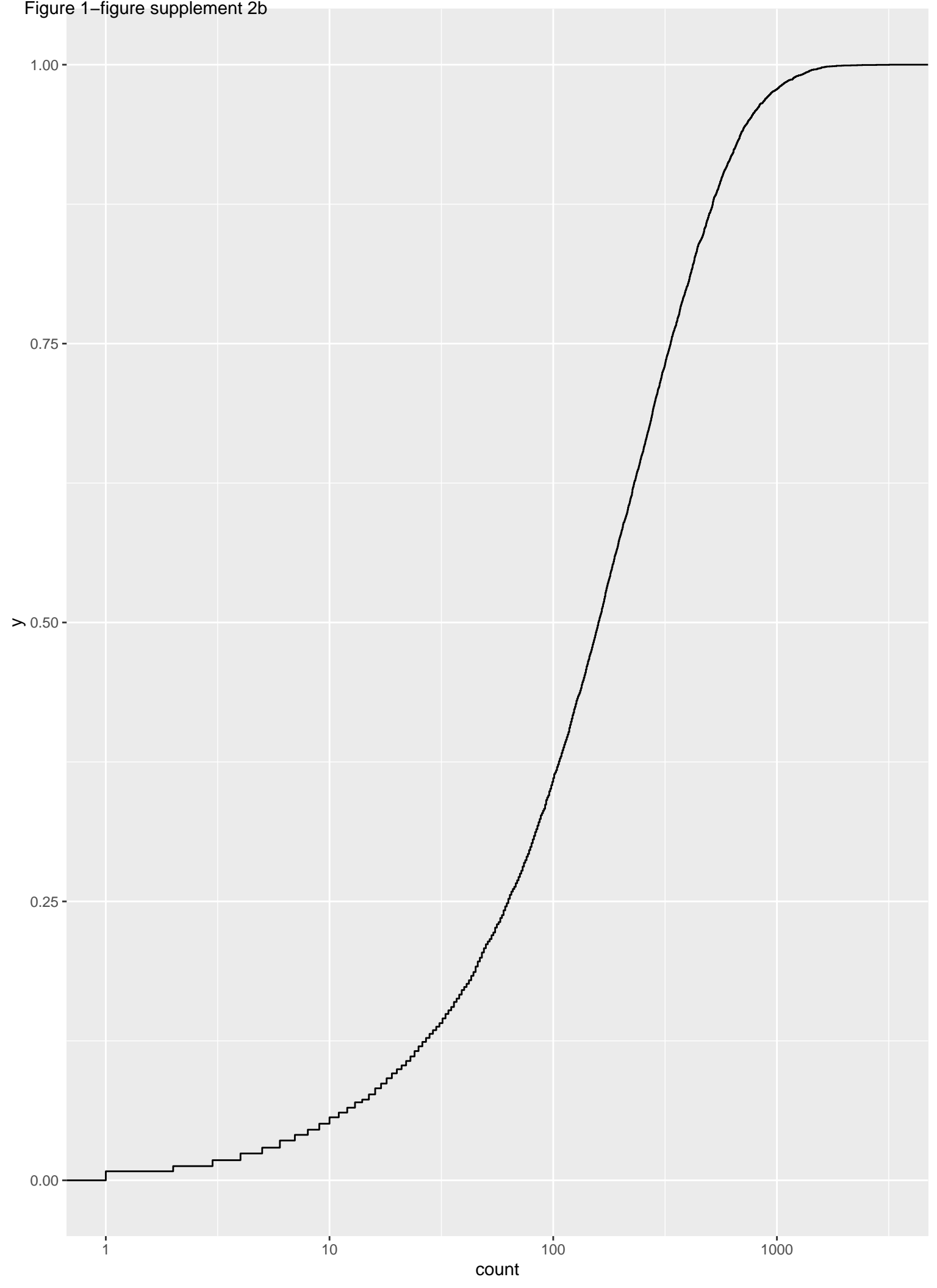

Supplement: Source data 1. [file elife-76903-data1.zip › SourceData/figure_output/Figure 1-figure supplement 2b.pdf]

Figure 2—figure supplement 1a

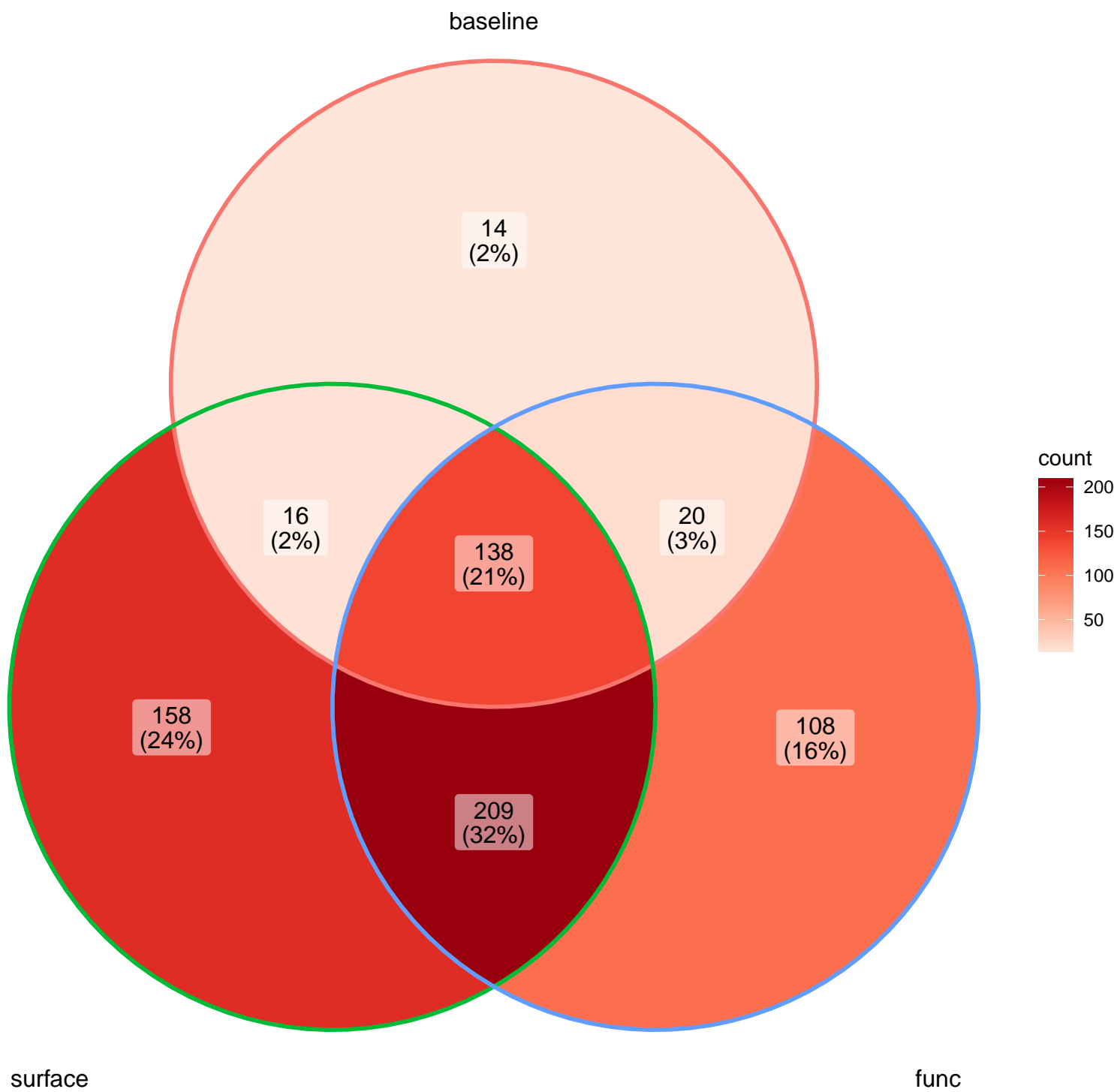

Supplement: Source data 1. [file elife-76903-data1.zip › SourceData/figure_output/Figure 2-figure supplement 1a.pdf]

Figure 2—figure supplement 2

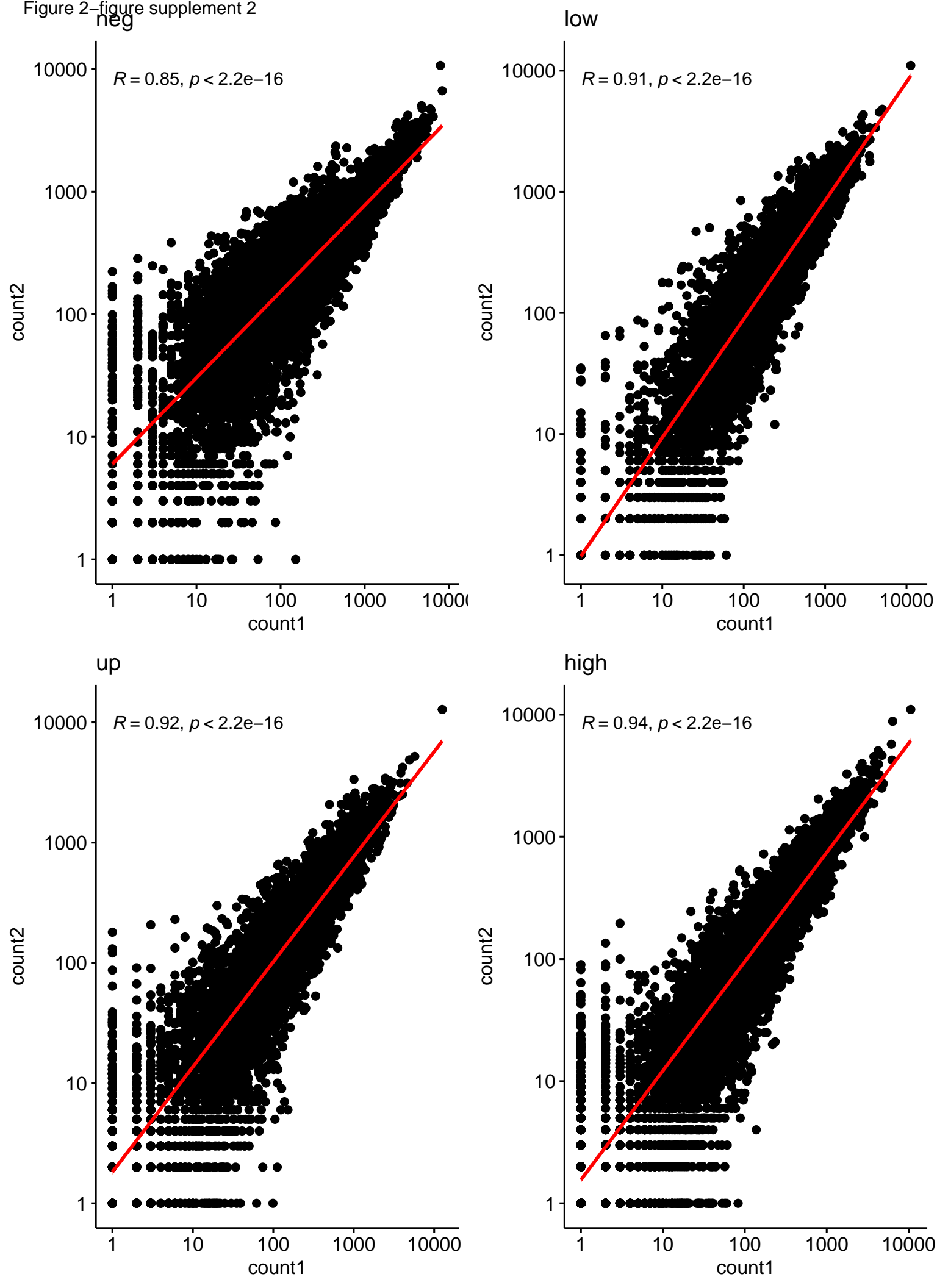

Supplement: Source data 1. [file elife-76903-data1.zip › SourceData/figure_output/Figure 2-figure supplement 2.pdf]

Figure 2—figure supplement 3

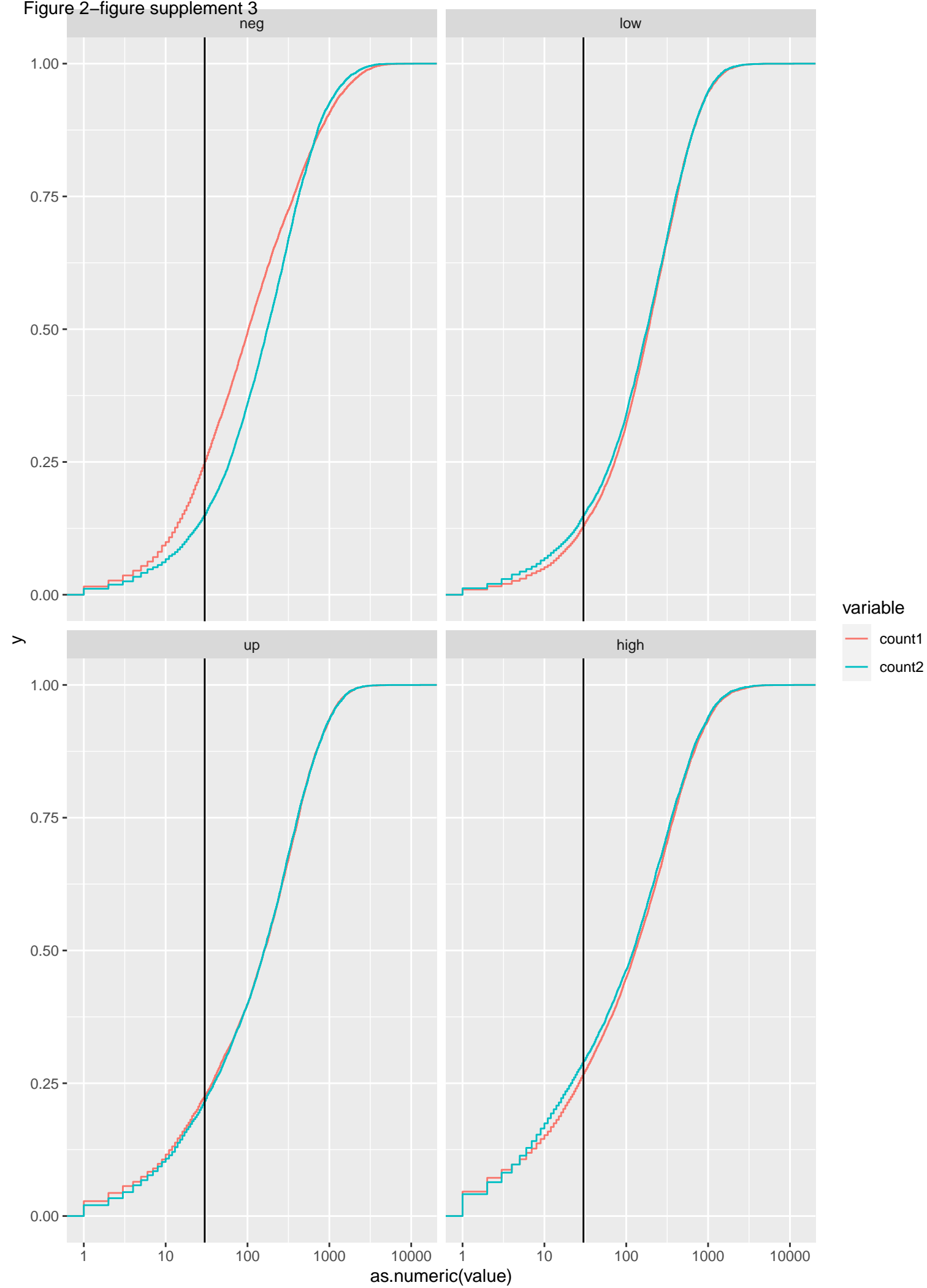

Supplement: Source data 1. [file elife-76903-data1.zip › SourceData/figure_output/Figure 2-figure supplement 3.pdf]

Figure 2—figure supplement 5a

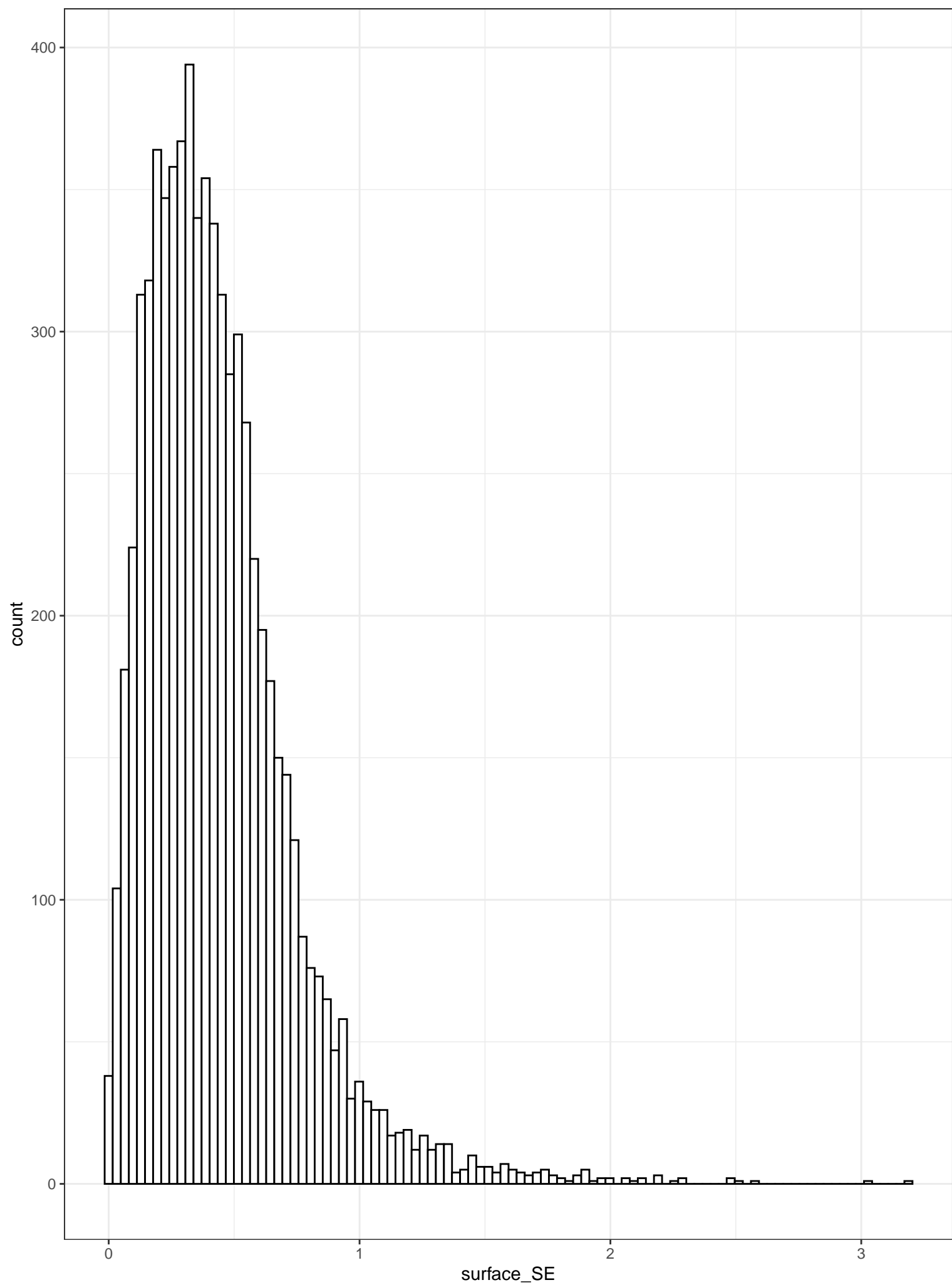

Supplement: Source data 1. [file elife-76903-data1.zip › SourceData/figure_output/Figure 2-figure supplement 5a.pdf]

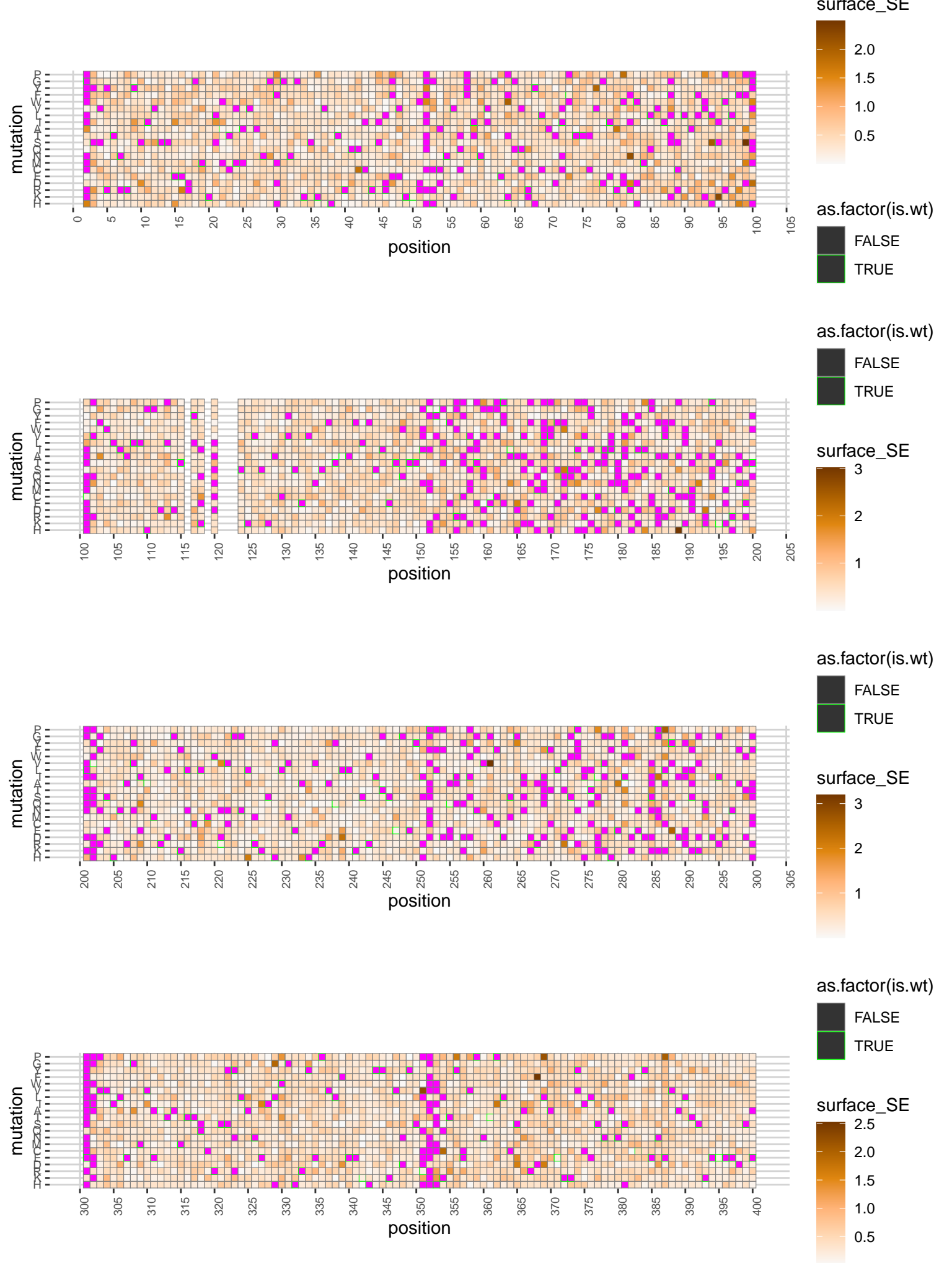

Supplement: Source data 1. [file elife-76903-data1.zip › SourceData/figure_output/Figure 2-figure supplement 5b.pdf]

Figure 4-figure supplement 1

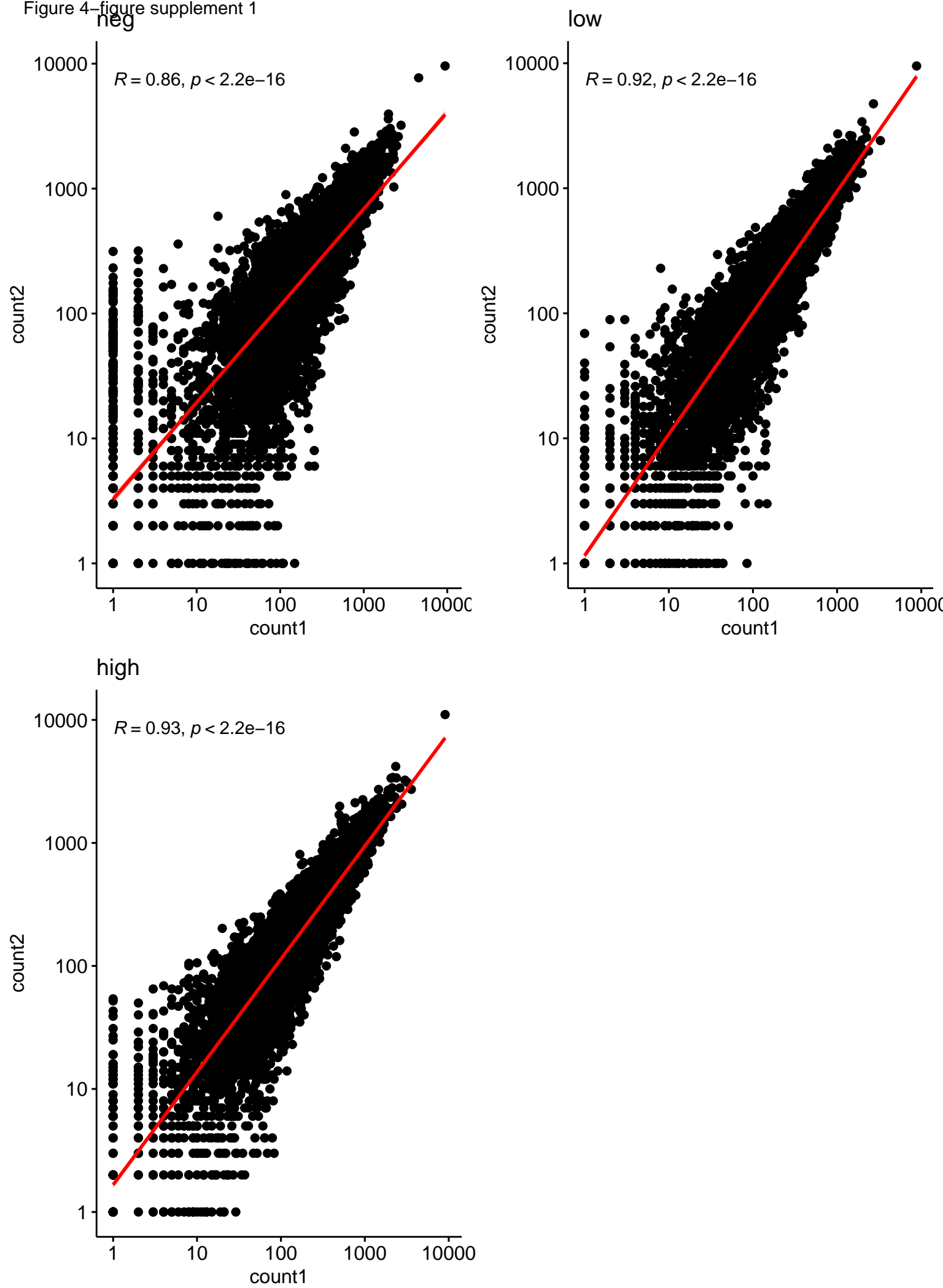

Supplement: Source data 1. [file elife-76903-data1.zip › SourceData/figure_output/Figure 4-figure supplement 1.pdf]

Figure 4—figure supplement 2

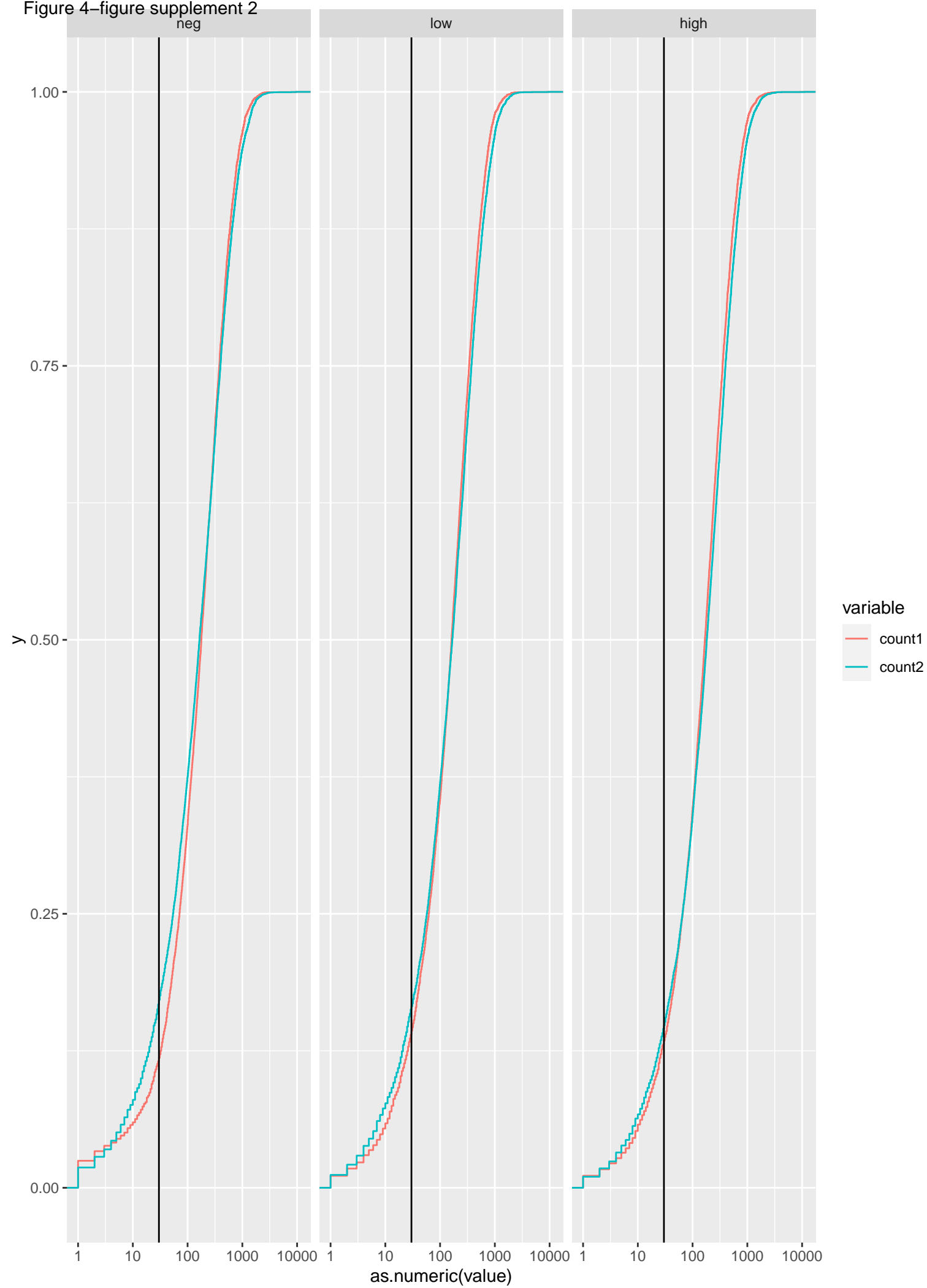

Supplement: Source data 1. [file elife-76903-data1.zip › SourceData/figure_output/Figure 4-figure supplement 2.pdf]

Figure 4—figure supplement 4a

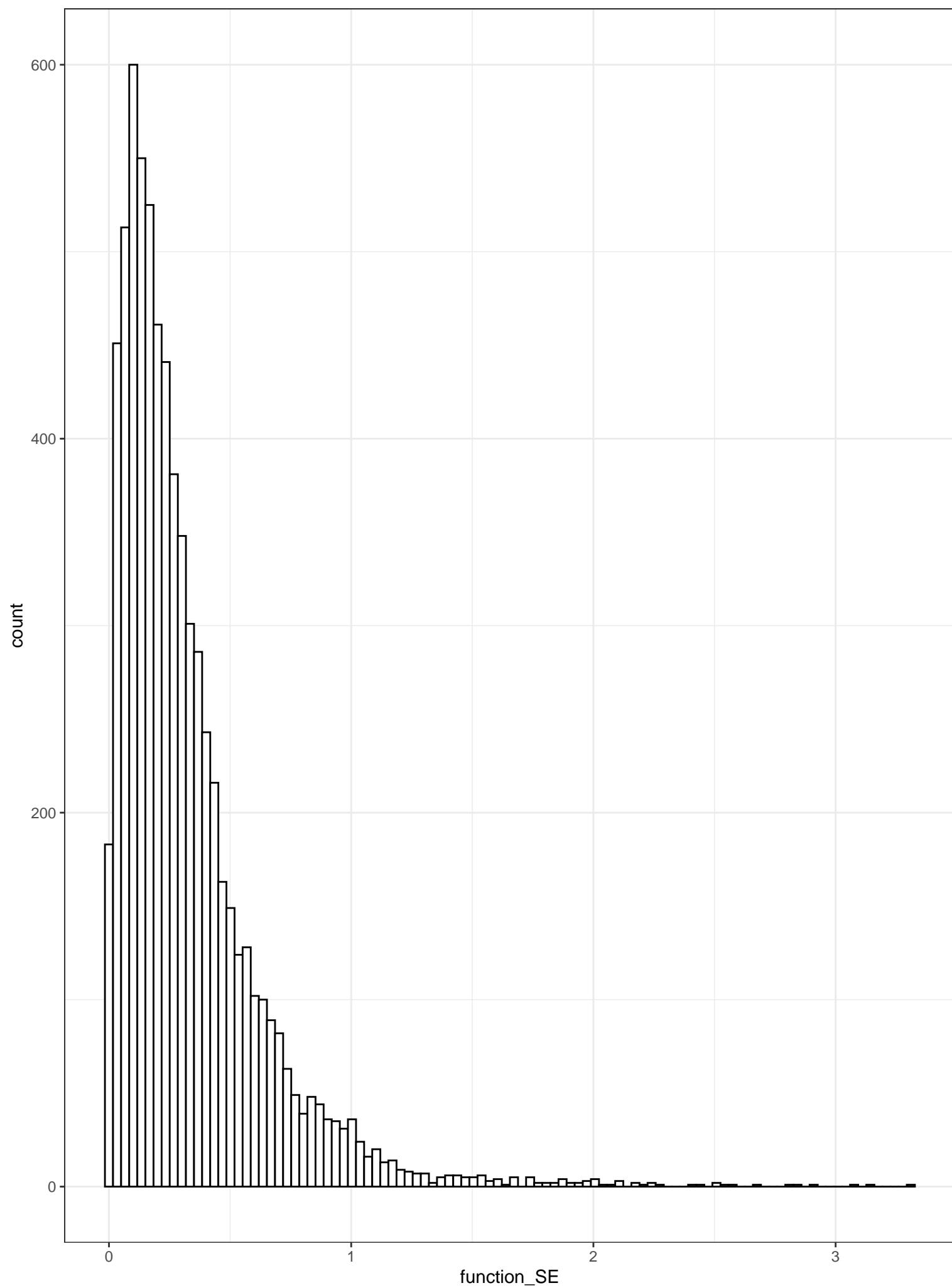

Supplement: Source data 1. [file elife-76903-data1.zip › SourceData/figure_output/Figure 4-figure supplement 4a.pdf]

```
as.factor(is.wt)
```

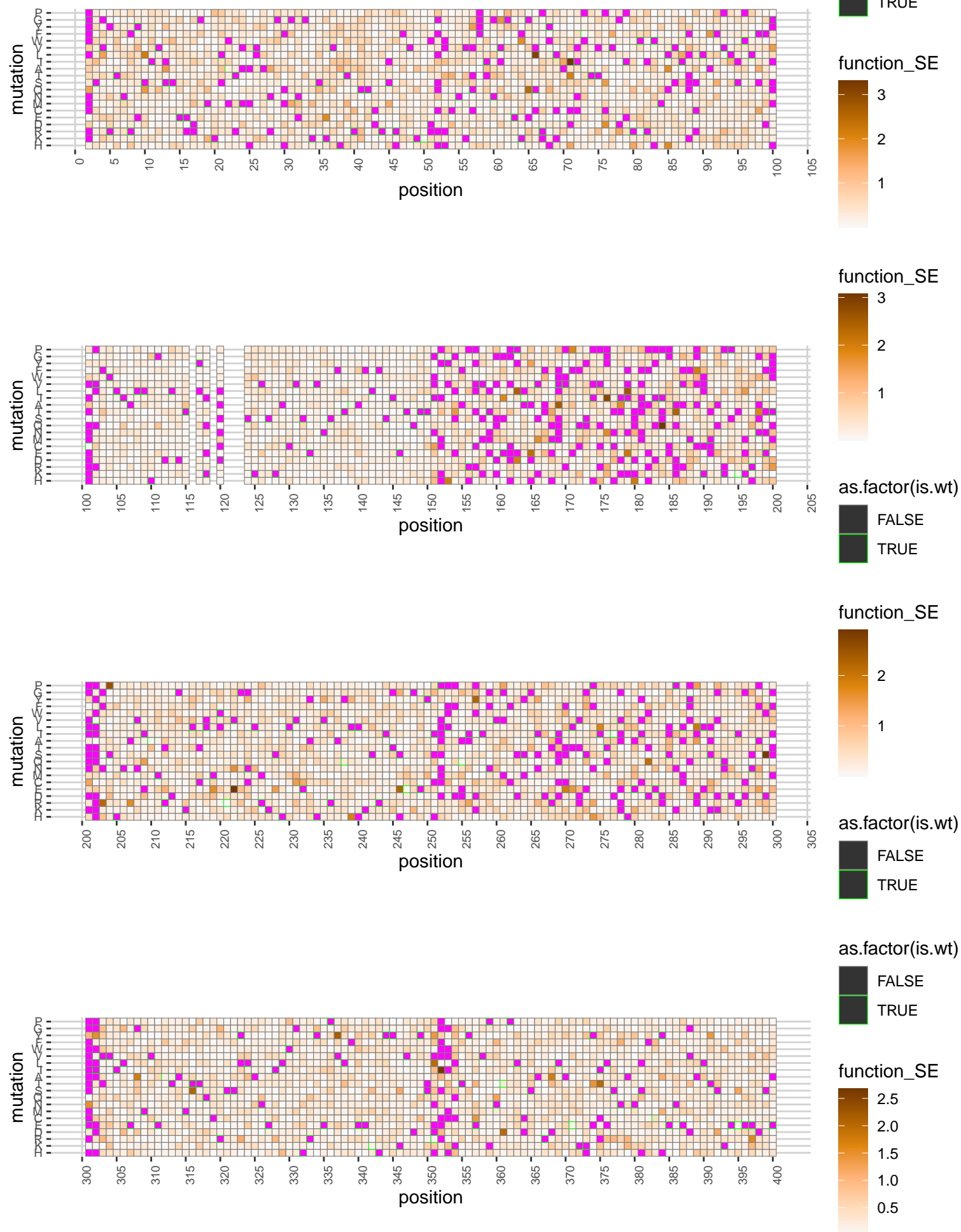

Supplement: Source data 1. [file elife-76903-data1.zip › SourceData/figure_output/Figure 4-figure supplement 4b.pdf]

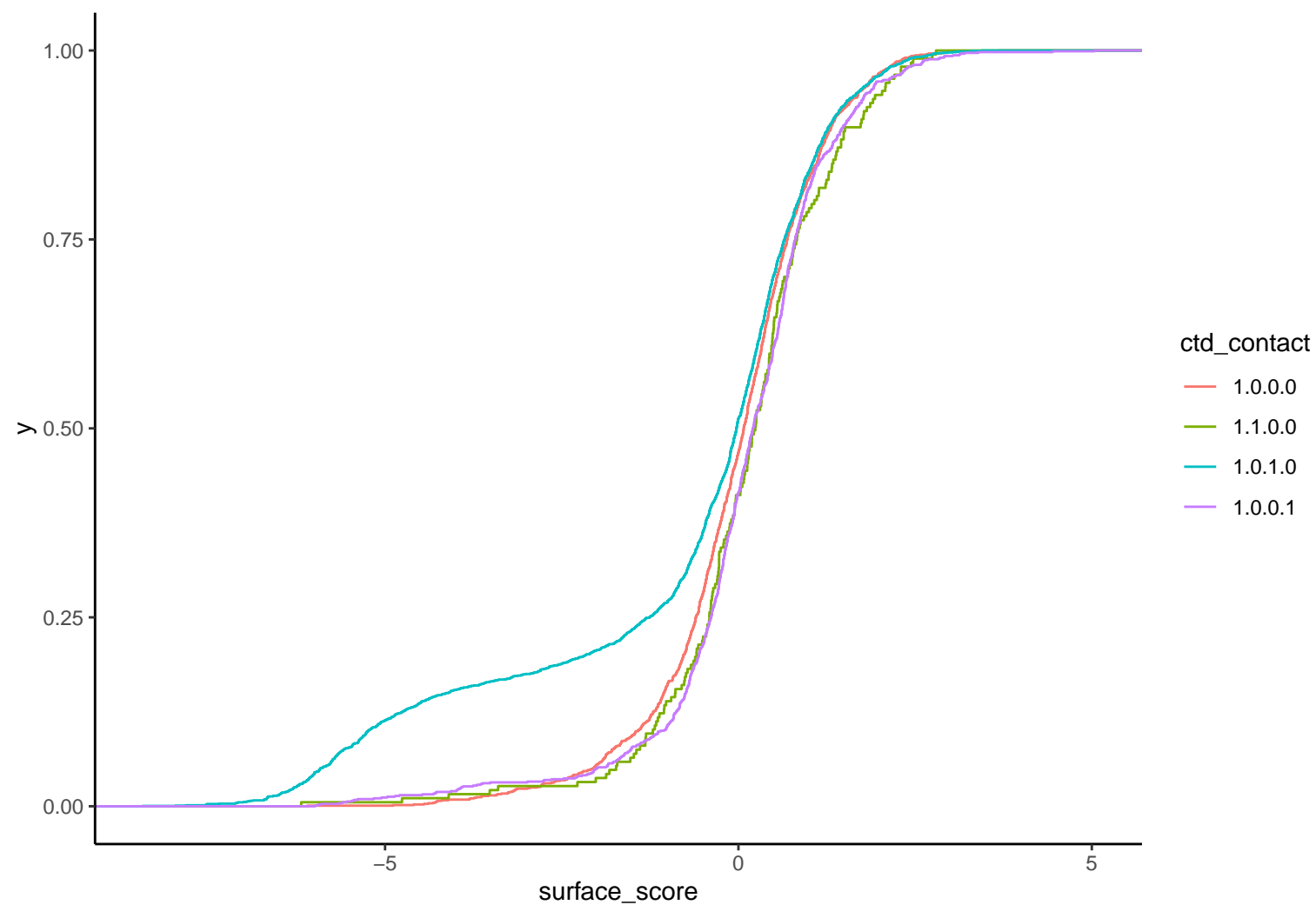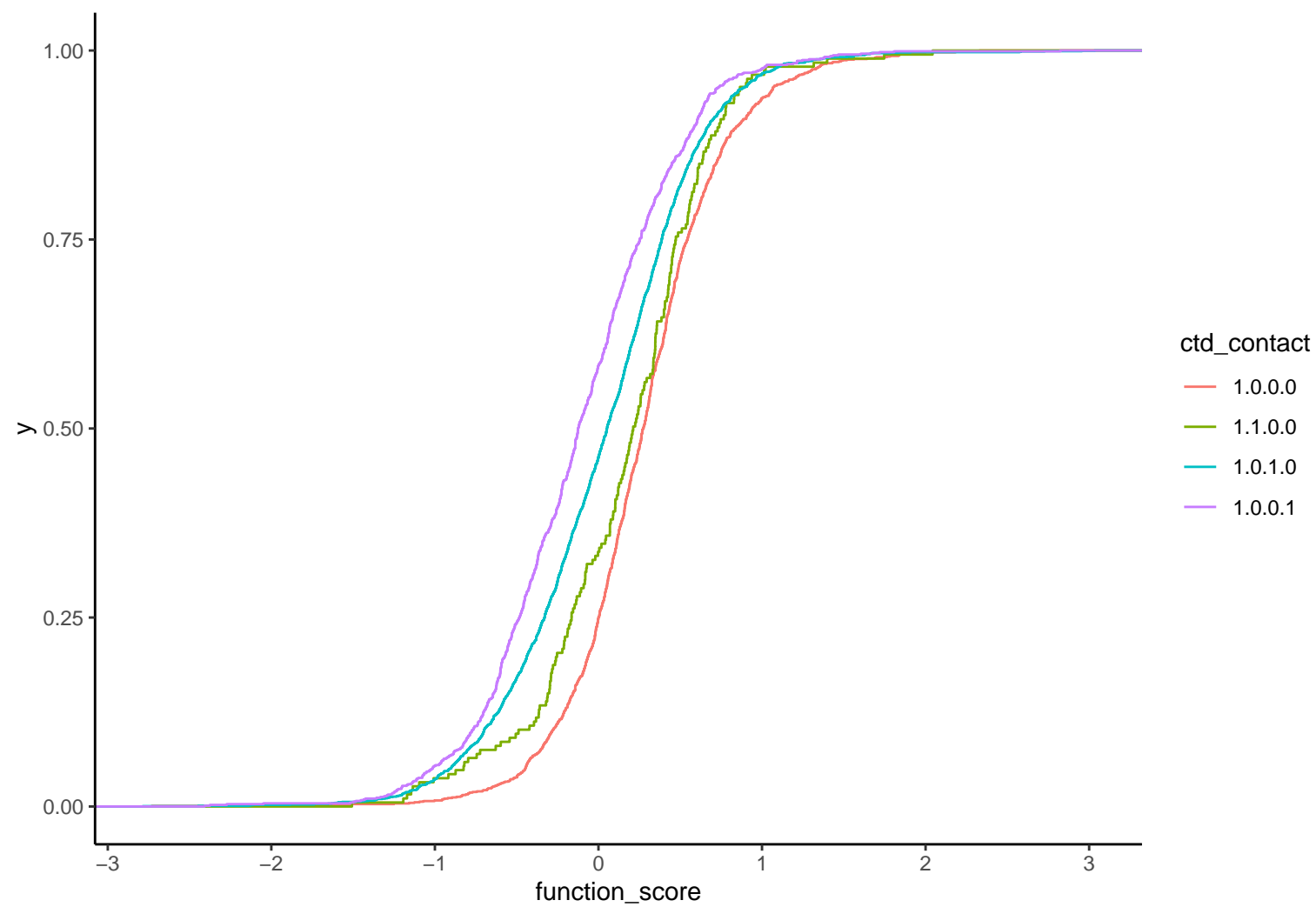

Supplement: Source data 1. [file elife-76903-data1.zip › SourceData/figure_output/Figure 5-figure supplement 1.pdf]
